# Synthesis, crystal structure and hydrogenation properties of Mg_*x*_Li_3 − *x*_B_48 − *y*_ (*x* = 1.11, *y* = 0.40)

**DOI:** 10.1107/S2056989023009969

**Published:** 2024-01-01

**Authors:** Nazar Pavlyuk, Viktoria Milashius, Vasyl Kordan, Volodymyr Pavlyuk

**Affiliations:** a Department of Inorganic Chemistry, Ivan Franko National University of Lviv, Kyryla i Mefodiya str., 6, 79005, Lviv, Ukraine; National Taras Shevchenko University of Kyiv, Ukraine

**Keywords:** crystal structure, inter­metallic compounds, hydrides

## Abstract

The ternary magnesium/lithium boride, Mg_*x*_Li_3 − *x*_B_48 − *y*_ (*x* = 1.11, *y* = 0.40, idealized formula MgLi_2_B_48_), crystallizes as its own structure type in *P*4_3_2_1_2, which is closely related to the structural family comprising α-AlB_12_, Be_0.7_Al_1.1_B_22_ and tetra­gonal β-boron. The asymmetric unit of title structure contains two statistical mixtures Mg/Li in Wyckoff sites 8*b* with relative occupancies Mg:Li = 0.495 (9):0.505 (9) and 4*a* with Mg:Li = 0.097 (8):0.903 (8). The boron atoms occupy 23 8*b* sites and two 4*a* sites. One of the latter sites has a partial occupancy factor of 0.61 (2). Both unique Mg/Li atoms adopt a twelvefold coordination environment in the form of truncated tetra­hedra (Laves polyhedra). These polyhedra are connected by triangular faces to four [B_12_] icosa­hedra. The boron atoms exhibit four kinds of polyhedra, namely penta­gonal pyramid (coordination number CN = 6), distorted tetra­gonal pyramid (CN = 5), bicapped hexa­gon (CN = 8) and gyrobifastigium (CN = 8). At the gas hydrogenation of MgLi_2_B_48_ alloy, formation of the eutectic composite hydride LiBH_4_+Mg(BH_4_)_2_ and amorphous boron is observed. In the temperature range 543–623 K, the hydride eutectics decompose, forming MgH_2_, LiH, MgB_4_, B and H_2_.

## Abstract 2

The crystal structure of the new tetra­gonal boride Mg_*x*_Li_3 - *x*_B_48 - *y*_ (*x* = 1.11, *x* = 0.40) has been determined by the single-crystal method. This new structure type is closely related to the structural family comprising tetra­gonal β-boron, α-AlB_12_ and Be_0.7_Al_1.1_B_22_.

## 1. Chemical context

The main requirements for modern hydrogen-storage mat­erials in automotive applications are high gravimetric uptake (above 6.5 wt.% capacity for hydrogen), absorption/desorp­tion of hydrogen at moderate temperatures and pressures, as well as a relatively low cost of the materials and their environmental safety (Sakintuna *et al.*, 2007 ▸). Conventional metal compositions, such as LaNi_5_ and its substitutional derivatives, titanium and zirconium alloys that are commonly used as hydrogen-storage systems, have a capacity of nearly 1.5 wt.% and thus they cannot satisfy the current needs. More promising candidates for the development of light-weight hydrogen-storage materials may be found with new formulations based on light metals such as Mg and Li (Herbst & Meyer, 2010 ▸; Pavlyuk *et al.*, 2013 ▸; Pavlyuk *et al.*, 2018 ▸, 2019*a* ▸,*b* ▸). Upon hydrogenation with boron, magnesium and lithium form complex hydrides of particularly high capacity. Two paradigmatic compositions of Mg(BH_4_)_2_ and LiBH_4_ contain as much as 14.9 and 18.5 wt.% of hydrogen, respectively (Li *et al.*, 2023 ▸; Puszkiel *et al.*, 2019 ▸). Therefore, new insights into the solid-state chemistry of Mg–Li–B ternary alloys and their hydrogen-sorption properties are of primary importance. While studying boron-rich alloys, we have succeeded in the preparation of a new ternary phase of the approximate composition Mg_x_Li_3  − *x*_B_48 − *y*_ (*x* = 1.11, *y* = 0.40) and we report its structure here.

## 2. Structural commentary

**Figure 1:**
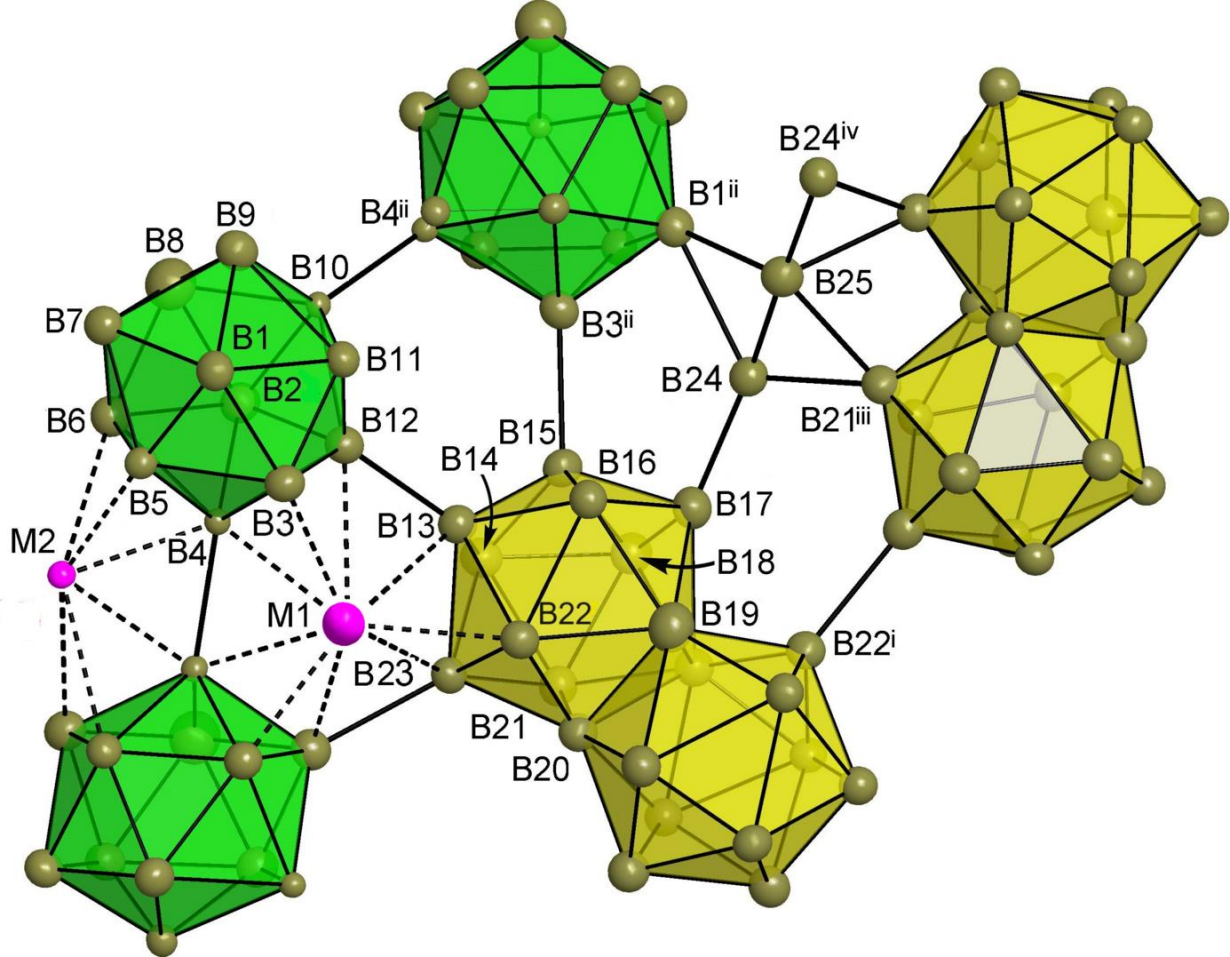
Structure of Mg_*x*_Li_3 − *x*_Mg_48 − *y*_ showing the atom-labelling scheme and displacement ellipsoids drawn at the 30% probability level. Two kinds of boride polyhedra are indicated – green [B_12_] icosa­hedra and yellow fused [B_21_] double icosa­hedra – and the two mixed Li/Mg sites *M*1 and *M*2. [Symmetry codes: (i) −*y* + 1, −*x* + 1, −*z* + 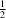 ; (ii) −*y* + 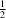 , *x* + 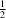 , *z* − 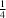 ; (iii) *x* + 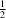 , −*y* + 1.5, −*z* + 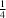 ; (iv) *y*, *x*, −*z*.]

**Figure 2:**
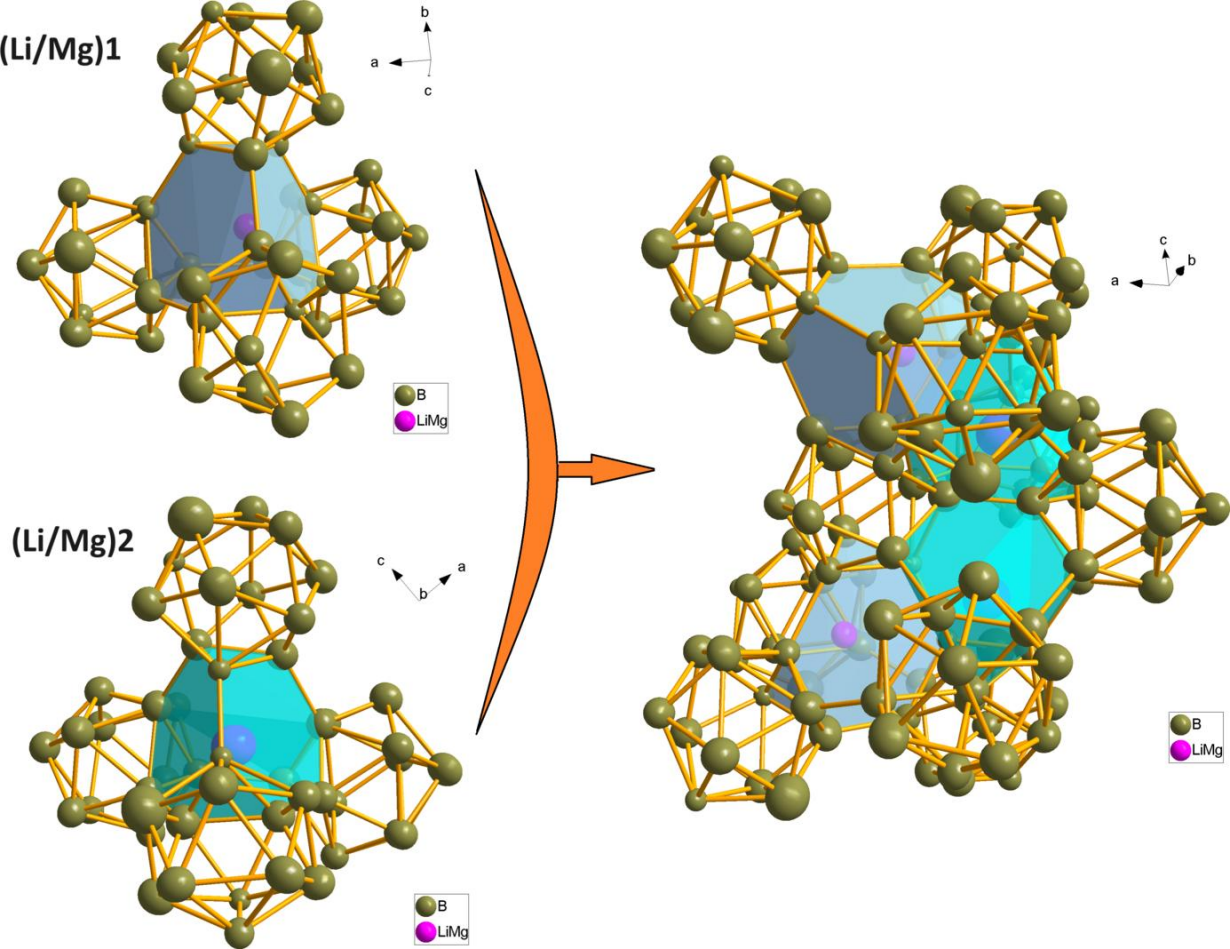
The connection of [*M*1B_12_] and [*M*2B_12_] truncated tetra­hedra with [B_12_] icosa­hedra.

**Figure 3:**
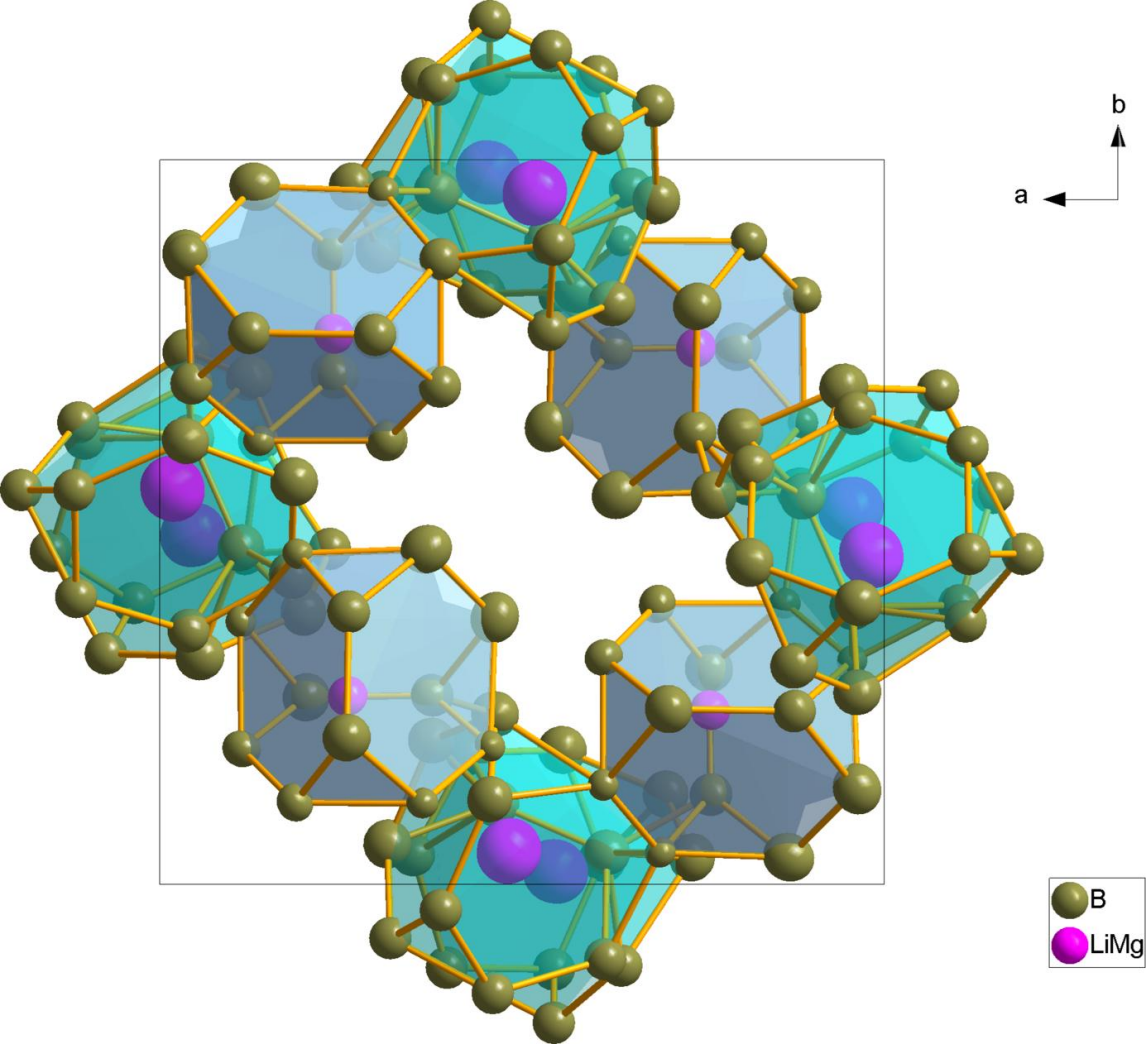
Packing of [*M*1B_12_] (green) and [*M*2B_12_] (grey) truncated tetra­hedra in the unit cell.

In the title structure, a statistical mixture of Mg/Li atoms occupies one 8*b* site with relative partial occupancies Mg:Li = 0.495 (9):0.505 (9) and one 4*a* site with Mg:Li = 0.097 (8):0.903 (8). The boron atoms occupy 23 8*b* sites (symmetry 1) and two 4*a* sites (symmetry ..2). Fig. 1 ▸ shows the two mixed Mg/Li sites (labelled as *M*1 and *M*2 for Mg1/Li1 and Mg2/Li2, respectively) and two kinds of the distinct boride polyhedra, which are [B_12_] icosa­hedra and fused [B_21_] double icosa­hedra. The latter are situated across twofold axes passing through the B19 atom. The twelvefold coordination environments in the form of truncated tetra­hedra (Laves polyhedra) are characteristic for both kinds of Mg/Li statistical mixtures. Every such [*M*2B_12_] truncated tetra­hedron is connected by triangular faces to four [B_12_] icosa­hedra, whereas the connection of [*M*1B_12_] comprises face-sharing with two [B_12_] icosa­hedra and two fused [B_21_] double icosa­hedra. The arrangement of the truncated tetra­hedra in the environment of the boride clusters is shown in Fig. 2 ▸. The [*M*1B_12_] and [*M*2B_12_] truncated tetra­hedra themselves are mutually connected by edges (Fig. 3 ▸).

The boron atoms exhibit four kinds of polyhedra, *viz*. penta­gonal pyramid (coordination number CN = 6), distorted tetra­gonal pyramid (CN = 5), bicapped hexa­gon (CN = 8) and gyrobifastigium (CN = 8). Analysis of the inter­atomic separations suggests that the shortest B—B bonds of 1.587 (5)–1.642 (6) Å are close to the same distances in the crystal structure of elemental boron (1.624 Å) and known binary borides (1.601–1.690 Å; Vlasse *et al.*, 1978 ▸, 1979 ▸; Kasper *et al.*, 1977 ▸). The shortest distances of boron to the Mg1/Li1 and Mg2/Li2 atoms are 2.067 (7) and 1.823 (6) Å, respectively. A slight shortening of the latter distance is obviously related to the partial ionisation of Mg/Li atoms.

## 3. Synthesis and crystallization

The Mg–Li–B samples were prepared from the following reactants: magnesium (powder, ≥99%) lithium (rod, 99.9%) and boron (powder, 99.99%). Appropriate amounts of the components were mixed and pressed into a tablet at a pressure of 6 bar. The tablet was closed inside a tantalum crucible in a glove-box under an argon atmosphere. The crucible was sealed by arc melting under a dry argon atmosphere. The reaction between the components was initiated in an induction furnace at 1173 K. After 10 min, the sample was cooled to 670 K and homogenized over 48 h. A prismatic-like single-crystal of the title compound, metallic dark grey in colour, was isolated by mechanical fragmentation from the alloy, the starting composition of which was Mg_5_Li_5_B_90_ (in at. %). The structure refinements give composition Mg_1.11_Li_1.89_B_47.60_ (MgLi_2_B_48_) or in at. % as Mg_2.22_Li_3.78_B_95.2_.

**Figure 4:**
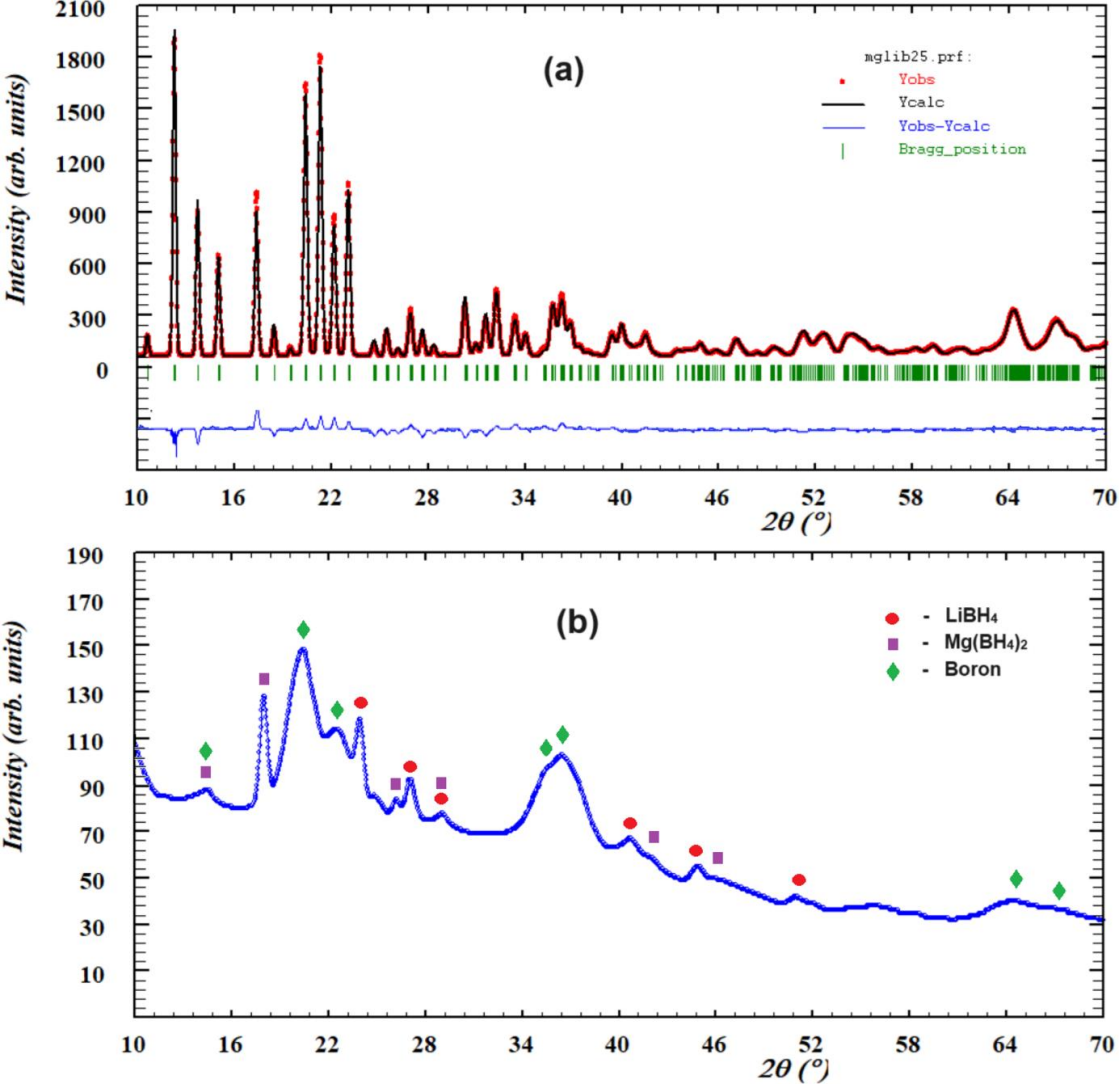
The observed powder X-ray diffraction patterns (Cu *K*α radiation) for the sample MgLi_2_B_48_ before hydrogenation and after gas-phase hydrogenation.

The bulk sample was also examined by X-ray powder diffraction (diffractometer Rigaku Miniflex D-600, Cu *K*α radiation). X-ray phase analysis indicates that the prepared sample is a single phase (Fig. 4 ▸ *a*).

Taking into account the fact that the compound consists of elements prone to hydride formation, we have examined its hydrogen-sorption properties. Gas-phase hydrogenation was performed using the IMI-COR Hiden Isochema apparatus in the temperature range from 293 to 773 K and hydrogen pressure up to 200 bar for an alloy with composition MgLi_2_B_48_. The hydrogenation results in the formation of LiBH_4_–Mg(BH_4_)_2_ hydride composite and amorphous boron:

MgLi_2_B_48_ + 8H_2_ = 2LiBH_4_ + Mg(BH_4_)_2_ + 44B_(amorph)_

According to the experimental data on phase diagram of the LiBH_4_–Mg(BH_4_)_2_ system (Bardají *et al.*, 2011 ▸), MgLi(BH_4_)_3_ is a eutectic composite [LiBH_4_+Mg(BH_4_)_2_] melting at 453 K. The decomposition temperature of the eutectics is lower than those of the individual LiBH_4_ and Mg(BH_4_)_2_. In the temperature range 473 to 643 K, the composite releases about 9.0 wt % of hydrogen (Nale *et al.*, 2011 ▸; Bardají *et al.*, 2011 ▸). Decomposition of the LiBH_4_–Mg(BH_4_)_2_ eutectics, in the temperature range 543 to 623 K, results in the formation of the following products:

2(LiBH_4_–Mg(BH_4_)_2_) = MgH_2_ + 2LiH + MgB_4_ + 2B + 10H_2_

## 4. Database survey

The title compound crystallizes in a new structure type, which is closely related to the structural family comprising tetra­gonal β-boron (Vlasse *et al.*, 1978 ▸, 1979 ▸), α-AlB_12_ (Kasper *et al.*, 1977 ▸) and Be_0.7_Al_1.1_B_22_ types (Higashi, 1980 ▸). Tetra­gonal β-boron crystallizes in the tetra­gonal crystal system with space group *P*4_1_ (*a* = 10.14 and *c* = 14.17 Å). Boron atoms occupy 49 positions of Wykoff 4*a*, three of which are half-populated. Boride α-AlB_12_ crystallizes in the tetra­gonal crystal system with space group *P*4_1_2_1_2 (*a* = 10.158 and *c* =14.270 Å). The aluminium atoms partially occupy five 8*b* sites. The boron atoms occupy 21 8*b* sites and two 4*a* sites. The Be_0.7_Al_1.1_B_22_ compound (*P*4_1_2_1_2, *a* = 10.168 and *c* = 14.262 Å) crystallizes in the same crystal system and space group as AlB_12_ but it adopts a different site distribution of the atoms. Boron atoms occupy 21 8*b* sites and two 4*a* sites. Aluminium atoms partially occupy three 8*b* sites and the beryllium atoms partially occupy five 8*b* sites.

**Figure 5:**
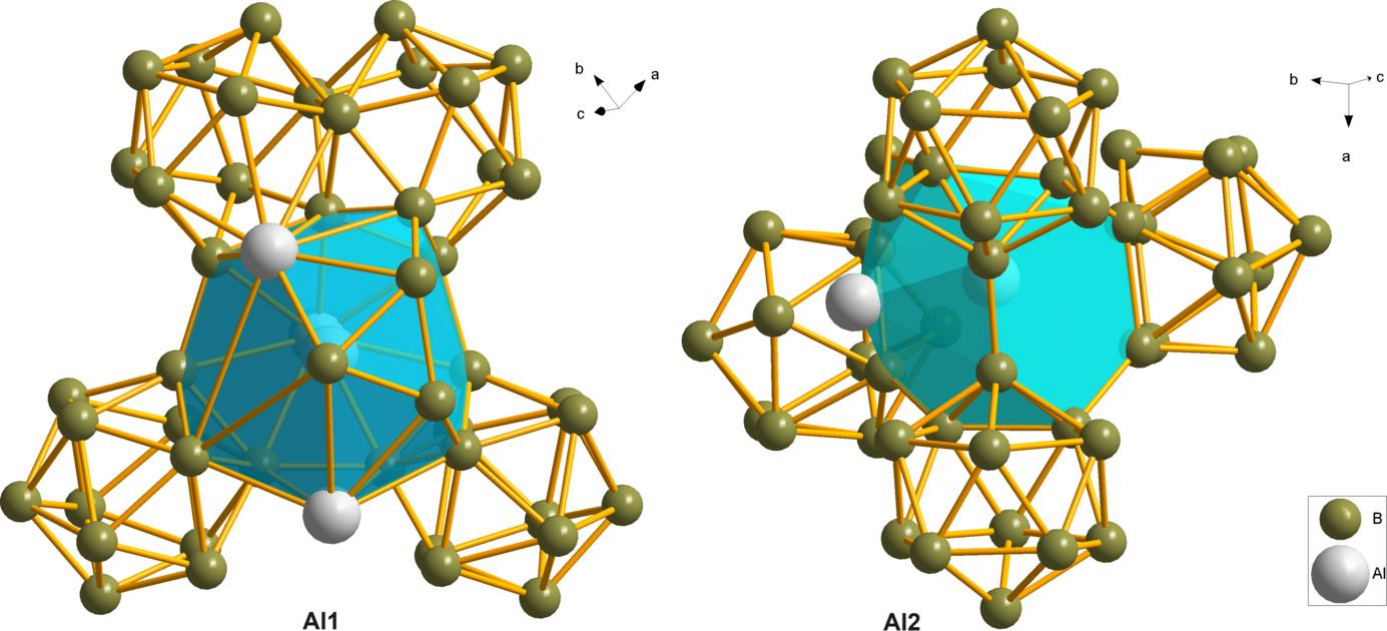
The connection of two types of Al truncated tetra­hedra with [B_12_] icosa­hedra in the structure of Al_2 − *x*_B_22_ (Kasper *et al.*, 1977 ▸).

Al_2 − *x*_B_22_ or α-AlB_12_ (Kasper *et al.*, 1977 ▸) and Mg_5 − *x*_B_44_ (Pediaditakis *et al.*, 2010 ▸) crystallize in the enanti­omorphic space groups *P*4_1_2_1_2 and *P*4_3_2_1_2, while adopting different structure types than the title compound. In these cases, all five 8*b* sites are partially occupied by the Al or Mg atoms and three of them are split. In addition, the coordination polyhedra of Al and Mg atoms are different (CN = 16 and CN = 12) as may be compared to the title structure. In addition to the boron atoms, these coordination environments also include metal atoms, which is a direct consequence of a more metal-rich composition. For example, Fig. 5 ▸ shows how the [B_12_] icosa­hedra are connected with the selected aluminium polyhedra and this mode differs from the one observed for the [B_12_] icosa­hedra and truncated tetra­hedra in the title phase.

## 5. Refinement

The structure was solved by direct methods after an empirical absorption correction. In the first stage of the refinement, the positions of the boron and Mg/Li atoms were obtained correctly by direct methods. The B19 atom (Wyckoff site 4*a*) showed displacement parameters considerably different than those of other boron atom sites, suggesting that this position is partially occupied and the subsequent refinement led to the occupancy factor of 0.61 (2). All atoms were freely refined anisotropically. Crystal data, data collection and structure refinement details are summarized in Table 1 ▸.

## Supplementary Material

Crystal structure: contains datablock(s) I. DOI: 10.1107/S2056989023009969/nu2001sup1.cif

Structure factors: contains datablock(s) I. DOI: 10.1107/S2056989023009969/nu2001Isup2.hkl

CCDC reference: 2308501

Additional supporting information:  crystallographic information; 3D view; checkCIF report

## Figures and Tables

**Table 1:** Experimental details

Crystal data	
Chemical formula	Mg_1.11_Li_1.89_B_47.60_
*M*_r_	554.61
Crystal system, space group	Tetragonal, *P*4_3_2_1_2
Temperature (K)	296
*a*, *c* (Å)	10.1905 (6), 14.4709 (10)
*V* (Å^3^)	1502.7 (2)
*Z*	4
Radiation type	Mo *K*α
μ (mm^−1^)	0.13
Crystal size (mm)	0.08 × 0.06 × 0.04

Data collection	
Diffractometer	Oxford Diffraction Xcalibur3 CCD
Absorption correction	Analytical (*CrysAlis RED*; Oxford Diffraction, 2008 ▸)
*T*_min_, *T*_max_	0.932, 0.994
No. of measured, independent and observed [*I* > 2σ(*I*)] reflections	3441, 1721, 1506
*R*_int_	0.042
(sin θ/λ)_max_ (Å^−1^)	0.648

Refinement	
*R*[*F* ^2^ > 2σ(*F* ^2^)], *wR*(*F* ^2^), *S*	0.053, 0.129, 1.00
No. of reflections	1721
No. of parameters	227
Δρ_max_, Δρ_min_ (e Å^−3^)	0.28, −0.29

Computer programs: *CrysAlis CCD* and *CrysAlis RED* (Oxford Diffraction, 2008 ▸), *SHELXS97* (Sheldrick, 2008 ▸), *SHELXL2014/7* (Sheldrick, 2015 ▸) and *DIAMOND* (Brandenburg, 2006 ▸).

## supplementary crystallographic information

### Crystal data

**Table:**

Mg_1.11_Li_1.89_B_47.60_	*D*_x_ = 2.451 Mg m^−^^3^
*M**_r_* = 554.61	Mo *K*α radiation, λ = 0.71073 Å
Tetragonal, *P*4_3_2_1_2	Cell parameters from 1506 reflections
*a* = 10.1905 (6) Å	θ = 2.5–27.4°
*c* = 14.4709 (10) Å	µ = 0.13 mm^−^^1^
*V* = 1502.7 (2) Å^3^	*T* = 296 K
*Z* = 4	Prism, dark grey
*F*(000) = 1028	0.08 × 0.06 × 0.04 mm

### Data collection

**Table:**

Oxford Diffraction Xcalibur3 CCD diffractometer	1721 independent reflections
Radiation source: fine-focus sealed tube	1506 reflections with *I* > 2σ(*I*)
Graphite monochromator	*R*_int_ = 0.042
ω scans	θ_max_ = 27.4°, θ_min_ = 2.4°
Absorption correction: analytical (CrysAlis RED; Oxford Diffraction, 2008)	*h* = −13→13
*T*_min_ = 0.932, *T*_max_ = 0.994	*k* = −9→9
3441 measured reflections	*l* = −18→18

### Refinement

**Table:**

Refinement on *F*^2^	0 restraints
Least-squares matrix: full	Primary atom site location: structure-invariant direct methods
*R*[*F*^2^ > 2σ(*F*^2^)] = 0.053	Secondary atom site location: difference Fourier map
*wR*(*F*^2^) = 0.129	*w* = 1/[σ^2^(*F*_o_^2^) + (0.0462*P*)^2^] where *P* = (*F*_o_^2^ + 2*F*_c_^2^)/3
*S* = 1.00	(Δ/σ)_max_ < 0.001
1721 reflections	Δρ_max_ = 0.28 e Å^−^^3^
227 parameters	Δρ_min_ = −0.29 e Å^−^^3^

### Special details

**Table:**

Geometry. All esds (except the esd in the dihedral angle between two l.s. planes) are estimated using the full covariance matrix. The cell esds are taken into account individually in the estimation of esds in distances, angles and torsion angles; correlations between esds in cell parameters are only used when they are defined by crystal symmetry. An approximate (isotropic) treatment of cell esds is used for estimating esds involving l.s. planes.

### Fractional atomic coordinates and isotropic or equivalent isotropic displacement parameters (Å^2^)

**Table:**

	*x*	*y*	*z*	*U*_iso_*/*U*_eq_	Occ. (<1)
B1	0.0296 (5)	0.1075 (5)	0.1722 (3)	0.0450 (10)	
B2	−0.1031 (5)	0.3851 (5)	0.0553 (3)	0.0479 (10)	
B3	0.0373 (5)	0.2648 (5)	0.2183 (3)	0.0462 (10)	
B4	−0.1125 (3)	0.3627 (3)	0.1818 (2)	0.0204 (6)	
B5	−0.1135 (4)	0.1867 (4)	0.1993 (2)	0.0300 (7)	
B6	−0.2025 (5)	0.2581 (5)	0.1026 (3)	0.0510 (11)	
B7	−0.1212 (5)	0.0989 (4)	0.0982 (3)	0.0451 (10)	
B8	−0.1069 (7)	0.2213 (7)	0.0065 (5)	0.0796 (19)	
B9	0.0373 (5)	0.1313 (5)	0.0427 (3)	0.0503 (11)	
B10	0.0440 (3)	0.3090 (3)	0.0217 (2)	0.0195 (6)	
B11	0.1235 (4)	0.2397 (4)	0.1241 (3)	0.0377 (8)	
B12	0.0442 (5)	0.3828 (5)	0.1306 (3)	0.0442 (10)	
B13	0.1120 (5)	0.5355 (5)	0.1575 (3)	0.0451 (10)	
B14	0.0552 (5)	0.6852 (5)	0.1140 (3)	0.0498 (11)	
B15	0.1983 (5)	0.6239 (5)	0.0812 (3)	0.0448 (10)	
B16	0.2913 (4)	0.5334 (5)	0.1726 (3)	0.0438 (9)	
B17	0.3319 (5)	0.7003 (5)	0.1173 (3)	0.0501 (11)	
B18	0.1937 (5)	0.7942 (5)	0.0920 (3)	0.0516 (11)	
B19	0.3318 (9)	0.6682 (9)	0.2500	0.056 (4)	0.61 (2)
B20	0.1714 (4)	0.7328 (5)	0.2927 (3)	0.0449 (10)	
B21	0.0910 (5)	0.8131 (5)	0.1950 (3)	0.0452 (10)	
B22	0.1816 (4)	0.5744 (5)	0.2717 (3)	0.0445 (10)	
B23	0.0402 (4)	0.6501 (4)	0.2442 (3)	0.0380 (8)	
B24	0.4342 (5)	0.6457 (5)	0.0306 (3)	0.0456 (10)	
B25	0.5384 (5)	0.5384 (5)	0.0000	0.0494 (15)	
Mg1	0.0173 (5)	0.4554 (4)	0.2722 (3)	0.113 (2)*	0.495 (9)
Li1	0.0173 (5)	0.4554 (4)	0.2722 (3)	0.113 (2)*	0.505 (9)
Mg2	−0.2597 (3)	0.2597 (3)	0.2500	0.0211 (15)*	0.097 (8)
Li2	−0.2597 (3)	0.2597 (3)	0.2500	0.0211 (15)*	0.903 (8)

### Atomic displacement parameters (Å^2^)

**Table:**

	*U*^11^	*U*^22^	*U*^33^	*U*^12^	*U*^13^	*U*^23^
B1	0.043 (2)	0.041 (2)	0.051 (2)	−0.0033 (19)	0.0019 (19)	0.0023 (18)
B2	0.050 (3)	0.047 (2)	0.048 (2)	−0.001 (2)	−0.0011 (19)	−0.0001 (19)
B3	0.048 (2)	0.044 (2)	0.047 (2)	0.000 (2)	0.001 (2)	−0.0033 (19)
B4	0.0204 (14)	0.0211 (14)	0.0196 (12)	−0.0005 (12)	−0.0004 (10)	0.0009 (11)
B5	0.0315 (17)	0.0286 (17)	0.0298 (15)	0.0015 (14)	−0.0024 (14)	0.0000 (13)
B6	0.047 (2)	0.050 (3)	0.056 (3)	−0.001 (2)	0.003 (2)	−0.001 (2)
B7	0.051 (3)	0.045 (2)	0.039 (2)	−0.003 (2)	0.0030 (18)	0.0008 (18)
B8	0.076 (4)	0.077 (4)	0.086 (4)	0.002 (3)	0.001 (4)	−0.003 (4)
B9	0.049 (3)	0.056 (3)	0.046 (2)	0.002 (2)	0.001 (2)	0.001 (2)
B10	0.0184 (13)	0.0212 (14)	0.0190 (12)	−0.0009 (11)	−0.0001 (11)	−0.0005 (11)
B11	0.036 (2)	0.038 (2)	0.0390 (19)	−0.0010 (16)	0.0009 (16)	0.0005 (15)
B12	0.043 (2)	0.045 (2)	0.045 (2)	0.000 (2)	0.0021 (18)	0.0015 (19)
B13	0.045 (2)	0.047 (2)	0.044 (2)	0.000 (2)	−0.0002 (19)	−0.0007 (19)
B14	0.053 (3)	0.048 (2)	0.049 (2)	0.000 (2)	0.003 (2)	−0.001 (2)
B15	0.042 (2)	0.046 (2)	0.046 (2)	−0.0005 (19)	−0.0043 (18)	−0.0017 (19)
B16	0.043 (2)	0.044 (2)	0.044 (2)	−0.0012 (18)	0.0018 (18)	0.0017 (18)
B17	0.049 (3)	0.048 (3)	0.053 (3)	−0.001 (2)	0.002 (2)	0.003 (2)
B18	0.052 (3)	0.051 (3)	0.052 (3)	0.001 (2)	0.001 (2)	−0.002 (2)
B19	0.054 (5)	0.054 (5)	0.060 (7)	−0.007 (5)	−0.001 (4)	−0.001 (4)
B20	0.044 (2)	0.044 (2)	0.046 (2)	0.0007 (18)	−0.0009 (18)	0.0008 (18)
B21	0.044 (2)	0.048 (2)	0.043 (2)	−0.0021 (19)	−0.0030 (19)	0.0009 (19)
B22	0.042 (2)	0.045 (2)	0.047 (2)	0.0026 (17)	0.0023 (18)	0.0003 (19)
B23	0.036 (2)	0.040 (2)	0.0376 (19)	0.0000 (16)	0.0003 (16)	0.0020 (17)
B24	0.048 (2)	0.045 (2)	0.044 (2)	−0.004 (2)	−0.0014 (19)	−0.0001 (19)
B25	0.052 (2)	0.052 (2)	0.044 (3)	0.002 (3)	0.000 (2)	0.000 (2)

### Geometric parameters (Å, º)

**Table:**

B1—B5	1.712 (6)	B14—B21	1.790 (7)
B1—B3	1.739 (7)	B14—B18	1.824 (7)
B1—B11	1.793 (6)	B14—B22^ii^	1.879 (7)
B1—B25^i^	1.867 (6)	B14—B23	1.923 (6)
B1—B7	1.875 (7)	B14—Li1^ii^	2.169 (7)
B1—B9	1.892 (6)	B14—Mg1^ii^	2.169 (7)
B1—B24^i^	2.010 (7)	B15—B17	1.652 (7)
B2—B10	1.756 (6)	B15—B18	1.743 (7)
B2—B6	1.780 (7)	B15—B16	1.870 (6)
B2—B8	1.813 (8)	B15—B3^ii^	1.894 (7)
B2—B4	1.847 (5)	B15—Li1^ii^	2.089 (6)
B2—B12	1.855 (7)	B15—Mg1^ii^	2.089 (6)
B2—B23^ii^	1.873 (6)	B16—B7^vii^	1.643 (7)
B2—Li1^ii^	2.076 (7)	B16—B19	1.821 (7)
B2—Mg1^ii^	2.076 (7)	B16—B22	1.866 (6)
B3—B11	1.642 (6)	B16—B17	1.925 (7)
B3—B12	1.749 (6)	B17—B24	1.724 (7)
B3—B5	1.752 (6)	B17—B18	1.741 (8)
B3—B15^i^	1.894 (7)	B17—B19	1.948 (5)
B3—B4	1.899 (6)	B17—B20^viii^	1.961 (7)
B3—Mg1	2.103 (6)	B18—B8^ix^	1.768 (9)
B4—B12	1.771 (5)	B18—B21	1.831 (7)
B4—B10^i^	1.799 (4)	B18—B20^viii^	1.862 (7)
B4—B5	1.811 (5)	B19—B16^viii^	1.820 (7)
B4—B6	1.813 (6)	B19—B22	1.832 (5)
B4—Mg2	2.080 (3)	B19—B22^viii^	1.832 (5)
B4—Mg1	2.087 (6)	B19—B20^viii^	1.868 (12)
B5—B7	1.717 (6)	B19—B20	1.868 (12)
B5—B5^iii^	1.807 (7)	B19—B17^viii^	1.948 (5)
B5—B6	1.820 (6)	B20—B22	1.646 (6)
B5—Mg2	1.820 (6)	B20—B23	1.729 (6)
B6—B8	1.739 (9)	B20—B21^viii^	1.811 (7)
B6—B11^iv^	1.805 (6)	B20—B21	1.827 (6)
B6—B7	1.823 (7)	B20—B20^viii^	1.852 (9)
B6—Mg2	2.212 (5)	B20—B18^viii^	1.862 (7)
B7—B16^iv^	1.643 (7)	B20—B17^viii^	1.961 (7)
B7—B8	1.827 (8)	B21—B24^x^	1.689 (7)
B7—B9	1.835 (7)	B21—B25^xi^	1.792 (7)
B8—B18^v^	1.768 (9)	B21—B20^viii^	1.811 (7)
B8—B10	1.793 (7)	B21—B23	1.880 (6)
B8—B9	1.810 (9)	B21—B21^viii^	2.108 (9)
B9—B9^vi^	1.833 (10)	B22—B23	1.682 (6)
B9—B10	1.838 (6)	B22—B14^i^	1.879 (7)
B9—B11	1.838 (7)	B22—Mg1	2.067 (7)
B9—Li2^ii^	2.427 (7)	B23—B2^i^	1.873 (6)
B9—Mg2^ii^	2.427 (7)	B23—Mg1	2.038 (6)
B10—B12	1.746 (5)	B24—B25	1.587 (5)
B10—B4^ii^	1.799 (4)	B24—B21^xii^	1.689 (7)
B10—B11	1.831 (5)	B24—B1^ii^	2.010 (7)
B10—Li1^ii^	2.123 (6)	B25—B24^vi^	1.587 (5)
B10—Mg1^ii^	2.123 (6)	B25—B21^xii^	1.792 (7)
B10—Li2^ii^	2.142 (4)	B25—B21^xiii^	1.792 (7)
B10—Mg2^ii^	2.142 (4)	B25—B1^ii^	1.867 (6)
B11—B12	1.670 (6)	B25—B1^vii^	1.867 (6)
B11—B6^vii^	1.805 (6)	Mg1—B2^i^	2.076 (7)
B11—Li2^ii^	2.154 (5)	Mg1—B13^i^	2.083 (6)
B11—Mg2^ii^	2.154 (5)	Mg1—B12^i^	2.083 (6)
B12—B13	1.747 (6)	Mg1—B15^i^	2.089 (6)
B12—Li1^ii^	2.083 (6)	Mg1—B10^i^	2.123 (6)
B12—Mg1^ii^	2.083 (6)	Mg1—B14^i^	2.169 (7)
B12—Mg1	2.196 (6)	Mg2—B5^iii^	1.820 (6)
B13—B15	1.675 (7)	Mg2—B4^iii^	2.081 (3)
B13—B14	1.749 (7)	Mg2—B10^iv^	2.142 (4)
B13—B16	1.840 (7)	Mg2—B10^i^	2.142 (4)
B13—B22	1.842 (6)	Mg2—B11^iv^	2.154 (5)
B13—B23	1.863 (6)	Mg2—B11^i^	2.154 (5)
B13—Li1^ii^	2.083 (6)	Mg2—B6^iii^	2.212 (5)
B13—Mg1^ii^	2.083 (6)	Mg2—B9^iv^	2.427 (7)
B13—Mg1	2.086 (6)	Mg2—B9^i^	2.427 (7)
B14—B15	1.656 (7)		
			
B5—B1—B3	61.0 (3)	B22^ii^—B14—Li1^ii^	60.9 (2)
B5—B1—B11	100.9 (3)	B23—B14—Li1^ii^	116.7 (3)
B3—B1—B11	55.4 (2)	B15—B14—Mg1^ii^	64.6 (3)
B5—B1—B25^i^	106.2 (3)	B13—B14—Mg1^ii^	63.2 (2)
B3—B1—B25^i^	120.0 (3)	B21—B14—Mg1^ii^	170.2 (4)
B11—B1—B25^i^	143.8 (3)	B18—B14—Mg1^ii^	114.3 (3)
B5—B1—B7	57.0 (2)	B22^ii^—B14—Mg1^ii^	60.9 (2)
B3—B1—B7	107.4 (3)	B23—B14—Mg1^ii^	116.7 (3)
B11—B1—B7	104.5 (3)	Li1^ii^—B14—Mg1^ii^	0.0
B25^i^—B1—B7	110.3 (3)	B17—B15—B14	117.2 (4)
B5—B1—B9	101.7 (3)	B17—B15—B13	118.5 (4)
B3—B1—B9	105.1 (3)	B14—B15—B13	63.3 (3)
B11—B1—B9	59.8 (3)	B17—B15—B18	61.6 (3)
B25^i^—B1—B9	134.2 (3)	B14—B15—B18	64.8 (3)
B7—B1—B9	58.3 (2)	B13—B15—B18	117.5 (4)
B5—B1—B24^i^	115.0 (3)	B17—B15—B16	65.9 (3)
B3—B1—B24^i^	82.5 (3)	B14—B15—B16	115.4 (3)
B11—B1—B24^i^	98.5 (3)	B13—B15—B16	62.3 (3)
B25^i^—B1—B24^i^	48.15 (19)	B18—B15—B16	116.2 (3)
B7—B1—B24^i^	156.7 (3)	B17—B15—B3^ii^	109.2 (3)
B9—B1—B24^i^	140.8 (3)	B14—B15—B3^ii^	126.7 (3)
B10—B2—B6	105.7 (3)	B13—B15—B3^ii^	115.0 (3)
B10—B2—B8	60.3 (3)	B18—B15—B3^ii^	123.1 (3)
B6—B2—B8	57.9 (3)	B16—B15—B3^ii^	106.2 (3)
B10—B2—B4	105.3 (3)	B17—B15—Li1^ii^	172.7 (4)
B6—B2—B4	60.0 (2)	B14—B15—Li1^ii^	69.7 (3)
B8—B2—B4	105.7 (3)	B13—B15—Li1^ii^	66.1 (3)
B10—B2—B12	57.7 (2)	B18—B15—Li1^ii^	122.3 (4)
B6—B2—B12	103.0 (3)	B16—B15—Li1^ii^	114.4 (3)
B8—B2—B12	103.6 (4)	B3^ii^—B15—Li1^ii^	63.5 (2)
B4—B2—B12	57.2 (2)	B17—B15—Mg1^ii^	172.7 (4)
B10—B2—B23^ii^	117.4 (3)	B14—B15—Mg1^ii^	69.7 (3)
B6—B2—B23^ii^	130.5 (4)	B13—B15—Mg1^ii^	66.1 (3)
B8—B2—B23^ii^	126.0 (4)	B18—B15—Mg1^ii^	122.3 (4)
B4—B2—B23^ii^	124.0 (3)	B16—B15—Mg1^ii^	114.4 (3)
B12—B2—B23^ii^	119.7 (3)	B3^ii^—B15—Mg1^ii^	63.5 (2)
B10—B2—Li1^ii^	66.7 (2)	Li1^ii^—B15—Mg1^ii^	0.0
B6—B2—Li1^ii^	166.6 (4)	B7^vii^—B16—B19	127.5 (5)
B8—B2—Li1^ii^	121.6 (4)	B7^vii^—B16—B13	121.8 (4)
B4—B2—Li1^ii^	110.2 (3)	B19—B16—B13	106.8 (5)
B12—B2—Li1^ii^	63.7 (2)	B7^vii^—B16—B22	130.5 (3)
B23^ii^—B2—Li1^ii^	61.9 (2)	B19—B16—B22	59.6 (3)
B10—B2—Mg1^ii^	66.7 (2)	B13—B16—B22	59.6 (2)
B6—B2—Mg1^ii^	166.6 (4)	B7^vii^—B16—B15	123.4 (3)
B8—B2—Mg1^ii^	121.6 (4)	B19—B16—B15	100.2 (4)
B4—B2—Mg1^ii^	110.2 (3)	B13—B16—B15	53.7 (2)
B12—B2—Mg1^ii^	63.7 (2)	B22—B16—B15	97.4 (3)
B23^ii^—B2—Mg1^ii^	61.9 (2)	B7^vii^—B16—B17	122.2 (3)
Li1^ii^—B2—Mg1^ii^	0.0	B19—B16—B17	62.6 (3)
B11—B3—B1	64.0 (3)	B13—B16—B17	98.8 (3)
B11—B3—B12	58.9 (3)	B22—B16—B17	104.5 (3)
B1—B3—B12	110.9 (3)	B15—B16—B17	51.6 (2)
B11—B3—B5	105.5 (3)	B15—B17—B24	96.7 (3)
B1—B3—B5	58.7 (2)	B15—B17—B18	61.8 (3)
B12—B3—B5	103.5 (3)	B24—B17—B18	120.9 (4)
B11—B3—B15^i^	119.9 (3)	B15—B17—B16	62.5 (3)
B1—B3—B15^i^	122.3 (3)	B24—B17—B16	98.5 (3)
B12—B3—B15^i^	118.1 (3)	B18—B17—B16	113.5 (3)
B5—B3—B15^i^	129.3 (3)	B15—B17—B19	103.4 (3)
B11—B3—B4	106.3 (3)	B24—B17—B19	131.6 (5)
B1—B3—B4	109.9 (3)	B18—B17—B19	107.4 (5)
B12—B3—B4	57.9 (2)	B16—B17—B19	56.1 (3)
B5—B3—B4	59.3 (2)	B15—B17—B20^viii^	104.3 (3)
B15^i^—B3—B4	120.7 (3)	B24—B17—B20^viii^	154.5 (4)
B11—B3—Mg1	120.3 (3)	B18—B17—B20^viii^	60.1 (3)
B1—B3—Mg1	171.8 (4)	B16—B17—B20^viii^	103.9 (3)
B12—B3—Mg1	68.8 (3)	B19—B17—B20^viii^	57.1 (4)
B5—B3—Mg1	113.1 (3)	B17—B18—B15	56.6 (3)
B15^i^—B3—Mg1	62.8 (2)	B17—B18—B8^ix^	110.7 (4)
B4—B3—Mg1	62.6 (2)	B15—B18—B8^ix^	119.4 (4)
B12—B4—B10^i^	129.9 (3)	B17—B18—B14	104.7 (4)
B12—B4—B5	100.2 (3)	B15—B18—B14	55.3 (3)
B10^i^—B4—B5	114.8 (2)	B8^ix^—B18—B14	127.6 (4)
B12—B4—B6	105.0 (3)	B17—B18—B21	110.5 (3)
B10^i^—B4—B6	122.9 (2)	B15—B18—B21	101.2 (3)
B5—B4—B6	60.3 (2)	B8^ix^—B18—B21	133.4 (4)
B12—B4—B2	61.6 (2)	B14—B18—B21	58.7 (3)
B10^i^—B4—B2	132.4 (3)	B17—B18—B20^viii^	65.8 (3)
B5—B4—B2	105.2 (2)	B15—B18—B20^viii^	104.9 (3)
B6—B4—B2	58.2 (2)	B8^ix^—B18—B20^viii^	123.4 (4)
B12—B4—B3	56.8 (2)	B14—B18—B20^viii^	105.6 (3)
B10^i^—B4—B3	115.7 (2)	B21—B18—B20^viii^	58.7 (3)
B5—B4—B3	56.3 (2)	B16^viii^—B19—B16	136.2 (8)
B6—B4—B3	105.9 (3)	B16^viii^—B19—B22	130.1 (2)
B2—B4—B3	107.4 (3)	B16—B19—B22	61.4 (2)
B12—B4—Mg2	155.0 (3)	B16^viii^—B19—B22^viii^	61.4 (2)
B10^i^—B4—Mg2	66.57 (17)	B16—B19—B22^viii^	130.1 (2)
B5—B4—Mg2	55.3 (2)	B22—B19—B22^viii^	154.4 (8)
B6—B4—Mg2	68.83 (18)	B16^viii^—B19—B20^viii^	105.7 (3)
B2—B4—Mg2	124.8 (2)	B16—B19—B20^viii^	112.2 (4)
B3—B4—Mg2	100.5 (2)	B22—B19—B20^viii^	102.6 (6)
B12—B4—Mg1	68.8 (2)	B22^viii^—B19—B20^viii^	52.8 (3)
B10^i^—B4—Mg1	65.72 (18)	B16^viii^—B19—B20	112.2 (4)
B5—B4—Mg1	111.3 (2)	B16—B19—B20	105.7 (3)
B6—B4—Mg1	169.3 (3)	B22—B19—B20	52.8 (3)
B2—B4—Mg1	122.2 (2)	B22^viii^—B19—B20	102.6 (6)
B3—B4—Mg1	63.5 (2)	B20^viii^—B19—B20	59.5 (5)
Mg2—B4—Mg1	112.81 (18)	B16^viii^—B19—B17^viii^	61.3 (2)
B1—B5—B7	66.3 (3)	B16—B19—B17^viii^	124.7 (3)
B1—B5—B3	60.2 (2)	B22—B19—B17^viii^	72.0 (2)
B7—B5—B3	114.3 (3)	B22^viii^—B19—B17^viii^	104.9 (3)
B1—B5—B5^iii^	110.05 (19)	B20^viii^—B19—B17^viii^	105.5 (5)
B7—B5—B5^iii^	117.2 (3)	B20—B19—B17^viii^	61.8 (3)
B3—B5—B5^iii^	115.0 (3)	B16^viii^—B19—B17	124.7 (3)
B1—B5—B4	115.5 (3)	B16—B19—B17	61.3 (2)
B7—B5—B4	113.4 (3)	B22—B19—B17	104.9 (3)
B3—B5—B4	64.4 (2)	B22^viii^—B19—B17	72.0 (2)
B5^iii^—B5—B4	121.7 (2)	B20^viii^—B19—B17	61.8 (3)
B1—B5—B6	115.9 (3)	B20—B19—B17	105.5 (5)
B7—B5—B6	62.0 (2)	B17^viii^—B19—B17	166.4 (8)
B3—B5—B6	112.1 (3)	B22—B20—B23	59.7 (3)
B5^iii^—B5—B6	125.7 (3)	B22—B20—B21^viii^	170.1 (4)
B4—B5—B6	59.9 (2)	B23—B20—B21^viii^	126.2 (3)
B1—B5—Mg2	169.2 (3)	B22—B20—B21	109.0 (3)
B7—B5—Mg2	121.2 (3)	B23—B20—B21	63.8 (3)
B3—B5—Mg2	118.0 (2)	B21^viii^—B20—B21	70.8 (3)
B5^iii^—B5—Mg2	60.23 (14)	B22—B20—B20^viii^	111.2 (3)
B4—B5—Mg2	69.90 (18)	B23—B20—B20^viii^	113.2 (4)
B6—B5—Mg2	74.8 (2)	B21^viii^—B20—B20^viii^	59.8 (2)
B8—B6—B2	62.0 (3)	B21—B20—B20^viii^	59.0 (2)
B8—B6—B11^iv^	134.6 (4)	B22—B20—B18^viii^	123.2 (4)
B2—B6—B11^iv^	128.5 (4)	B23—B20—B18^viii^	134.9 (4)
B8—B6—B4	110.4 (4)	B21^viii^—B20—B18^viii^	59.8 (3)
B2—B6—B4	61.9 (2)	B21—B20—B18^viii^	126.7 (3)
B11^iv^—B6—B4	111.8 (3)	B20^viii^—B20—B18^viii^	106.6 (4)
B8—B6—B5	104.5 (4)	B22—B20—B19	62.5 (3)
B2—B6—B5	107.6 (3)	B23—B20—B19	111.8 (3)
B11^iv^—B6—B5	110.5 (3)	B21^viii^—B20—B19	107.8 (3)
B4—B6—B5	59.8 (2)	B21—B20—B19	107.1 (3)
B8—B6—B7	61.7 (3)	B20^viii^—B20—B19	60.3 (2)
B2—B6—B7	112.0 (3)	B18^viii^—B20—B19	105.8 (3)
B11^iv^—B6—B7	117.6 (3)	B22—B20—B17^viii^	75.6 (3)
B4—B6—B7	108.4 (3)	B23—B20—B17^viii^	128.4 (3)
B5—B6—B7	56.3 (2)	B21^viii^—B20—B17^viii^	102.1 (3)
B8—B6—Mg2	157.0 (4)	B21—B20—B17^viii^	164.6 (4)
B2—B6—Mg2	121.1 (3)	B20^viii^—B20—B17^viii^	105.6 (3)
B11^iv^—B6—Mg2	63.9 (2)	B18^viii^—B20—B17^viii^	54.1 (3)
B4—B6—Mg2	61.30 (17)	B19—B20—B17^viii^	61.1 (2)
B5—B6—Mg2	52.6 (2)	B24^x^—B21—B14	97.2 (3)
B7—B6—Mg2	99.2 (3)	B24^x^—B21—B25^xi^	54.1 (2)
B16^iv^—B7—B5	127.5 (3)	B14—B21—B25^xi^	147.6 (4)
B16^iv^—B7—B6	119.3 (4)	B24^x^—B21—B20^viii^	153.5 (4)
B5—B7—B6	61.8 (2)	B14—B21—B20^viii^	109.3 (3)
B16^iv^—B7—B8	118.1 (4)	B25^xi^—B21—B20^viii^	100.3 (3)
B5—B7—B8	105.0 (3)	B24^x^—B21—B20	111.9 (3)
B6—B7—B8	56.9 (3)	B14—B21—B20	105.8 (3)
B16^iv^—B7—B9	123.0 (4)	B25^xi^—B21—B20	99.7 (3)
B5—B7—B9	103.8 (3)	B20^viii^—B21—B20	61.2 (3)
B6—B7—B9	104.8 (3)	B24^x^—B21—B18	137.5 (4)
B8—B7—B9	59.3 (3)	B14—B21—B18	60.5 (3)
B16^iv^—B7—B1	126.0 (3)	B25^xi^—B21—B18	128.4 (3)
B5—B7—B1	56.7 (2)	B20^viii^—B21—B18	61.5 (3)
B6—B7—B1	108.1 (3)	B20—B21—B18	109.0 (3)
B8—B7—B1	108.5 (4)	B24^x^—B21—B23	83.1 (3)
B9—B7—B1	61.3 (3)	B14—B21—B23	63.1 (3)
B6—B8—B18^v^	111.1 (5)	B25^xi^—B21—B23	119.7 (3)
B6—B8—B10	105.9 (4)	B20^viii^—B21—B23	108.2 (3)
B18^v^—B8—B10	131.9 (5)	B20—B21—B23	55.6 (2)
B6—B8—B9	109.4 (5)	B18—B21—B23	111.9 (3)
B18^v^—B8—B9	128.0 (5)	B24^x^—B21—B21^viii^	99.5 (3)
B10—B8—B9	61.3 (3)	B14—B21—B21^viii^	157.9 (2)
B6—B8—B2	60.1 (3)	B25^xi^—B21—B21^viii^	54.0 (2)
B18^v^—B8—B2	118.2 (5)	B20^viii^—B21—B21^viii^	54.9 (2)
B10—B8—B2	58.3 (3)	B20—B21—B21^viii^	54.2 (2)
B9—B8—B2	109.7 (4)	B18—B21—B21^viii^	113.5 (3)
B6—B8—B7	61.4 (3)	B23—B21—B21^viii^	104.5 (3)
B18^v^—B8—B7	115.6 (5)	B20—B22—B23	62.6 (3)
B10—B8—B7	108.6 (4)	B20—B22—B19	64.7 (4)
B9—B8—B7	60.6 (3)	B23—B22—B19	115.8 (5)
B2—B8—B7	110.3 (4)	B20—B22—B13	110.6 (3)
B8—B9—B9^vi^	119.6 (4)	B23—B22—B13	63.7 (3)
B8—B9—B7	60.2 (3)	B19—B22—B13	106.3 (3)
B9^vi^—B9—B7	131.4 (2)	B20—B22—B16	113.5 (3)
B8—B9—B10	58.9 (3)	B23—B22—B16	115.7 (3)
B9^vi^—B9—B10	112.6 (3)	B19—B22—B16	59.0 (3)
B7—B9—B10	106.4 (3)	B13—B22—B16	59.5 (2)
B8—B9—B11	105.6 (4)	B20—B22—B14^i^	124.0 (4)
B9^vi^—B9—B11	119.8 (3)	B23—B22—B14^i^	120.5 (3)
B7—B9—B11	104.4 (3)	B19—B22—B14^i^	118.2 (4)
B10—B9—B11	59.7 (2)	B13—B22—B14^i^	119.6 (3)
B8—B9—B1	108.5 (4)	B16—B22—B14^i^	112.2 (3)
B9^vi^—B9—B1	128.5 (4)	B20—B22—Mg1	121.5 (3)
B7—B9—B1	60.4 (2)	B23—B22—Mg1	64.9 (2)
B10—B9—B1	106.9 (3)	B19—B22—Mg1	169.6 (3)
B11—B9—B1	57.5 (2)	B13—B22—Mg1	64.2 (2)
B8—B9—Li2^ii^	112.7 (3)	B16—B22—Mg1	110.9 (3)
B9^vi^—B9—Li2^ii^	67.82 (13)	B14^i^—B22—Mg1	66.5 (2)
B7—B9—Li2^ii^	160.8 (3)	B22—B23—B20	57.7 (2)
B10—B9—Li2^ii^	58.36 (18)	B22—B23—B13	62.3 (3)
B11—B9—Li2^ii^	58.7 (2)	B20—B23—B13	106.0 (3)
B1—B9—Li2^ii^	110.3 (3)	B22—B23—B2^i^	119.6 (3)
B8—B9—Mg2^ii^	112.7 (3)	B20—B23—B2^i^	125.8 (3)
B9^vi^—B9—Mg2^ii^	67.82 (13)	B13—B23—B2^i^	119.3 (3)
B7—B9—Mg2^ii^	160.8 (3)	B22—B23—B21	105.0 (3)
B10—B9—Mg2^ii^	58.36 (18)	B20—B23—B21	60.7 (2)
B11—B9—Mg2^ii^	58.7 (2)	B13—B23—B21	101.0 (3)
B1—B9—Mg2^ii^	110.3 (3)	B2^i^—B23—B21	129.6 (3)
Li2^ii^—B9—Mg2^ii^	0.0	B22—B23—B14	104.4 (3)
B12—B10—B2	64.0 (2)	B20—B23—B14	104.2 (3)
B12—B10—B8	109.0 (3)	B13—B23—B14	55.0 (2)
B2—B10—B8	61.4 (3)	B2^i^—B23—B14	125.2 (3)
B12—B10—B4^ii^	117.5 (2)	B21—B23—B14	56.1 (2)
B2—B10—B4^ii^	117.0 (2)	B22—B23—Mg1	66.7 (3)
B8—B10—B4^ii^	125.9 (3)	B20—B23—Mg1	118.8 (3)
B12—B10—B11	55.6 (2)	B13—B23—Mg1	64.5 (2)
B2—B10—B11	108.9 (3)	B2^i^—B23—Mg1	63.9 (2)
B8—B10—B11	106.7 (3)	B21—B23—Mg1	165.2 (3)
B4^ii^—B10—B11	121.6 (2)	B14—B23—Mg1	112.7 (3)
B12—B10—B9	106.0 (3)	B25—B24—B21^xii^	66.2 (3)
B2—B10—B9	111.0 (3)	B25—B24—B17	146.1 (4)
B8—B10—B9	59.8 (3)	B21^xii^—B24—B17	109.9 (3)
B4^ii^—B10—B9	125.3 (2)	B25—B24—B1^ii^	61.2 (3)
B11—B10—B9	60.2 (2)	B21^xii^—B24—B1^ii^	120.7 (3)
B12—B10—Li1^ii^	64.3 (2)	B17—B24—B1^ii^	129.0 (3)
B2—B10—Li1^ii^	63.9 (2)	B24^vi^—B25—B24	178.3 (6)
B8—B10—Li1^ii^	120.1 (3)	B24^vi^—B25—B21^xii^	118.8 (4)
B4^ii^—B10—Li1^ii^	63.68 (18)	B24—B25—B21^xii^	59.6 (3)
B11—B10—Li1^ii^	112.5 (2)	B24^vi^—B25—B21^xiii^	59.6 (3)
B9—B10—Li1^ii^	170.1 (3)	B24—B25—B21^xiii^	118.8 (4)
B12—B10—Mg1^ii^	64.3 (2)	B21^xii^—B25—B21^xiii^	72.1 (4)
B2—B10—Mg1^ii^	63.9 (2)	B24^vi^—B25—B1^ii^	110.4 (3)
B8—B10—Mg1^ii^	120.1 (3)	B24—B25—B1^ii^	70.7 (2)
B4^ii^—B10—Mg1^ii^	63.68 (18)	B21^xii^—B25—B1^ii^	123.2 (2)
B11—B10—Mg1^ii^	112.5 (2)	B21^xiii^—B25—B1^ii^	114.8 (2)
B9—B10—Mg1^ii^	170.1 (3)	B24^vi^—B25—B1^vii^	70.7 (2)
Li1^ii^—B10—Mg1^ii^	0.0	B24—B25—B1^vii^	110.4 (3)
B12—B10—Li2^ii^	105.8 (2)	B21^xii^—B25—B1^vii^	114.8 (2)
B2—B10—Li2^ii^	169.1 (2)	B21^xiii^—B25—B1^vii^	123.2 (2)
B8—B10—Li2^ii^	128.3 (3)	B1^ii^—B25—B1^vii^	106.7 (5)
B4^ii^—B10—Li2^ii^	63.02 (16)	B23—Mg1—B22	48.4 (2)
B11—B10—Li2^ii^	65.12 (16)	B23—Mg1—B2^i^	54.2 (2)
B9—B10—Li2^ii^	74.7 (2)	B22—Mg1—B2^i^	95.8 (3)
Li1^ii^—B10—Li2^ii^	109.0 (2)	B23—Mg1—B13^i^	119.9 (3)
Mg1^ii^—B10—Li2^ii^	109.0 (2)	B22—Mg1—B13^i^	97.2 (3)
B12—B10—Mg2^ii^	105.8 (2)	B2^i^—Mg1—B13^i^	94.6 (3)
B2—B10—Mg2^ii^	169.1 (2)	B23—Mg1—B12^i^	102.9 (3)
B8—B10—Mg2^ii^	128.3 (3)	B22—Mg1—B12^i^	122.3 (3)
B4^ii^—B10—Mg2^ii^	63.02 (16)	B2^i^—Mg1—B12^i^	53.0 (2)
B11—B10—Mg2^ii^	65.12 (16)	B13^i^—Mg1—B12^i^	49.6 (2)
B9—B10—Mg2^ii^	74.7 (2)	B23—Mg1—B13	53.7 (2)
Li1^ii^—B10—Mg2^ii^	109.0 (2)	B22—Mg1—B13	52.6 (2)
Mg1^ii^—B10—Mg2^ii^	109.0 (2)	B2^i^—Mg1—B13	101.5 (3)
Li2^ii^—B10—Mg2^ii^	0.0	B13^i^—Mg1—B13	146.7 (3)
B3—B11—B12	63.8 (3)	B12^i^—Mg1—B13	154.5 (3)
B3—B11—B1	60.6 (3)	B23—Mg1—B4	112.8 (3)
B12—B11—B1	112.0 (3)	B22—Mg1—B4	140.9 (3)
B3—B11—B6^vii^	111.6 (3)	B2^i^—Mg1—B4	93.5 (2)
B12—B11—B6^vii^	117.0 (3)	B13^i^—Mg1—B4	119.8 (3)
B1—B11—B6^vii^	117.5 (3)	B12^i^—Mg1—B4	93.2 (3)
B3—B11—B10	112.0 (3)	B13—Mg1—B4	88.3 (2)
B12—B11—B10	59.6 (2)	B23—Mg1—B15^i^	138.8 (3)
B1—B11—B10	111.6 (3)	B22—Mg1—B15^i^	91.2 (3)
B6^vii^—B11—B10	125.5 (3)	B2^i^—Mg1—B15^i^	141.9 (3)
B3—B11—B9	111.7 (3)	B13^i^—Mg1—B15^i^	47.3 (2)
B12—B11—B9	109.2 (3)	B12^i^—Mg1—B15^i^	92.1 (2)
B1—B11—B9	62.8 (2)	B13—Mg1—B15^i^	112.2 (3)
B6^vii^—B11—B9	126.6 (3)	B4—Mg1—B15^i^	104.2 (3)
B10—B11—B9	60.1 (2)	B23—Mg1—B3	144.5 (3)
B3—B11—Li2^ii^	170.8 (3)	B22—Mg1—B3	117.5 (3)
B12—B11—Li2^ii^	108.2 (3)	B2^i^—Mg1—B3	144.9 (3)
B1—B11—Li2^ii^	128.4 (3)	B13^i^—Mg1—B3	92.0 (2)
B6^vii^—B11—Li2^ii^	67.3 (2)	B12^i^—Mg1—B3	110.2 (3)
B10—B11—Li2^ii^	64.44 (17)	B13—Mg1—B3	91.2 (2)
B9—B11—Li2^ii^	74.4 (2)	B4—Mg1—B3	53.9 (2)
B3—B11—Mg2^ii^	170.8 (3)	B15^i^—Mg1—B3	53.7 (2)
B12—B11—Mg2^ii^	108.2 (3)	B23—Mg1—B10^i^	96.4 (2)
B1—B11—Mg2^ii^	128.4 (3)	B22—Mg1—B10^i^	143.9 (3)
B6^vii^—B11—Mg2^ii^	67.3 (2)	B2^i^—Mg1—B10^i^	49.43 (19)
B10—B11—Mg2^ii^	64.44 (17)	B13^i^—Mg1—B10^i^	95.3 (2)
B9—B11—Mg2^ii^	74.4 (2)	B12^i^—Mg1—B10^i^	49.04 (19)
Li2^ii^—B11—Mg2^ii^	0.0	B13—Mg1—B10^i^	117.3 (3)
B11—B12—B10	64.8 (2)	B4—Mg1—B10^i^	50.60 (16)
B11—B12—B13	126.8 (3)	B15^i^—Mg1—B10^i^	121.6 (3)
B10—B12—B13	125.8 (3)	B3—Mg1—B10^i^	95.6 (3)
B11—B12—B3	57.3 (3)	B23—Mg1—B14^i^	94.6 (3)
B10—B12—B3	111.1 (3)	B22—Mg1—B14^i^	52.6 (2)
B13—B12—B3	117.8 (3)	B2^i^—Mg1—B14^i^	114.0 (3)
B11—B12—B4	111.1 (3)	B13^i^—Mg1—B14^i^	48.5 (2)
B10—B12—B4	109.1 (3)	B12^i^—Mg1—B14^i^	93.3 (2)
B13—B12—B4	111.5 (3)	B13—Mg1—B14^i^	98.2 (3)
B3—B12—B4	65.3 (2)	B4—Mg1—B14^i^	149.5 (3)
B11—B12—B2	111.7 (3)	B15^i^—Mg1—B14^i^	45.7 (2)
B10—B12—B2	58.3 (2)	B3—Mg1—B14^i^	96.0 (3)
B13—B12—B2	116.1 (3)	B10^i^—Mg1—B14^i^	142.3 (3)
B3—B12—B2	113.8 (3)	B23—Mg1—B12	97.4 (2)
B4—B12—B2	61.2 (2)	B22—Mg1—B12	95.4 (3)
B11—B12—Li1^ii^	122.1 (3)	B2^i^—Mg1—B12	122.7 (3)
B10—B12—Li1^ii^	66.7 (2)	B13^i^—Mg1—B12	139.0 (3)
B13—B12—Li1^ii^	65.2 (3)	B12^i^—Mg1—B12	141.7 (3)
B3—B12—Li1^ii^	176.9 (4)	B13—Mg1—B12	48.1 (2)
B4—B12—Li1^ii^	113.1 (3)	B4—Mg1—B12	48.77 (18)
B2—B12—Li1^ii^	63.3 (2)	B15^i^—Mg1—B12	93.6 (2)
B11—B12—Mg1^ii^	122.1 (3)	B3—Mg1—B12	47.96 (19)
B10—B12—Mg1^ii^	66.7 (2)	B10^i^—Mg1—B12	97.0 (2)
B13—B12—Mg1^ii^	65.2 (3)	B14^i^—Mg1—B12	117.2 (3)
B3—B12—Mg1^ii^	176.9 (4)	B5—Mg2—B5^iii^	59.5 (3)
B4—B12—Mg1^ii^	113.1 (3)	B5—Mg2—B4	54.84 (16)
B2—B12—Mg1^ii^	63.3 (2)	B5^iii^—Mg2—B4	108.0 (2)
Li1^ii^—B12—Mg1^ii^	0.0	B5—Mg2—B4^iii^	108.0 (2)
B11—B12—Mg1	114.0 (3)	B5^iii^—Mg2—B4^iii^	54.84 (16)
B10—B12—Mg1	170.9 (3)	B4—Mg2—B4^iii^	162.4 (3)
B13—B12—Mg1	62.7 (2)	B5—Mg2—B10^iv^	124.87 (18)
B3—B12—Mg1	63.2 (2)	B5^iii^—Mg2—B10^iv^	99.95 (15)
B4—B12—Mg1	62.4 (2)	B4—Mg2—B10^iv^	140.24 (15)
B2—B12—Mg1	116.3 (3)	B4^iii^—Mg2—B10^iv^	50.42 (11)
Li1^ii^—B12—Mg1	118.7	B5—Mg2—B10^i^	99.95 (15)
Mg1^ii^—B12—Mg1	118.7 (3)	B5^iii^—Mg2—B10^i^	124.87 (18)
B15—B13—B12	122.7 (3)	B4—Mg2—B10^i^	50.42 (11)
B15—B13—B14	57.8 (3)	B4^iii^—Mg2—B10^i^	140.24 (15)
B12—B13—B14	124.5 (3)	B10^iv^—Mg2—B10^i^	129.2 (3)
B15—B13—B16	64.1 (3)	B5—Mg2—B11^iv^	96.75 (17)
B12—B13—B16	114.1 (3)	B5^iii^—Mg2—B11^iv^	124.3 (2)
B14—B13—B16	112.4 (3)	B4—Mg2—B11^iv^	90.07 (14)
B15—B13—B22	105.9 (3)	B4^iii^—Mg2—B11^iv^	96.84 (14)
B12—B13—B22	123.0 (3)	B10^iv^—Mg2—B11^iv^	50.44 (15)
B14—B13—B22	105.2 (3)	B10^i^—Mg2—B11^iv^	107.5 (2)
B16—B13—B22	60.9 (3)	B5—Mg2—B11^i^	124.3 (2)
B15—B13—B23	108.2 (3)	B5^iii^—Mg2—B11^i^	96.75 (17)
B12—B13—B23	123.6 (3)	B4—Mg2—B11^i^	96.85 (14)
B14—B13—B23	64.2 (3)	B4^iii^—Mg2—B11^i^	90.07 (14)
B16—B13—B23	108.5 (3)	B10^iv^—Mg2—B11^i^	107.5 (2)
B22—B13—B23	54.0 (2)	B10^i^—Mg2—B11^i^	50.44 (15)
B15—B13—Li1^ii^	66.5 (2)	B11^iv^—Mg2—B11^i^	133.8 (3)
B12—B13—Li1^ii^	65.2 (3)	B5—Mg2—B6^iii^	105.9 (3)
B14—B13—Li1^ii^	68.3 (2)	B5^iii^—Mg2—B6^iii^	52.57 (18)
B16—B13—Li1^ii^	116.0 (3)	B4—Mg2—B6^iii^	125.85 (18)
B22—B13—Li1^ii^	171.7 (3)	B4^iii^—Mg2—B6^iii^	49.87 (15)
B23—B13—Li1^ii^	124.0 (3)	B10^iv^—Mg2—B6^iii^	93.55 (15)
B15—B13—Mg1^ii^	66.5 (2)	B10^i^—Mg2—B6^iii^	95.89 (15)
B12—B13—Mg1^ii^	65.2 (3)	B11^iv^—Mg2—B6^iii^	143.99 (19)
B14—B13—Mg1^ii^	68.3 (2)	B11^i^—Mg2—B6^iii^	48.83 (17)
B16—B13—Mg1^ii^	116.0 (3)	B5—Mg2—B6	52.58 (18)
B22—B13—Mg1^ii^	171.7 (3)	B5^iii^—Mg2—B6	105.9 (3)
B23—B13—Mg1^ii^	124.0 (3)	B4—Mg2—B6	49.87 (15)
Li1^ii^—B13—Mg1^ii^	0.0	B4^iii^—Mg2—B6	125.85 (18)
B15—B13—Mg1	167.9 (3)	B10^iv^—Mg2—B6	95.89 (15)
B12—B13—Mg1	69.3 (2)	B10^i^—Mg2—B6	93.55 (15)
B14—B13—Mg1	118.3 (3)	B11^iv^—Mg2—B6	48.83 (17)
B16—B13—Mg1	111.1 (3)	B11^i^—Mg2—B6	143.99 (19)
B22—B13—Mg1	63.1 (2)	B6^iii^—Mg2—B6	157.9 (4)
B23—B13—Mg1	61.8 (2)	B5—Mg2—B9^iv^	141.46 (16)
Li1^ii^—B13—Mg1	124.1	B5^iii^—Mg2—B9^iv^	145.64 (17)
Mg1^ii^—B13—Mg1	124.1 (3)	B4—Mg2—B9^iv^	105.24 (19)
B15—B14—B13	58.8 (3)	B4^iii^—Mg2—B9^iv^	91.22 (19)
B15—B14—B21	106.4 (3)	B10^iv^—Mg2—B9^iv^	46.92 (17)
B13—B14—B21	109.4 (3)	B10^i^—Mg2—B9^iv^	83.6 (2)
B15—B14—B18	59.9 (3)	B11^iv^—Mg2—B9^iv^	46.85 (17)
B13—B14—B18	109.7 (4)	B11^i^—Mg2—B9^iv^	87.5 (2)
B21—B14—B18	60.9 (3)	B6^iii^—Mg2—B9^iv^	111.9 (2)
B15—B14—B22^ii^	114.0 (3)	B6—Mg2—B9^iv^	89.01 (19)
B13—B14—B22^ii^	118.1 (3)	B5—Mg2—B9^i^	145.64 (17)
B21—B14—B22^ii^	128.5 (4)	B5^iii^—Mg2—B9^i^	141.46 (16)
B18—B14—B22^ii^	115.6 (3)	B4—Mg2—B9^i^	91.22 (19)
B15—B14—B23	106.3 (3)	B4^iii^—Mg2—B9^i^	105.24 (19)
B13—B14—B23	60.8 (3)	B10^iv^—Mg2—B9^i^	83.6 (2)
B21—B14—B23	60.7 (3)	B10^i^—Mg2—B9^i^	46.92 (17)
B18—B14—B23	110.2 (3)	B11^iv^—Mg2—B9^i^	87.5 (2)
B22^ii^—B14—B23	129.6 (3)	B11^i^—Mg2—B9^i^	46.85 (17)
B15—B14—Li1^ii^	64.6 (3)	B6^iii^—Mg2—B9^i^	89.01 (19)
B13—B14—Li1^ii^	63.2 (2)	B6—Mg2—B9^i^	111.9 (2)
B21—B14—Li1^ii^	170.2 (4)	B9^iv^—Mg2—B9^i^	44.4 (3)
B18—B14—Li1^ii^	114.3 (3)		
			
B5—B1—B3—B11	−130.2 (3)	B4—B3—B12—Mg1	70.2 (2)
B25^i^—B1—B3—B11	137.1 (4)	B10^i^—B4—B12—B11	132.8 (3)
B7—B1—B3—B11	−95.9 (3)	B5—B4—B12—B11	−2.1 (4)
B9—B1—B3—B11	−35.0 (3)	B6—B4—B12—B11	−63.9 (4)
B24^i^—B1—B3—B11	105.8 (3)	B2—B4—B12—B11	−103.9 (4)
B5—B1—B3—B12	−93.4 (3)	B3—B4—B12—B11	35.5 (3)
B11—B1—B3—B12	36.7 (3)	Mg2—B4—B12—B11	8.1 (7)
B25^i^—B1—B3—B12	173.9 (4)	Mg1—B4—B12—B11	106.9 (3)
B7—B1—B3—B12	−59.2 (4)	B10^i^—B4—B12—B10	−157.7 (3)
B9—B1—B3—B12	1.8 (4)	B5—B4—B12—B10	67.4 (3)
B24^i^—B1—B3—B12	142.5 (3)	B6—B4—B12—B10	5.6 (4)
B11—B1—B3—B5	130.2 (3)	B2—B4—B12—B10	−34.4 (3)
B25^i^—B1—B3—B5	−92.7 (4)	B3—B4—B12—B10	105.0 (3)
B7—B1—B3—B5	34.3 (3)	Mg2—B4—B12—B10	77.6 (6)
B9—B1—B3—B5	95.2 (3)	Mg1—B4—B12—B10	176.4 (3)
B24^i^—B1—B3—B5	−124.1 (3)	B10^i^—B4—B12—B13	−14.4 (4)
B5—B1—B3—B15^i^	119.5 (4)	B5—B4—B12—B13	−149.4 (3)
B11—B1—B3—B15^i^	−110.3 (4)	B6—B4—B12—B13	148.9 (3)
B25^i^—B1—B3—B15^i^	26.8 (6)	B2—B4—B12—B13	108.8 (3)
B7—B1—B3—B15^i^	153.8 (3)	B3—B4—B12—B13	−111.7 (4)
B9—B1—B3—B15^i^	−145.3 (3)	Mg2—B4—B12—B13	−139.1 (5)
B24^i^—B1—B3—B15^i^	−4.5 (4)	Mg1—B4—B12—B13	−40.3 (3)
B5—B1—B3—B4	−31.2 (3)	B10^i^—B4—B12—B3	97.3 (3)
B11—B1—B3—B4	98.9 (3)	B5—B4—B12—B3	−37.6 (3)
B25^i^—B1—B3—B4	−123.9 (4)	B6—B4—B12—B3	−99.4 (3)
B7—B1—B3—B4	3.0 (4)	B2—B4—B12—B3	−139.4 (3)
B9—B1—B3—B4	64.0 (4)	Mg2—B4—B12—B3	−27.4 (6)
B24^i^—B1—B3—B4	−155.3 (3)	Mg1—B4—B12—B3	71.4 (2)
B10—B2—B4—B12	33.4 (3)	B10^i^—B4—B12—B2	−123.2 (3)
B6—B2—B4—B12	133.0 (3)	B5—B4—B12—B2	101.8 (3)
B8—B2—B4—B12	96.2 (4)	B6—B4—B12—B2	40.0 (3)
B23^ii^—B2—B4—B12	−106.0 (4)	B3—B4—B12—B2	139.4 (3)
Li1^ii^—B2—B4—B12	−36.9 (3)	Mg2—B4—B12—B2	112.0 (5)
Mg1^ii^—B2—B4—B12	−36.9 (3)	Mg1—B4—B12—B2	−149.2 (3)
B10—B2—B4—B10^i^	153.1 (3)	B10^i^—B4—B12—Li1^ii^	−85.6 (4)
B6—B2—B4—B10^i^	−107.3 (4)	B5—B4—B12—Li1^ii^	139.4 (3)
B8—B2—B4—B10^i^	−144.1 (4)	B6—B4—B12—Li1^ii^	77.7 (3)
B12—B2—B4—B10^i^	119.7 (4)	B2—B4—B12—Li1^ii^	37.6 (3)
B23^ii^—B2—B4—B10^i^	13.7 (6)	B3—B4—B12—Li1^ii^	177.1 (4)
Li1^ii^—B2—B4—B10^i^	82.7 (4)	Mg2—B4—B12—Li1^ii^	149.7 (4)
Mg1^ii^—B2—B4—B10^i^	82.7 (4)	Mg1—B4—B12—Li1^ii^	−111.5 (3)
B10—B2—B4—B5	−60.2 (3)	B10^i^—B4—B12—Mg1^ii^	−85.6 (4)
B6—B2—B4—B5	39.4 (3)	B5—B4—B12—Mg1^ii^	139.4 (3)
B8—B2—B4—B5	2.6 (4)	B6—B4—B12—Mg1^ii^	77.7 (3)
B12—B2—B4—B5	−93.6 (3)	B2—B4—B12—Mg1^ii^	37.6 (3)
B23^ii^—B2—B4—B5	160.4 (3)	B3—B4—B12—Mg1^ii^	177.1 (4)
Li1^ii^—B2—B4—B5	−130.5 (3)	Mg2—B4—B12—Mg1^ii^	149.7 (4)
Mg1^ii^—B2—B4—B5	−130.5 (3)	Mg1—B4—B12—Mg1^ii^	−111.5 (3)
B10—B2—B4—B6	−99.6 (3)	B10^i^—B4—B12—Mg1	25.9 (3)
B8—B2—B4—B6	−36.8 (3)	B5—B4—B12—Mg1	−109.0 (2)
B12—B2—B4—B6	−133.0 (3)	B6—B4—B12—Mg1	−170.8 (3)
B23^ii^—B2—B4—B6	121.0 (4)	B2—B4—B12—Mg1	149.2 (3)
Li1^ii^—B2—B4—B6	−169.9 (4)	B3—B4—B12—Mg1	−71.4 (2)
Mg1^ii^—B2—B4—B6	−169.9 (4)	Mg2—B4—B12—Mg1	−98.8 (5)
B10—B2—B4—B3	−1.4 (3)	B10—B2—B12—B11	−38.2 (3)
B6—B2—B4—B3	98.2 (3)	B6—B2—B12—B11	62.3 (4)
B8—B2—B4—B3	61.5 (4)	B8—B2—B12—B11	2.7 (5)
B12—B2—B4—B3	−34.8 (3)	B4—B2—B12—B11	102.9 (3)
B23^ii^—B2—B4—B3	−140.8 (3)	B23^ii^—B2—B12—B11	−143.7 (3)
Li1^ii^—B2—B4—B3	−71.7 (3)	Li1^ii^—B2—B12—B11	−116.1 (3)
Mg1^ii^—B2—B4—B3	−71.7 (3)	Mg1^ii^—B2—B12—B11	−116.1 (3)
B10—B2—B4—Mg2	−118.2 (3)	B6—B2—B12—B10	100.6 (3)
B6—B2—B4—Mg2	−18.6 (3)	B8—B2—B12—B10	41.0 (3)
B8—B2—B4—Mg2	−55.3 (4)	B4—B2—B12—B10	141.1 (3)
B12—B2—B4—Mg2	−151.6 (3)	B23^ii^—B2—B12—B10	−105.5 (4)
B23^ii^—B2—B4—Mg2	102.4 (4)	Li1^ii^—B2—B12—B10	−77.8 (2)
Li1^ii^—B2—B4—Mg2	171.5 (3)	Mg1^ii^—B2—B12—B10	−77.8 (2)
Mg1^ii^—B2—B4—Mg2	171.5 (3)	B10—B2—B12—B13	117.6 (4)
B10—B2—B4—Mg1	67.8 (4)	B6—B2—B12—B13	−141.8 (3)
B6—B2—B4—Mg1	167.4 (3)	B8—B2—B12—B13	158.5 (4)
B8—B2—B4—Mg1	130.6 (4)	B4—B2—B12—B13	−101.3 (4)
B12—B2—B4—Mg1	34.4 (3)	B23^ii^—B2—B12—B13	12.1 (5)
B23^ii^—B2—B4—Mg1	−71.6 (5)	Li1^ii^—B2—B12—B13	39.7 (3)
Li1^ii^—B2—B4—Mg1	−2.5 (4)	Mg1^ii^—B2—B12—B13	39.7 (3)
Mg1^ii^—B2—B4—Mg1	−2.5 (4)	B10—B2—B12—B3	−100.9 (3)
B11—B3—B4—B12	−35.1 (3)	B6—B2—B12—B3	−0.3 (4)
B1—B3—B4—B12	−102.8 (3)	B8—B2—B12—B3	−60.0 (5)
B5—B3—B4—B12	−133.8 (3)	B4—B2—B12—B3	40.2 (3)
B15^i^—B3—B4—B12	105.9 (4)	B23^ii^—B2—B12—B3	153.6 (3)
Mg1—B3—B4—B12	81.0 (3)	Li1^ii^—B2—B12—B3	−178.8 (4)
B11—B3—B4—B10^i^	−157.5 (3)	Mg1^ii^—B2—B12—B3	−178.8 (4)
B1—B3—B4—B10^i^	134.8 (3)	B10—B2—B12—B4	−141.1 (3)
B12—B3—B4—B10^i^	−122.5 (3)	B6—B2—B12—B4	−40.5 (3)
B5—B3—B4—B10^i^	103.8 (3)	B8—B2—B12—B4	−100.1 (4)
B15^i^—B3—B4—B10^i^	−16.5 (4)	B23^ii^—B2—B12—B4	113.4 (4)
Mg1—B3—B4—B10^i^	−41.4 (3)	Li1^ii^—B2—B12—B4	141.0 (3)
B11—B3—B4—B5	98.7 (3)	Mg1^ii^—B2—B12—B4	141.0 (3)
B1—B3—B4—B5	31.0 (3)	B10—B2—B12—Li1^ii^	77.8 (2)
B12—B3—B4—B5	133.8 (3)	B6—B2—B12—Li1^ii^	178.4 (3)
B15^i^—B3—B4—B5	−120.3 (4)	B8—B2—B12—Li1^ii^	118.8 (4)
Mg1—B3—B4—B5	−145.2 (3)	B4—B2—B12—Li1^ii^	−141.0 (3)
B11—B3—B4—B6	62.7 (4)	B23^ii^—B2—B12—Li1^ii^	−27.6 (3)
B1—B3—B4—B6	−4.9 (4)	Mg1^ii^—B2—B12—Li1^ii^	0.000 (1)
B12—B3—B4—B6	97.8 (3)	B10—B2—B12—Mg1^ii^	77.8 (2)
B5—B3—B4—B6	−35.9 (3)	B6—B2—B12—Mg1^ii^	178.4 (3)
B15^i^—B3—B4—B6	−156.3 (3)	B8—B2—B12—Mg1^ii^	118.8 (4)
Mg1—B3—B4—B6	178.9 (3)	B4—B2—B12—Mg1^ii^	−141.0 (3)
B11—B3—B4—B2	1.8 (4)	B23^ii^—B2—B12—Mg1^ii^	−27.6 (3)
B1—B3—B4—B2	−65.9 (4)	Li1^ii^—B2—B12—Mg1^ii^	0.000 (1)
B12—B3—B4—B2	36.8 (3)	B10—B2—B12—Mg1	−171.6 (3)
B5—B3—B4—B2	−96.9 (3)	B6—B2—B12—Mg1	−71.0 (4)
B15^i^—B3—B4—B2	142.8 (3)	B8—B2—B12—Mg1	−130.6 (4)
Mg1—B3—B4—B2	117.9 (3)	B4—B2—B12—Mg1	−30.5 (3)
B11—B3—B4—Mg2	133.5 (3)	B23^ii^—B2—B12—Mg1	83.0 (4)
B1—B3—B4—Mg2	65.9 (3)	Li1^ii^—B2—B12—Mg1	110.6 (4)
B12—B3—B4—Mg2	168.6 (3)	Mg1^ii^—B2—B12—Mg1	110.6 (4)
B5—B3—B4—Mg2	34.85 (19)	B11—B12—B13—B15	77.5 (5)
B15^i^—B3—B4—Mg2	−85.5 (3)	B10—B12—B13—B15	−5.7 (6)
Mg1—B3—B4—Mg2	−110.4 (2)	B3—B12—B13—B15	146.0 (4)
B11—B3—B4—Mg1	−116.1 (3)	B4—B12—B13—B15	−141.5 (4)
B1—B3—B4—Mg1	176.2 (3)	B2—B12—B13—B15	−74.1 (5)
B12—B3—B4—Mg1	−81.0 (3)	Li1^ii^—B12—B13—B15	−35.1 (4)
B5—B3—B4—Mg1	145.2 (3)	Mg1^ii^—B12—B13—B15	−35.1 (4)
B15^i^—B3—B4—Mg1	24.9 (3)	Mg1—B12—B13—B15	178.3 (5)
B3—B1—B5—B7	140.2 (3)	B11—B12—B13—B14	148.5 (4)
B11—B1—B5—B7	100.4 (3)	B10—B12—B13—B14	65.2 (6)
B25^i^—B1—B5—B7	−104.0 (3)	B3—B12—B13—B14	−143.1 (4)
B9—B1—B5—B7	39.3 (3)	B4—B12—B13—B14	−70.6 (5)
B24^i^—B1—B5—B7	−154.9 (4)	B2—B12—B13—B14	−3.2 (6)
B11—B1—B5—B3	−39.8 (3)	Li1^ii^—B12—B13—B14	35.8 (4)
B25^i^—B1—B5—B3	115.8 (3)	Mg1^ii^—B12—B13—B14	35.8 (4)
B7—B1—B5—B3	−140.2 (3)	Mg1—B12—B13—B14	−110.8 (4)
B9—B1—B5—B3	−100.9 (3)	B11—B12—B13—B16	3.9 (6)
B24^i^—B1—B5—B3	65.0 (3)	B10—B12—B13—B16	−79.4 (5)
B3—B1—B5—B5^iii^	−108.0 (4)	B3—B12—B13—B16	72.3 (5)
B11—B1—B5—B5^iii^	−147.9 (3)	B4—B12—B13—B16	144.8 (3)
B25^i^—B1—B5—B5^iii^	7.8 (4)	B2—B12—B13—B16	−147.7 (3)
B7—B1—B5—B5^iii^	111.8 (4)	Li1^ii^—B12—B13—B16	−108.8 (3)
B9—B1—B5—B5^iii^	151.0 (3)	Mg1^ii^—B12—B13—B16	−108.8 (3)
B24^i^—B1—B5—B5^iii^	−43.1 (4)	Mg1—B12—B13—B16	104.6 (4)
B3—B1—B5—B4	34.5 (3)	B11—B12—B13—B22	−65.9 (6)
B11—B1—B5—B4	−5.4 (4)	B10—B12—B13—B22	−149.2 (3)
B25^i^—B1—B5—B4	150.3 (2)	B3—B12—B13—B22	2.5 (5)
B7—B1—B5—B4	−105.7 (3)	B4—B12—B13—B22	75.0 (4)
B9—B1—B5—B4	−66.4 (4)	B2—B12—B13—B22	142.4 (4)
B24^i^—B1—B5—B4	99.4 (3)	Li1^ii^—B12—B13—B22	−178.6 (4)
B3—B1—B5—B6	101.8 (3)	Mg1^ii^—B12—B13—B22	−178.6 (4)
B11—B1—B5—B6	61.9 (4)	Mg1—B12—B13—B22	34.8 (3)
B25^i^—B1—B5—B6	−142.4 (3)	B11—B12—B13—B23	−131.7 (4)
B7—B1—B5—B6	−38.4 (3)	B10—B12—B13—B23	145.0 (3)
B9—B1—B5—B6	0.9 (4)	B3—B12—B13—B23	−63.3 (5)
B24^i^—B1—B5—B6	166.7 (3)	B4—B12—B13—B23	9.2 (5)
B3—B1—B5—Mg2	−83.5 (15)	B2—B12—B13—B23	76.6 (5)
B11—B1—B5—Mg2	−123.3 (14)	Li1^ii^—B12—B13—B23	115.6 (4)
B25^i^—B1—B5—Mg2	32.3 (16)	Mg1^ii^—B12—B13—B23	115.6 (4)
B7—B1—B5—Mg2	136.3 (15)	Mg1—B12—B13—B23	−31.0 (3)
B9—B1—B5—Mg2	175.6 (14)	B11—B12—B13—Li1^ii^	112.7 (4)
B24^i^—B1—B5—Mg2	−18.6 (16)	B10—B12—B13—Li1^ii^	29.4 (4)
B11—B3—B5—B1	45.5 (3)	B3—B12—B13—Li1^ii^	−178.9 (4)
B12—B3—B5—B1	106.5 (3)	B4—B12—B13—Li1^ii^	−106.4 (3)
B15^i^—B3—B5—B1	−108.2 (4)	B2—B12—B13—Li1^ii^	−39.0 (3)
B4—B3—B5—B1	145.5 (3)	Mg1^ii^—B12—B13—Li1^ii^	0.000 (1)
Mg1—B3—B5—B1	178.9 (3)	Mg1—B12—B13—Li1^ii^	−146.6 (3)
B11—B3—B5—B7	5.5 (4)	B11—B12—B13—Mg1^ii^	112.7 (4)
B1—B3—B5—B7	−40.0 (3)	B10—B12—B13—Mg1^ii^	29.4 (4)
B12—B3—B5—B7	66.5 (4)	B3—B12—B13—Mg1^ii^	−178.9 (4)
B15^i^—B3—B5—B7	−148.2 (4)	B4—B12—B13—Mg1^ii^	−106.4 (3)
B4—B3—B5—B7	105.5 (3)	B2—B12—B13—Mg1^ii^	−39.0 (3)
Mg1—B3—B5—B7	138.9 (3)	Li1^ii^—B12—B13—Mg1^ii^	0.000 (1)
B11—B3—B5—B5^iii^	145.2 (2)	Mg1—B12—B13—Mg1^ii^	−146.6 (3)
B1—B3—B5—B5^iii^	99.7 (2)	B11—B12—B13—Mg1	−100.7 (4)
B12—B3—B5—B5^iii^	−153.8 (2)	B10—B12—B13—Mg1	176.0 (4)
B15^i^—B3—B5—B5^iii^	−8.4 (5)	B3—B12—B13—Mg1	−32.3 (3)
B4—B3—B5—B5^iii^	−114.8 (2)	B4—B12—B13—Mg1	40.2 (3)
Mg1—B3—B5—B5^iii^	−81.3 (3)	B2—B12—B13—Mg1	107.6 (4)
B11—B3—B5—B4	−100.0 (3)	Li1^ii^—B12—B13—Mg1	146.6 (3)
B1—B3—B5—B4	−145.5 (3)	Mg1^ii^—B12—B13—Mg1	146.6 (3)
B12—B3—B5—B4	−39.0 (3)	B12—B13—B14—B15	−110.0 (4)
B15^i^—B3—B5—B4	106.4 (4)	B16—B13—B14—B15	35.1 (3)
Mg1—B3—B5—B4	33.4 (3)	B22—B13—B14—B15	99.4 (3)
B11—B3—B5—B6	−62.6 (4)	B23—B13—B14—B15	135.6 (3)
B1—B3—B5—B6	−108.1 (3)	Li1^ii^—B13—B14—B15	−75.1 (3)
B12—B3—B5—B6	−1.6 (4)	Mg1^ii^—B13—B14—B15	−75.1 (3)
B15^i^—B3—B5—B6	143.7 (4)	Mg1—B13—B14—B15	166.8 (4)
B4—B3—B5—B6	37.4 (3)	B15—B13—B14—B21	−97.9 (4)
Mg1—B3—B5—B6	70.8 (4)	B12—B13—B14—B21	152.1 (4)
B11—B3—B5—Mg2	−146.7 (3)	B16—B13—B14—B21	−62.8 (4)
B1—B3—B5—Mg2	167.9 (3)	B22—B13—B14—B21	1.5 (4)
B12—B3—B5—Mg2	−85.6 (3)	B23—B13—B14—B21	37.7 (3)
B15^i^—B3—B5—Mg2	59.7 (5)	Li1^ii^—B13—B14—B21	−173.0 (4)
B4—B3—B5—Mg2	−46.6 (2)	Mg1^ii^—B13—B14—B21	−173.0 (4)
Mg1—B3—B5—Mg2	−13.2 (4)	Mg1—B13—B14—B21	68.9 (4)
B12—B4—B5—B1	4.9 (4)	B15—B13—B14—B18	−32.9 (3)
B10^i^—B4—B5—B1	−138.4 (3)	B12—B13—B14—B18	−142.9 (4)
B6—B4—B5—B1	106.4 (3)	B16—B13—B14—B18	2.2 (4)
B2—B4—B5—B1	68.0 (3)	B22—B13—B14—B18	66.6 (4)
B3—B4—B5—B1	−33.0 (3)	B23—B13—B14—B18	102.7 (3)
Mg2—B4—B5—B1	−169.9 (3)	Li1^ii^—B13—B14—B18	−108.0 (3)
Mg1—B4—B5—B1	−66.3 (3)	Mg1^ii^—B13—B14—B18	−108.0 (3)
B12—B4—B5—B7	−68.9 (3)	Mg1—B13—B14—B18	133.9 (3)
B10^i^—B4—B5—B7	147.8 (3)	B15—B13—B14—B22^ii^	102.5 (4)
B6—B4—B5—B7	32.6 (3)	B12—B13—B14—B22^ii^	−7.5 (6)
B2—B4—B5—B7	−5.7 (4)	B16—B13—B14—B22^ii^	137.6 (4)
B3—B4—B5—B7	−106.8 (3)	B22—B13—B14—B22^ii^	−158.1 (3)
Mg2—B4—B5—B7	116.3 (3)	B23—B13—B14—B22^ii^	−121.9 (4)
Mg1—B4—B5—B7	−140.0 (3)	Li1^ii^—B13—B14—B22^ii^	27.4 (3)
B12—B4—B5—B3	37.9 (3)	Mg1^ii^—B13—B14—B22^ii^	27.4 (3)
B10^i^—B4—B5—B3	−105.4 (3)	Mg1—B13—B14—B22^ii^	−90.7 (4)
B6—B4—B5—B3	139.5 (3)	B15—B13—B14—B23	−135.6 (3)
B2—B4—B5—B3	101.1 (3)	B12—B13—B14—B23	114.4 (4)
Mg2—B4—B5—B3	−136.9 (2)	B16—B13—B14—B23	−100.5 (3)
Mg1—B4—B5—B3	−33.2 (3)	B22—B13—B14—B23	−36.2 (3)
B12—B4—B5—B5^iii^	142.7 (3)	Li1^ii^—B13—B14—B23	149.3 (3)
B10^i^—B4—B5—B5^iii^	−0.7 (4)	Mg1^ii^—B13—B14—B23	149.3 (3)
B6—B4—B5—B5^iii^	−115.8 (4)	Mg1—B13—B14—B23	31.2 (3)
B2—B4—B5—B5^iii^	−154.2 (3)	B15—B13—B14—Li1^ii^	75.1 (3)
B3—B4—B5—B5^iii^	104.8 (4)	B12—B13—B14—Li1^ii^	−34.9 (4)
Mg2—B4—B5—B5^iii^	−32.1 (2)	B16—B13—B14—Li1^ii^	110.2 (3)
Mg1—B4—B5—B5^iii^	71.5 (4)	B22—B13—B14—Li1^ii^	174.6 (3)
B12—B4—B5—B6	−101.6 (3)	B23—B13—B14—Li1^ii^	−149.3 (3)
B10^i^—B4—B5—B6	115.1 (3)	Mg1^ii^—B13—B14—Li1^ii^	0.000 (1)
B2—B4—B5—B6	−38.4 (3)	Mg1—B13—B14—Li1^ii^	−118.1 (3)
B3—B4—B5—B6	−139.5 (3)	B15—B13—B14—Mg1^ii^	75.1 (3)
Mg2—B4—B5—B6	83.7 (2)	B12—B13—B14—Mg1^ii^	−34.9 (4)
Mg1—B4—B5—B6	−172.7 (3)	B16—B13—B14—Mg1^ii^	110.2 (3)
B12—B4—B5—Mg2	174.8 (2)	B22—B13—B14—Mg1^ii^	174.6 (3)
B10^i^—B4—B5—Mg2	31.4 (2)	B23—B13—B14—Mg1^ii^	−149.3 (3)
B6—B4—B5—Mg2	−83.7 (2)	Li1^ii^—B13—B14—Mg1^ii^	0.000 (1)
B2—B4—B5—Mg2	−122.1 (2)	Mg1—B13—B14—Mg1^ii^	−118.1 (3)
B3—B4—B5—Mg2	136.9 (2)	B13—B14—B15—B17	−110.1 (4)
Mg1—B4—B5—Mg2	103.6 (2)	B21—B14—B15—B17	−7.0 (5)
B10—B2—B6—B8	−38.3 (4)	B18—B14—B15—B17	33.7 (4)
B4—B2—B6—B8	−137.1 (4)	B22^ii^—B14—B15—B17	140.5 (4)
B12—B2—B6—B8	−98.0 (4)	B23—B14—B15—B17	−70.6 (5)
B23^ii^—B2—B6—B8	112.1 (5)	Li1^ii^—B14—B15—B17	177.2 (4)
Li1^ii^—B2—B6—B8	−91.9 (15)	Mg1^ii^—B14—B15—B17	177.2 (4)
Mg1^ii^—B2—B6—B8	−91.9 (15)	B21—B14—B15—B13	103.1 (4)
B10—B2—B6—B11^iv^	−164.7 (4)	B18—B14—B15—B13	143.8 (3)
B8—B2—B6—B11^iv^	−126.4 (5)	B22^ii^—B14—B15—B13	−109.5 (4)
B4—B2—B6—B11^iv^	96.5 (4)	B23—B14—B15—B13	39.5 (3)
B12—B2—B6—B11^iv^	135.6 (4)	Li1^ii^—B14—B15—B13	−72.7 (3)
B23^ii^—B2—B6—B11^iv^	−14.3 (6)	Mg1^ii^—B14—B15—B13	−72.7 (3)
Li1^ii^—B2—B6—B11^iv^	141.7 (14)	B13—B14—B15—B18	−143.8 (3)
Mg1^ii^—B2—B6—B11^iv^	141.7 (14)	B21—B14—B15—B18	−40.7 (3)
B10—B2—B6—B4	98.8 (3)	B22^ii^—B14—B15—B18	106.7 (4)
B8—B2—B6—B4	137.1 (4)	B23—B14—B15—B18	−104.3 (3)
B12—B2—B6—B4	39.1 (3)	Li1^ii^—B14—B15—B18	143.4 (3)
B23^ii^—B2—B6—B4	−110.8 (4)	Mg1^ii^—B14—B15—B18	143.4 (3)
Li1^ii^—B2—B6—B4	45.2 (15)	B13—B14—B15—B16	−35.4 (3)
Mg1^ii^—B2—B6—B4	45.2 (15)	B21—B14—B15—B16	67.7 (4)
B10—B2—B6—B5	59.1 (4)	B18—B14—B15—B16	108.4 (4)
B8—B2—B6—B5	97.3 (4)	B22^ii^—B14—B15—B16	−144.8 (3)
B4—B2—B6—B5	−39.8 (3)	B23—B14—B15—B16	4.1 (5)
B12—B2—B6—B5	−0.7 (4)	Li1^ii^—B14—B15—B16	−108.1 (4)
B23^ii^—B2—B6—B5	−150.5 (4)	Mg1^ii^—B14—B15—B16	−108.1 (4)
Li1^ii^—B2—B6—B5	5.4 (16)	B13—B14—B15—B3^ii^	102.4 (4)
Mg1^ii^—B2—B6—B5	5.4 (16)	B21—B14—B15—B3^ii^	−154.5 (4)
B10—B2—B6—B7	−0.9 (4)	B18—B14—B15—B3^ii^	−113.8 (5)
B8—B2—B6—B7	37.4 (4)	B22^ii^—B14—B15—B3^ii^	−7.0 (6)
B4—B2—B6—B7	−99.8 (3)	B23—B14—B15—B3^ii^	141.9 (4)
B12—B2—B6—B7	−60.7 (4)	Li1^ii^—B14—B15—B3^ii^	29.7 (4)
B23^ii^—B2—B6—B7	149.5 (4)	Mg1^ii^—B14—B15—B3^ii^	29.7 (4)
Li1^ii^—B2—B6—B7	−54.6 (16)	B13—B14—B15—Li1^ii^	72.7 (3)
Mg1^ii^—B2—B6—B7	−54.6 (16)	B21—B14—B15—Li1^ii^	175.8 (3)
B10—B2—B6—Mg2	115.5 (4)	B18—B14—B15—Li1^ii^	−143.4 (3)
B8—B2—B6—Mg2	153.8 (5)	B22^ii^—B14—B15—Li1^ii^	−36.7 (3)
B4—B2—B6—Mg2	16.7 (3)	B23—B14—B15—Li1^ii^	112.3 (3)
B12—B2—B6—Mg2	55.8 (4)	Mg1^ii^—B14—B15—Li1^ii^	0.0
B23^ii^—B2—B6—Mg2	−94.1 (5)	B13—B14—B15—Mg1^ii^	72.7 (3)
Li1^ii^—B2—B6—Mg2	61.9 (16)	B21—B14—B15—Mg1^ii^	175.8 (3)
Mg1^ii^—B2—B6—Mg2	61.9 (16)	B18—B14—B15—Mg1^ii^	−143.4 (3)
B12—B4—B6—B8	−1.9 (4)	B22^ii^—B14—B15—Mg1^ii^	−36.7 (3)
B10^i^—B4—B6—B8	162.9 (3)	B23—B14—B15—Mg1^ii^	112.3 (3)
B5—B4—B6—B8	−95.3 (4)	Li1^ii^—B14—B15—Mg1^ii^	0.0
B2—B4—B6—B8	39.9 (4)	B12—B13—B15—B17	−139.0 (4)
B3—B4—B6—B8	−61.0 (4)	B14—B13—B15—B17	108.0 (4)
Mg2—B4—B6—B8	−156.4 (4)	B16—B13—B15—B17	−35.8 (4)
Mg1—B4—B6—B8	−55.5 (15)	B22—B13—B15—B17	9.7 (5)
B12—B4—B6—B2	−41.8 (3)	B23—B13—B15—B17	66.4 (5)
B10^i^—B4—B6—B2	123.0 (3)	Li1^ii^—B13—B15—B17	−173.7 (4)
B5—B4—B6—B2	−135.1 (3)	Mg1^ii^—B13—B15—B17	−173.7 (4)
B3—B4—B6—B2	−100.9 (3)	Mg1—B13—B15—B17	33.2 (19)
Mg2—B4—B6—B2	163.7 (3)	B12—B13—B15—B14	113.0 (4)
Mg1—B4—B6—B2	−95.4 (14)	B16—B13—B15—B14	−143.8 (3)
B12—B4—B6—B11^iv^	−164.9 (3)	B22—B13—B15—B14	−98.2 (3)
B10^i^—B4—B6—B11^iv^	−0.1 (4)	B23—B13—B15—B14	−41.6 (3)
B5—B4—B6—B11^iv^	101.8 (3)	Li1^ii^—B13—B15—B14	78.3 (3)
B2—B4—B6—B11^iv^	−123.1 (4)	Mg1^ii^—B13—B15—B14	78.3 (3)
B3—B4—B6—B11^iv^	136.0 (3)	Mg1—B13—B15—B14	−74.7 (17)
Mg2—B4—B6—B11^iv^	40.6 (3)	B12—B13—B15—B18	150.1 (4)
Mg1—B4—B6—B11^iv^	141.6 (13)	B14—B13—B15—B18	37.0 (4)
B12—B4—B6—B5	93.4 (3)	B16—B13—B15—B18	−106.7 (4)
B10^i^—B4—B6—B5	−101.9 (3)	B22—B13—B15—B18	−61.2 (5)
B2—B4—B6—B5	135.1 (3)	B23—B13—B15—B18	−4.5 (5)
B3—B4—B6—B5	34.2 (2)	Li1^ii^—B13—B15—B18	115.3 (4)
Mg2—B4—B6—B5	−61.1 (2)	Mg1^ii^—B13—B15—B18	115.3 (4)
Mg1—B4—B6—B5	39.8 (14)	Mg1—B13—B15—B18	−37.7 (19)
B12—B4—B6—B7	63.9 (3)	B12—B13—B15—B16	−103.2 (4)
B10^i^—B4—B6—B7	−131.3 (3)	B14—B13—B15—B16	143.8 (3)
B5—B4—B6—B7	−29.4 (3)	B22—B13—B15—B16	45.5 (3)
B2—B4—B6—B7	105.7 (3)	B23—B13—B15—B16	102.2 (3)
B3—B4—B6—B7	4.8 (4)	Li1^ii^—B13—B15—B16	−137.9 (3)
Mg2—B4—B6—B7	−90.6 (3)	Mg1^ii^—B13—B15—B16	−137.9 (3)
Mg1—B4—B6—B7	10.3 (15)	Mg1—B13—B15—B16	69.0 (17)
B12—B4—B6—Mg2	154.5 (3)	B12—B13—B15—B3^ii^	−7.2 (5)
B10^i^—B4—B6—Mg2	−40.7 (3)	B14—B13—B15—B3^ii^	−120.3 (4)
B5—B4—B6—Mg2	61.1 (2)	B16—B13—B15—B3^ii^	96.0 (4)
B2—B4—B6—Mg2	−163.7 (3)	B22—B13—B15—B3^ii^	141.5 (3)
B3—B4—B6—Mg2	95.4 (2)	B23—B13—B15—B3^ii^	−161.8 (3)
Mg1—B4—B6—Mg2	100.9 (14)	Li1^ii^—B13—B15—B3^ii^	−42.0 (3)
B1—B5—B6—B8	−0.3 (5)	Mg1^ii^—B13—B15—B3^ii^	−42.0 (3)
B7—B5—B6—B8	−40.4 (4)	Mg1—B13—B15—B3^ii^	165.0 (16)
B3—B5—B6—B8	66.2 (4)	B12—B13—B15—Li1^ii^	34.7 (4)
B5^iii^—B5—B6—B8	−145.2 (4)	B14—B13—B15—Li1^ii^	−78.3 (3)
B4—B5—B6—B8	105.5 (4)	B16—B13—B15—Li1^ii^	137.9 (3)
Mg2—B5—B6—B8	−179.3 (4)	B22—B13—B15—Li1^ii^	−176.5 (3)
B1—B5—B6—B2	−65.0 (4)	B23—B13—B15—Li1^ii^	−119.9 (3)
B7—B5—B6—B2	−105.1 (3)	Mg1^ii^—B13—B15—Li1^ii^	0.000 (1)
B3—B5—B6—B2	1.5 (4)	Mg1—B13—B15—Li1^ii^	−153.0 (17)
B5^iii^—B5—B6—B2	150.1 (3)	B12—B13—B15—Mg1^ii^	34.7 (4)
B4—B5—B6—B2	40.7 (3)	B14—B13—B15—Mg1^ii^	−78.3 (3)
Mg2—B5—B6—B2	116.0 (3)	B16—B13—B15—Mg1^ii^	137.9 (3)
B1—B5—B6—B11^iv^	150.3 (3)	B22—B13—B15—Mg1^ii^	−176.5 (3)
B7—B5—B6—B11^iv^	110.2 (4)	B23—B13—B15—Mg1^ii^	−119.9 (3)
B3—B5—B6—B11^iv^	−143.2 (3)	Li1^ii^—B13—B15—Mg1^ii^	0.000 (1)
B5^iii^—B5—B6—B11^iv^	5.4 (5)	Mg1—B13—B15—Mg1^ii^	−153.0 (17)
B4—B5—B6—B11^iv^	−103.9 (3)	B15—B13—B16—B7^vii^	−110.3 (4)
Mg2—B5—B6—B11^iv^	−28.7 (3)	B12—B13—B16—B7^vii^	5.8 (5)
B1—B5—B6—B4	−105.8 (3)	B14—B13—B16—B7^vii^	−143.1 (4)
B7—B5—B6—B4	−145.9 (3)	B22—B13—B16—B7^vii^	121.4 (4)
B3—B5—B6—B4	−39.2 (3)	B23—B13—B16—B7^vii^	147.9 (3)
B5^iii^—B5—B6—B4	109.3 (3)	Li1^ii^—B13—B16—B7^vii^	−67.2 (5)
Mg2—B5—B6—B4	75.26 (18)	Mg1^ii^—B13—B16—B7^vii^	−67.2 (5)
B1—B5—B6—B7	40.1 (3)	Mg1—B13—B16—B7^vii^	81.7 (4)
B3—B5—B6—B7	106.6 (3)	B15—B13—B16—B19	90.5 (3)
B5^iii^—B5—B6—B7	−104.8 (4)	B12—B13—B16—B19	−153.4 (3)
B4—B5—B6—B7	145.9 (3)	B14—B13—B16—B19	57.7 (4)
Mg2—B5—B6—B7	−138.9 (3)	B22—B13—B16—B19	−37.7 (3)
B1—B5—B6—Mg2	179.0 (3)	B23—B13—B16—B19	−11.3 (4)
B7—B5—B6—Mg2	138.9 (3)	Li1^ii^—B13—B16—B19	133.6 (3)
B3—B5—B6—Mg2	−114.5 (3)	Mg1^ii^—B13—B16—B19	133.6 (3)
B5^iii^—B5—B6—Mg2	34.1 (3)	Mg1—B13—B16—B19	−77.4 (4)
B4—B5—B6—Mg2	−75.26 (18)	B15—B13—B16—B22	128.2 (3)
B1—B5—B7—B16^iv^	112.5 (5)	B12—B13—B16—B22	−115.7 (4)
B3—B5—B7—B16^iv^	150.0 (4)	B14—B13—B16—B22	95.5 (3)
B5^iii^—B5—B7—B16^iv^	11.2 (5)	B23—B13—B16—B22	26.4 (3)
B4—B5—B7—B16^iv^	−138.7 (4)	Li1^ii^—B13—B16—B22	171.4 (4)
B6—B5—B7—B16^iv^	−106.8 (5)	Mg1^ii^—B13—B16—B22	171.4 (4)
Mg2—B5—B7—B16^iv^	−58.9 (5)	Mg1—B13—B16—B22	−39.7 (3)
B1—B5—B7—B6	−140.7 (3)	B12—B13—B16—B15	116.1 (4)
B3—B5—B7—B6	−103.1 (3)	B14—B13—B16—B15	−32.7 (3)
B5^iii^—B5—B7—B6	118.0 (3)	B22—B13—B16—B15	−128.2 (3)
B4—B5—B7—B6	−31.9 (3)	B23—B13—B16—B15	−101.8 (3)
Mg2—B5—B7—B6	48.0 (3)	Li1^ii^—B13—B16—B15	43.1 (3)
B1—B5—B7—B8	−102.5 (4)	Mg1^ii^—B13—B16—B15	43.1 (3)
B3—B5—B7—B8	−65.0 (4)	Mg1—B13—B16—B15	−167.9 (4)
B5^iii^—B5—B7—B8	156.2 (3)	B15—B13—B16—B17	26.5 (3)
B4—B5—B7—B8	6.3 (4)	B12—B13—B16—B17	142.7 (3)
B6—B5—B7—B8	38.2 (3)	B14—B13—B16—B17	−6.2 (4)
Mg2—B5—B7—B8	86.2 (4)	B22—B13—B16—B17	−101.7 (3)
B1—B5—B7—B9	−41.2 (3)	B23—B13—B16—B17	−75.2 (3)
B3—B5—B7—B9	−3.6 (4)	Li1^ii^—B13—B16—B17	69.7 (4)
B5^iii^—B5—B7—B9	−142.4 (2)	Mg1^ii^—B13—B16—B17	69.7 (4)
B4—B5—B7—B9	67.6 (4)	Mg1—B13—B16—B17	−141.4 (3)
B6—B5—B7—B9	99.6 (3)	B17—B15—B16—B7^vii^	−106.9 (4)
Mg2—B5—B7—B9	147.5 (3)	B14—B15—B16—B7^vii^	143.1 (4)
B3—B5—B7—B1	37.6 (3)	B13—B15—B16—B7^vii^	107.3 (4)
B5^iii^—B5—B7—B1	−101.3 (2)	B18—B15—B16—B7^vii^	−143.8 (4)
B4—B5—B7—B1	108.8 (3)	B3^ii^—B15—B16—B7^vii^	−2.8 (5)
B6—B5—B7—B1	140.7 (3)	Li1^ii^—B15—B16—B7^vii^	65.0 (5)
Mg2—B5—B7—B1	−171.3 (3)	Mg1^ii^—B15—B16—B7^vii^	65.0 (5)
B8—B6—B7—B16^iv^	−106.1 (5)	B17—B15—B16—B19	42.3 (3)
B2—B6—B7—B16^iv^	−143.6 (3)	B14—B15—B16—B19	−67.6 (4)
B11^iv^—B6—B7—B16^iv^	22.1 (5)	B13—B15—B16—B19	−103.4 (4)
B4—B6—B7—B16^iv^	150.1 (3)	B18—B15—B16—B19	5.5 (5)
B5—B6—B7—B16^iv^	119.4 (4)	B3^ii^—B15—B16—B19	146.5 (3)
Mg2—B6—B7—B16^iv^	87.4 (4)	Li1^ii^—B15—B16—B19	−145.7 (3)
B8—B6—B7—B5	134.5 (4)	Mg1^ii^—B15—B16—B19	−145.7 (3)
B2—B6—B7—B5	97.0 (3)	B17—B15—B16—B13	145.7 (4)
B11^iv^—B6—B7—B5	−97.3 (4)	B14—B15—B16—B13	35.8 (3)
B4—B6—B7—B5	30.7 (3)	B18—B15—B16—B13	108.9 (4)
Mg2—B6—B7—B5	−32.0 (2)	B3^ii^—B15—B16—B13	−110.1 (4)
B2—B6—B7—B8	−37.5 (4)	Li1^ii^—B15—B16—B13	−42.3 (3)
B11^iv^—B6—B7—B8	128.2 (5)	Mg1^ii^—B15—B16—B13	−42.3 (3)
B4—B6—B7—B8	−103.8 (4)	B17—B15—B16—B22	102.6 (3)
B5—B6—B7—B8	−134.5 (4)	B14—B15—B16—B22	−7.3 (4)
Mg2—B6—B7—B8	−166.5 (4)	B13—B15—B16—B22	−43.1 (3)
B8—B6—B7—B9	36.6 (4)	B18—B15—B16—B22	65.8 (4)
B2—B6—B7—B9	−0.9 (4)	B3^ii^—B15—B16—B22	−153.2 (3)
B11^iv^—B6—B7—B9	164.8 (3)	Li1^ii^—B15—B16—B22	−85.4 (3)
B4—B6—B7—B9	−67.2 (4)	Mg1^ii^—B15—B16—B22	−85.4 (3)
B5—B6—B7—B9	−98.0 (3)	B14—B15—B16—B17	−110.0 (4)
Mg2—B6—B7—B9	−129.9 (3)	B13—B15—B16—B17	−145.7 (4)
B8—B6—B7—B1	100.7 (4)	B18—B15—B16—B17	−36.9 (3)
B2—B6—B7—B1	63.2 (4)	B3^ii^—B15—B16—B17	104.2 (4)
B11^iv^—B6—B7—B1	−131.1 (3)	Li1^ii^—B15—B16—B17	172.0 (4)
B4—B6—B7—B1	−3.1 (4)	Mg1^ii^—B15—B16—B17	172.0 (4)
B5—B6—B7—B1	−33.8 (3)	B14—B15—B17—B24	−156.5 (4)
Mg2—B6—B7—B1	−65.8 (3)	B13—B15—B17—B24	130.7 (4)
B5—B1—B7—B16^iv^	−115.0 (4)	B18—B15—B17—B24	−121.6 (4)
B3—B1—B7—B16^iv^	−151.0 (4)	B16—B15—B17—B24	96.1 (3)
B11—B1—B7—B16^iv^	151.3 (4)	B3^ii^—B15—B17—B24	−3.6 (4)
B25^i^—B1—B7—B16^iv^	−18.5 (5)	B14—B15—B17—B18	−34.8 (4)
B9—B1—B7—B16^iv^	111.8 (4)	B13—B15—B17—B18	−107.7 (4)
B24^i^—B1—B7—B16^iv^	−38.5 (10)	B16—B15—B17—B18	−142.3 (3)
B3—B1—B7—B5	−36.0 (3)	B3^ii^—B15—B17—B18	118.0 (4)
B11—B1—B7—B5	−93.7 (3)	B14—B15—B17—B16	107.4 (4)
B25^i^—B1—B7—B5	96.5 (3)	B13—B15—B17—B16	34.6 (4)
B9—B1—B7—B5	−133.2 (3)	B18—B15—B17—B16	142.3 (3)
B24^i^—B1—B7—B5	76.5 (8)	B3^ii^—B15—B17—B16	−99.7 (3)
B5—B1—B7—B6	35.9 (3)	B14—B15—B17—B19	67.9 (6)
B3—B1—B7—B6	0.0 (4)	B13—B15—B17—B19	−5.0 (6)
B11—B1—B7—B6	−57.8 (4)	B18—B15—B17—B19	102.8 (5)
B25^i^—B1—B7—B6	132.5 (3)	B16—B15—B17—B19	−39.5 (5)
B9—B1—B7—B6	−97.3 (3)	B3^ii^—B15—B17—B19	−139.2 (5)
B24^i^—B1—B7—B6	112.4 (8)	B14—B15—B17—B20^viii^	9.0 (5)
B5—B1—B7—B8	96.2 (4)	B13—B15—B17—B20^viii^	−63.9 (5)
B3—B1—B7—B8	60.2 (4)	B18—B15—B17—B20^viii^	43.8 (3)
B11—B1—B7—B8	2.4 (4)	B16—B15—B17—B20^viii^	−98.5 (3)
B25^i^—B1—B7—B8	−167.3 (4)	B3^ii^—B15—B17—B20^viii^	161.8 (3)
B9—B1—B7—B8	−37.1 (3)	B24—B17—B18—B15	80.4 (4)
B24^i^—B1—B7—B8	172.6 (8)	B16—B17—B18—B15	−36.3 (3)
B5—B1—B7—B9	133.2 (3)	B19—B17—B18—B15	−96.2 (3)
B3—B1—B7—B9	97.3 (4)	B20^viii^—B17—B18—B15	−129.3 (3)
B11—B1—B7—B9	39.5 (3)	B15—B17—B18—B8^ix^	−112.3 (4)
B25^i^—B1—B7—B9	−130.2 (4)	B24—B17—B18—B8^ix^	−31.9 (6)
B24^i^—B1—B7—B9	−150.3 (9)	B16—B17—B18—B8^ix^	−148.6 (4)
B2—B6—B8—B18^v^	−111.2 (5)	B19—B17—B18—B8^ix^	151.5 (4)
B11^iv^—B6—B8—B18^v^	6.5 (8)	B20^viii^—B17—B18—B8^ix^	118.4 (5)
B4—B6—B8—B18^v^	−151.1 (4)	B15—B17—B18—B14	28.5 (3)
B5—B6—B8—B18^v^	146.2 (4)	B24—B17—B18—B14	108.9 (4)
B7—B6—B8—B18^v^	108.5 (5)	B16—B17—B18—B14	−7.8 (4)
Mg2—B6—B8—B18^v^	144.7 (8)	B19—B17—B18—B14	−67.7 (4)
B2—B6—B8—B10	37.4 (3)	B20^viii^—B17—B18—B14	−100.8 (3)
B11^iv^—B6—B8—B10	155.1 (4)	B15—B17—B18—B21	90.1 (4)
B4—B6—B8—B10	−2.4 (5)	B24—B17—B18—B21	170.4 (4)
B5—B6—B8—B10	−65.2 (4)	B16—B17—B18—B21	53.8 (5)
B7—B6—B8—B10	−102.9 (4)	B19—B17—B18—B21	−6.1 (4)
Mg2—B6—B8—B10	−66.7 (10)	B20^viii^—B17—B18—B21	−39.2 (3)
B2—B6—B8—B9	102.0 (4)	B15—B17—B18—B20^viii^	129.3 (3)
B11^iv^—B6—B8—B9	−140.2 (5)	B24—B17—B18—B20^viii^	−150.3 (4)
B4—B6—B8—B9	62.2 (5)	B16—B17—B18—B20^viii^	93.0 (4)
B5—B6—B8—B9	−0.5 (5)	B19—B17—B18—B20^viii^	33.1 (3)
B7—B6—B8—B9	−38.3 (4)	B14—B15—B18—B17	145.8 (4)
Mg2—B6—B8—B9	−2.0 (12)	B13—B15—B18—B17	109.3 (4)
B11^iv^—B6—B8—B2	117.7 (6)	B16—B15—B18—B17	38.5 (3)
B4—B6—B8—B2	−39.8 (3)	B3^ii^—B15—B18—B17	−95.4 (4)
B5—B6—B8—B2	−102.5 (3)	Li1^ii^—B15—B18—B17	−172.8 (4)
B7—B6—B8—B2	−140.3 (4)	Mg1^ii^—B15—B18—B17	−172.8 (4)
Mg2—B6—B8—B2	−104.1 (9)	B17—B15—B18—B8^ix^	96.6 (5)
B2—B6—B8—B7	140.3 (4)	B14—B15—B18—B8^ix^	−117.5 (5)
B11^iv^—B6—B8—B7	−102.0 (5)	B13—B15—B18—B8^ix^	−154.0 (4)
B4—B6—B8—B7	100.5 (4)	B16—B15—B18—B8^ix^	135.1 (4)
B5—B6—B8—B7	37.7 (3)	B3^ii^—B15—B18—B8^ix^	1.3 (7)
Mg2—B6—B8—B7	36.2 (10)	Li1^ii^—B15—B18—B8^ix^	−76.2 (6)
B10—B2—B8—B6	136.6 (4)	Mg1^ii^—B15—B18—B8^ix^	−76.2 (6)
B4—B2—B8—B6	37.7 (3)	B17—B15—B18—B14	−145.8 (4)
B12—B2—B8—B6	97.0 (4)	B13—B15—B18—B14	−36.5 (4)
B23^ii^—B2—B8—B6	−119.4 (5)	B16—B15—B18—B14	−107.3 (4)
Li1^ii^—B2—B8—B6	164.3 (4)	B3^ii^—B15—B18—B14	118.8 (4)
Mg1^ii^—B2—B8—B6	164.3 (4)	Li1^ii^—B15—B18—B14	41.4 (3)
B10—B2—B8—B18^v^	−124.0 (5)	Mg1^ii^—B15—B18—B14	41.4 (3)
B6—B2—B8—B18^v^	99.4 (5)	B17—B15—B18—B21	−107.3 (4)
B4—B2—B8—B18^v^	137.1 (5)	B14—B15—B18—B21	38.6 (3)
B12—B2—B8—B18^v^	−163.6 (4)	B13—B15—B18—B21	2.1 (5)
B23^ii^—B2—B8—B18^v^	−20.1 (7)	B16—B15—B18—B21	−68.8 (4)
Li1^ii^—B2—B8—B18^v^	−96.4 (5)	B3^ii^—B15—B18—B21	157.4 (3)
Mg1^ii^—B2—B8—B18^v^	−96.4 (5)	Li1^ii^—B15—B18—B21	79.9 (4)
B6—B2—B8—B10	−136.6 (4)	Mg1^ii^—B15—B18—B21	79.9 (4)
B4—B2—B8—B10	−98.9 (3)	B17—B15—B18—B20^viii^	−46.9 (3)
B12—B2—B8—B10	−39.7 (3)	B14—B15—B18—B20^viii^	98.9 (3)
B23^ii^—B2—B8—B10	103.9 (4)	B13—B15—B18—B20^viii^	62.4 (5)
Li1^ii^—B2—B8—B10	27.6 (4)	B16—B15—B18—B20^viii^	−8.4 (5)
Mg1^ii^—B2—B8—B10	27.6 (4)	B3^ii^—B15—B18—B20^viii^	−142.3 (3)
B10—B2—B8—B9	35.0 (3)	Li1^ii^—B15—B18—B20^viii^	140.3 (3)
B6—B2—B8—B9	−101.7 (5)	Mg1^ii^—B15—B18—B20^viii^	140.3 (3)
B4—B2—B8—B9	−63.9 (5)	B15—B14—B18—B17	−29.0 (3)
B12—B2—B8—B9	−4.7 (5)	B13—B14—B18—B17	3.4 (4)
B23^ii^—B2—B8—B9	138.9 (4)	B21—B14—B18—B17	105.2 (4)
Li1^ii^—B2—B8—B9	62.6 (5)	B22^ii^—B14—B18—B17	−133.2 (4)
Mg1^ii^—B2—B8—B9	62.6 (5)	B23—B14—B18—B17	68.6 (4)
B10—B2—B8—B7	99.9 (4)	Li1^ii^—B14—B18—B17	−65.2 (4)
B6—B2—B8—B7	−36.8 (4)	Mg1^ii^—B14—B18—B17	−65.2 (4)
B4—B2—B8—B7	1.0 (5)	B13—B14—B18—B15	32.5 (3)
B12—B2—B8—B7	60.2 (5)	B21—B14—B18—B15	134.2 (4)
B23^ii^—B2—B8—B7	−156.2 (4)	B22^ii^—B14—B18—B15	−104.2 (4)
Li1^ii^—B2—B8—B7	127.5 (4)	B23—B14—B18—B15	97.6 (4)
Mg1^ii^—B2—B8—B7	127.5 (4)	Li1^ii^—B14—B18—B15	−36.2 (3)
B16^iv^—B7—B8—B6	108.4 (4)	Mg1^ii^—B14—B18—B15	−36.2 (3)
B5—B7—B8—B6	−40.6 (3)	B15—B14—B18—B8^ix^	102.7 (5)
B9—B7—B8—B6	−137.9 (4)	B13—B14—B18—B8^ix^	135.1 (5)
B1—B7—B8—B6	−99.9 (4)	B21—B14—B18—B8^ix^	−123.1 (5)
B16^iv^—B7—B8—B18^v^	7.2 (7)	B22^ii^—B14—B18—B8^ix^	−1.5 (7)
B5—B7—B8—B18^v^	−141.7 (4)	B23—B14—B18—B8^ix^	−159.7 (4)
B6—B7—B8—B18^v^	−101.2 (5)	Li1^ii^—B14—B18—B8^ix^	66.5 (6)
B9—B7—B8—B18^v^	120.9 (5)	Mg1^ii^—B14—B18—B8^ix^	66.5 (6)
B1—B7—B8—B18^v^	158.9 (4)	B15—B14—B18—B21	−134.2 (4)
B16^iv^—B7—B8—B10	−153.2 (4)	B13—B14—B18—B21	−101.8 (4)
B5—B7—B8—B10	57.8 (5)	B22^ii^—B14—B18—B21	121.6 (4)
B6—B7—B8—B10	98.4 (4)	B23—B14—B18—B21	−36.7 (3)
B9—B7—B8—B10	−39.5 (3)	Li1^ii^—B14—B18—B21	−170.4 (4)
B1—B7—B8—B10	−1.5 (5)	Mg1^ii^—B14—B18—B21	−170.4 (4)
B16^iv^—B7—B8—B9	−113.7 (4)	B15—B14—B18—B20^viii^	−97.5 (4)
B5—B7—B8—B9	97.3 (3)	B13—B14—B18—B20^viii^	−65.1 (4)
B6—B7—B8—B9	137.9 (4)	B21—B14—B18—B20^viii^	36.7 (3)
B1—B7—B8—B9	38.0 (3)	B22^ii^—B14—B18—B20^viii^	158.3 (3)
B16^iv^—B7—B8—B2	144.6 (4)	B23—B14—B18—B20^viii^	0.0 (5)
B5—B7—B8—B2	−4.4 (5)	Li1^ii^—B14—B18—B20^viii^	−133.7 (3)
B6—B7—B8—B2	36.2 (3)	Mg1^ii^—B14—B18—B20^viii^	−133.7 (3)
B9—B7—B8—B2	−101.7 (5)	B7^vii^—B16—B19—B16^viii^	−0.5 (3)
B1—B7—B8—B2	−63.7 (5)	B13—B16—B19—B16^viii^	157.1 (3)
B6—B8—B9—B9^vi^	162.1 (3)	B22—B16—B19—B16^viii^	119.4 (3)
B18^v^—B8—B9—B9^vi^	22.6 (7)	B15—B16—B19—B16^viii^	−147.9 (3)
B10—B8—B9—B9^vi^	−100.0 (3)	B17—B16—B19—B16^viii^	−111.5 (3)
B2—B8—B9—B9^vi^	−133.7 (3)	B7^vii^—B16—B19—B22	−119.9 (5)
B7—B8—B9—B9^vi^	123.4 (3)	B13—B16—B19—B22	37.7 (4)
B6—B8—B9—B7	38.6 (4)	B15—B16—B19—B22	92.7 (3)
B18^v^—B8—B9—B7	−100.8 (6)	B17—B16—B19—B22	129.1 (5)
B10—B8—B9—B7	136.6 (4)	B7^vii^—B16—B19—B22^viii^	88.0 (9)
B2—B8—B9—B7	102.8 (5)	B13—B16—B19—B22^viii^	−114.3 (8)
B6—B8—B9—B10	−98.0 (4)	B22—B16—B19—B22^viii^	−152.1 (11)
B18^v^—B8—B9—B10	122.6 (6)	B15—B16—B19—B22^viii^	−59.4 (9)
B2—B8—B9—B10	−33.8 (3)	B17—B16—B19—B22^viii^	−23.0 (7)
B7—B8—B9—B10	−136.6 (4)	B7^vii^—B16—B19—B20^viii^	147.4 (5)
B6—B8—B9—B11	−59.3 (5)	B13—B16—B19—B20^viii^	−55.0 (4)
B18^v^—B8—B9—B11	161.3 (5)	B22—B16—B19—B20^viii^	−92.7 (5)
B10—B8—B9—B11	38.7 (3)	B15—B16—B19—B20^viii^	−0.1 (5)
B2—B8—B9—B11	5.0 (5)	B17—B16—B19—B20^viii^	36.4 (3)
B7—B8—B9—B11	−97.9 (3)	B7^vii^—B16—B19—B20	−149.7 (5)
B6—B8—B9—B1	1.0 (5)	B13—B16—B19—B20	7.9 (4)
B18^v^—B8—B9—B1	−138.4 (6)	B22—B16—B19—B20	−29.8 (3)
B10—B8—B9—B1	99.0 (4)	B15—B16—B19—B20	62.9 (3)
B2—B8—B9—B1	65.3 (5)	B17—B16—B19—B20	99.3 (5)
B7—B8—B9—B1	−37.6 (3)	B7^vii^—B16—B19—B17^viii^	−83.4 (8)
B6—B8—B9—Li2^ii^	−121.4 (4)	B13—B16—B19—B17^viii^	74.2 (8)
B18^v^—B8—B9—Li2^ii^	99.1 (6)	B22—B16—B19—B17^viii^	36.5 (6)
B10—B8—B9—Li2^ii^	−23.4 (3)	B15—B16—B19—B17^viii^	129.1 (7)
B2—B8—B9—Li2^ii^	−57.2 (5)	B17—B16—B19—B17^viii^	165.6 (9)
B7—B8—B9—Li2^ii^	−160.0 (3)	B7^vii^—B16—B19—B17	111.0 (5)
B6—B8—B9—Mg2^ii^	−121.4 (4)	B13—B16—B19—B17	−91.4 (3)
B18^v^—B8—B9—Mg2^ii^	99.1 (6)	B22—B16—B19—B17	−129.1 (5)
B10—B8—B9—Mg2^ii^	−23.4 (3)	B15—B16—B19—B17	−36.5 (3)
B2—B8—B9—Mg2^ii^	−57.2 (5)	B16^viii^—B19—B20—B22	−124.3 (4)
B7—B8—B9—Mg2^ii^	−160.0 (3)	B16—B19—B20—B22	33.2 (3)
B16^iv^—B7—B9—B8	105.4 (5)	B22^viii^—B19—B20—B22	171.7 (2)
B5—B7—B9—B8	−99.5 (4)	B20^viii^—B19—B20—B22	140.0 (4)
B6—B7—B9—B8	−35.5 (3)	B17^viii^—B19—B20—B22	−88.1 (3)
B1—B7—B9—B8	−138.3 (4)	B17—B19—B20—B22	97.1 (3)
B16^iv^—B7—B9—B9^vi^	0.8 (7)	B16^viii^—B19—B20—B23	−159.2 (3)
B5—B7—B9—B9^vi^	155.9 (5)	B16—B19—B20—B23	−1.7 (5)
B6—B7—B9—B9^vi^	−140.2 (5)	B22—B19—B20—B23	−34.9 (3)
B8—B7—B9—B9^vi^	−104.7 (6)	B22^viii^—B19—B20—B23	136.8 (3)
B1—B7—B9—B9^vi^	117.0 (6)	B20^viii^—B19—B20—B23	105.1 (4)
B16^iv^—B7—B9—B10	143.2 (4)	B17^viii^—B19—B20—B23	−123.0 (3)
B5—B7—B9—B10	−61.7 (3)	B17—B19—B20—B23	62.2 (4)
B6—B7—B9—B10	2.3 (4)	B16^viii^—B19—B20—B21^viii^	58.0 (5)
B8—B7—B9—B10	37.8 (3)	B16—B19—B20—B21^viii^	−144.5 (4)
B1—B7—B9—B10	−100.5 (3)	B22—B19—B20—B21^viii^	−177.7 (4)
B16^iv^—B7—B9—B11	−154.6 (4)	B22^viii^—B19—B20—B21^viii^	−6.0 (4)
B5—B7—B9—B11	0.5 (4)	B20^viii^—B19—B20—B21^viii^	−37.7 (2)
B6—B7—B9—B11	64.5 (3)	B17^viii^—B19—B20—B21^viii^	94.2 (3)
B8—B7—B9—B11	100.0 (4)	B17—B19—B20—B21^viii^	−80.6 (3)
B1—B7—B9—B11	−38.3 (3)	B16^viii^—B19—B20—B21	132.8 (4)
B16^iv^—B7—B9—B1	−116.3 (4)	B16—B19—B20—B21	−69.7 (4)
B5—B7—B9—B1	38.8 (3)	B22—B19—B20—B21	−102.9 (4)
B6—B7—B9—B1	102.8 (3)	B22^viii^—B19—B20—B21	68.8 (3)
B8—B7—B9—B1	138.3 (4)	B20^viii^—B19—B20—B21	37.1 (2)
B16^iv^—B7—B9—Li2^ii^	178.5 (7)	B17^viii^—B19—B20—B21	169.1 (4)
B5—B7—B9—Li2^ii^	−26.4 (10)	B17—B19—B20—B21	−5.7 (4)
B6—B7—B9—Li2^ii^	37.5 (10)	B16^viii^—B19—B20—B20^viii^	95.7 (4)
B8—B7—B9—Li2^ii^	73.0 (9)	B16—B19—B20—B20^viii^	−106.8 (4)
B1—B7—B9—Li2^ii^	−65.3 (8)	B22—B19—B20—B20^viii^	−140.0 (4)
B16^iv^—B7—B9—Mg2^ii^	178.5 (7)	B22^viii^—B19—B20—B20^viii^	31.7 (3)
B5—B7—B9—Mg2^ii^	−26.4 (10)	B17^viii^—B19—B20—B20^viii^	131.9 (4)
B6—B7—B9—Mg2^ii^	37.5 (10)	B17—B19—B20—B20^viii^	−42.9 (4)
B8—B7—B9—Mg2^ii^	73.0 (9)	B16^viii^—B19—B20—B18^viii^	−4.7 (5)
B1—B7—B9—Mg2^ii^	−65.3 (8)	B16—B19—B20—B18^viii^	152.8 (3)
B5—B1—B9—B8	−1.1 (4)	B22—B19—B20—B18^viii^	119.6 (4)
B3—B1—B9—B8	−64.0 (4)	B22^viii^—B19—B20—B18^viii^	−68.7 (4)
B11—B1—B9—B8	−97.0 (4)	B20^viii^—B19—B20—B18^viii^	−100.4 (4)
B25^i^—B1—B9—B8	125.6 (5)	B17^viii^—B19—B20—B18^viii^	31.5 (3)
B7—B1—B9—B8	37.5 (3)	B17—B19—B20—B18^viii^	−143.3 (3)
B24^i^—B1—B9—B8	−160.6 (5)	B16^viii^—B19—B20—B17^viii^	−36.2 (3)
B5—B1—B9—B9^vi^	−159.92 (19)	B16—B19—B20—B17^viii^	121.3 (4)
B3—B1—B9—B9^vi^	137.2 (2)	B22—B19—B20—B17^viii^	88.1 (3)
B11—B1—B9—B9^vi^	104.2 (3)	B22^viii^—B19—B20—B17^viii^	−100.3 (2)
B25^i^—B1—B9—B9^vi^	−33.2 (6)	B20^viii^—B19—B20—B17^viii^	−131.9 (4)
B7—B1—B9—B9^vi^	−121.3 (3)	B17—B19—B20—B17^viii^	−174.8 (3)
B24^i^—B1—B9—B9^vi^	40.6 (6)	B15—B14—B21—B24^x^	−178.2 (3)
B5—B1—B9—B7	−38.6 (3)	B13—B14—B21—B24^x^	−116.2 (4)
B3—B1—B9—B7	−101.4 (3)	B18—B14—B21—B24^x^	141.5 (4)
B11—B1—B9—B7	−134.5 (3)	B22^ii^—B14—B21—B24^x^	40.7 (5)
B25^i^—B1—B9—B7	88.1 (5)	B23—B14—B21—B24^x^	−78.5 (3)
B24^i^—B1—B9—B7	161.9 (6)	B15—B14—B21—B25^xi^	156.4 (5)
B5—B1—B9—B10	61.0 (3)	B13—B14—B21—B25^xi^	−141.5 (5)
B3—B1—B9—B10	−1.9 (4)	B18—B14—B21—B25^xi^	116.2 (6)
B11—B1—B9—B10	−35.0 (3)	B22^ii^—B14—B21—B25^xi^	15.3 (9)
B25^i^—B1—B9—B10	−172.3 (4)	B23—B14—B21—B25^xi^	−103.8 (6)
B7—B1—B9—B10	99.6 (3)	B15—B14—B21—B20^viii^	1.4 (4)
B24^i^—B1—B9—B10	−98.5 (5)	B13—B14—B21—B20^viii^	63.5 (4)
B5—B1—B9—B11	95.9 (3)	B18—B14—B21—B20^viii^	−38.8 (3)
B3—B1—B9—B11	33.1 (3)	B22^ii^—B14—B21—B20^viii^	−139.7 (4)
B25^i^—B1—B9—B11	−137.3 (5)	B23—B14—B21—B20^viii^	101.2 (3)
B7—B1—B9—B11	134.5 (3)	B15—B14—B21—B20	−63.0 (4)
B24^i^—B1—B9—B11	−63.6 (5)	B13—B14—B21—B20	−0.9 (4)
B5—B1—B9—Li2^ii^	122.8 (3)	B18—B14—B21—B20	−103.3 (4)
B3—B1—B9—Li2^ii^	60.0 (4)	B22^ii^—B14—B21—B20	155.9 (4)
B11—B1—B9—Li2^ii^	26.9 (3)	B23—B14—B21—B20	36.8 (3)
B25^i^—B1—B9—Li2^ii^	−110.5 (4)	B15—B14—B21—B18	40.2 (3)
B7—B1—B9—Li2^ii^	161.4 (3)	B13—B14—B21—B18	102.3 (4)
B24^i^—B1—B9—Li2^ii^	−36.7 (6)	B22^ii^—B14—B21—B18	−100.8 (4)
B5—B1—B9—Mg2^ii^	122.8 (3)	B23—B14—B21—B18	140.0 (3)
B3—B1—B9—Mg2^ii^	60.0 (4)	B15—B14—B21—B23	−99.8 (4)
B11—B1—B9—Mg2^ii^	26.9 (3)	B13—B14—B21—B23	−37.7 (3)
B25^i^—B1—B9—Mg2^ii^	−110.5 (4)	B18—B14—B21—B23	−140.0 (3)
B7—B1—B9—Mg2^ii^	161.4 (3)	B22^ii^—B14—B21—B23	119.1 (4)
B24^i^—B1—B9—Mg2^ii^	−36.7 (6)	B15—B14—B21—B21^viii^	−39.6 (12)
B6—B2—B10—B12	−95.6 (3)	B13—B14—B21—B21^viii^	22.5 (12)
B8—B2—B10—B12	−132.8 (4)	B18—B14—B21—B21^viii^	−79.9 (11)
B4—B2—B10—B12	−33.2 (2)	B22^ii^—B14—B21—B21^viii^	179.3 (9)
B23^ii^—B2—B10—B12	109.4 (4)	B23—B14—B21—B21^viii^	60.2 (11)
Li1^ii^—B2—B10—B12	72.7 (2)	B22—B20—B21—B24^x^	104.6 (4)
Mg1^ii^—B2—B10—B12	72.7 (2)	B23—B20—B21—B24^x^	64.4 (3)
B6—B2—B10—B8	37.2 (4)	B21^viii^—B20—B21—B24^x^	−86.0 (3)
B4—B2—B10—B8	99.6 (4)	B20^viii^—B20—B21—B24^x^	−151.6 (4)
B12—B2—B10—B8	132.8 (4)	B18^viii^—B20—B21—B24^x^	−63.5 (5)
B23^ii^—B2—B10—B8	−117.8 (4)	B19—B20—B21—B24^x^	170.7 (3)
Li1^ii^—B2—B10—B8	−154.5 (3)	B17^viii^—B20—B21—B24^x^	−150.7 (12)
Mg1^ii^—B2—B10—B8	−154.5 (3)	B22—B20—B21—B14	−0.1 (4)
B6—B2—B10—B4^ii^	155.3 (3)	B23—B20—B21—B14	−40.3 (3)
B8—B2—B10—B4^ii^	118.1 (4)	B21^viii^—B20—B21—B14	169.4 (4)
B4—B2—B10—B4^ii^	−142.2 (2)	B20^viii^—B20—B21—B14	103.7 (4)
B12—B2—B10—B4^ii^	−109.1 (3)	B18^viii^—B20—B21—B14	−168.2 (4)
B23^ii^—B2—B10—B4^ii^	0.3 (4)	B19—B20—B21—B14	66.0 (4)
Li1^ii^—B2—B10—B4^ii^	−36.4 (2)	B17^viii^—B20—B21—B14	104.6 (13)
Mg1^ii^—B2—B10—B4^ii^	−36.4 (2)	B22—B20—B21—B25^xi^	159.7 (3)
B6—B2—B10—B11	−61.9 (3)	B23—B20—B21—B25^xi^	119.5 (3)
B8—B2—B10—B11	−99.1 (3)	B21^viii^—B20—B21—B25^xi^	−30.8 (2)
B4—B2—B10—B11	0.5 (3)	B20^viii^—B20—B21—B25^xi^	−96.5 (3)
B12—B2—B10—B11	33.7 (3)	B18^viii^—B20—B21—B25^xi^	−8.3 (5)
B23^ii^—B2—B10—B11	143.1 (3)	B19—B20—B21—B25^xi^	−134.2 (3)
Li1^ii^—B2—B10—B11	106.4 (2)	B17^viii^—B20—B21—B25^xi^	−95.6 (13)
Mg1^ii^—B2—B10—B11	106.4 (2)	B22—B20—B21—B20^viii^	−103.8 (3)
B6—B2—B10—B9	2.5 (4)	B23—B20—B21—B20^viii^	−144.0 (3)
B8—B2—B10—B9	−34.7 (4)	B21^viii^—B20—B21—B20^viii^	65.7 (3)
B4—B2—B10—B9	64.9 (3)	B18^viii^—B20—B21—B20^viii^	88.2 (5)
B12—B2—B10—B9	98.1 (3)	B19—B20—B21—B20^viii^	−37.7 (2)
B23^ii^—B2—B10—B9	−152.5 (3)	B17^viii^—B20—B21—B20^viii^	0.9 (13)
Li1^ii^—B2—B10—B9	170.8 (3)	B22—B20—B21—B18	−63.7 (4)
Mg1^ii^—B2—B10—B9	170.8 (3)	B23—B20—B21—B18	−103.9 (4)
B6—B2—B10—Li1^ii^	−168.3 (3)	B21^viii^—B20—B21—B18	105.7 (4)
B8—B2—B10—Li1^ii^	154.5 (3)	B20^viii^—B20—B21—B18	40.1 (3)
B4—B2—B10—Li1^ii^	−105.8 (3)	B18^viii^—B20—B21—B18	128.2 (3)
B12—B2—B10—Li1^ii^	−72.7 (2)	B19—B20—B21—B18	2.3 (4)
B23^ii^—B2—B10—Li1^ii^	36.7 (3)	B17^viii^—B20—B21—B18	41.0 (15)
Mg1^ii^—B2—B10—Li1^ii^	0.000 (1)	B22—B20—B21—B23	40.2 (3)
B6—B2—B10—Mg1^ii^	−168.3 (3)	B21^viii^—B20—B21—B23	−150.3 (3)
B8—B2—B10—Mg1^ii^	154.5 (3)	B20^viii^—B20—B21—B23	144.0 (3)
B4—B2—B10—Mg1^ii^	−105.8 (3)	B18^viii^—B20—B21—B23	−127.8 (5)
B12—B2—B10—Mg1^ii^	−72.7 (2)	B19—B20—B21—B23	106.3 (3)
B23^ii^—B2—B10—Mg1^ii^	36.7 (3)	B17^viii^—B20—B21—B23	144.9 (14)
Li1^ii^—B2—B10—Mg1^ii^	0.000 (1)	B22—B20—B21—B21^viii^	−169.5 (4)
B6—B2—B10—Li2^ii^	−117.2 (12)	B23—B20—B21—B21^viii^	150.3 (3)
B8—B2—B10—Li2^ii^	−154.4 (13)	B20^viii^—B20—B21—B21^viii^	−65.7 (3)
B4—B2—B10—Li2^ii^	−54.8 (14)	B18^viii^—B20—B21—B21^viii^	22.5 (4)
B12—B2—B10—Li2^ii^	−21.6 (13)	B19—B20—B21—B21^viii^	−103.4 (3)
B23^ii^—B2—B10—Li2^ii^	87.8 (13)	B17^viii^—B20—B21—B21^viii^	−64.7 (13)
Li1^ii^—B2—B10—Li2^ii^	51.1 (13)	B17—B18—B21—B24^x^	−161.3 (4)
Mg1^ii^—B2—B10—Li2^ii^	51.1 (13)	B15—B18—B21—B24^x^	−103.0 (5)
B6—B2—B10—Mg2^ii^	−117.2 (12)	B8^ix^—B18—B21—B24^x^	48.0 (8)
B8—B2—B10—Mg2^ii^	−154.4 (13)	B14—B18—B21—B24^x^	−66.1 (5)
B4—B2—B10—Mg2^ii^	−54.8 (14)	B20^viii^—B18—B21—B24^x^	156.2 (6)
B12—B2—B10—Mg2^ii^	−21.6 (13)	B17—B18—B21—B14	−95.2 (4)
B23^ii^—B2—B10—Mg2^ii^	87.8 (13)	B15—B18—B21—B14	−36.9 (3)
Li1^ii^—B2—B10—Mg2^ii^	51.1 (13)	B8^ix^—B18—B21—B14	114.1 (6)
Mg1^ii^—B2—B10—Mg2^ii^	51.1 (13)	B20^viii^—B18—B21—B14	−137.7 (4)
B6—B8—B10—B12	6.0 (5)	B17—B18—B21—B25^xi^	122.6 (4)
B18^v^—B8—B10—B12	145.3 (5)	B15—B18—B21—B25^xi^	−179.0 (4)
B9—B8—B10—B12	−97.8 (3)	B8^ix^—B18—B21—B25^xi^	−28.1 (8)
B2—B8—B10—B12	44.2 (3)	B14—B18—B21—B25^xi^	−142.2 (5)
B7—B8—B10—B12	−58.7 (4)	B20^viii^—B18—B21—B25^xi^	80.2 (4)
B6—B8—B10—B2	−38.2 (3)	B17—B18—B21—B20^viii^	42.4 (3)
B18^v^—B8—B10—B2	101.1 (6)	B15—B18—B21—B20^viii^	100.8 (4)
B9—B8—B10—B2	−142.0 (4)	B8^ix^—B18—B21—B20^viii^	−108.3 (6)
B7—B8—B10—B2	−102.9 (4)	B14—B18—B21—B20^viii^	137.7 (4)
B6—B8—B10—B4^ii^	−142.4 (3)	B17—B18—B21—B20	2.5 (5)
B18^v^—B8—B10—B4^ii^	−3.1 (8)	B15—B18—B21—B20	60.9 (4)
B9—B8—B10—B4^ii^	113.8 (3)	B8^ix^—B18—B21—B20	−148.2 (5)
B2—B8—B10—B4^ii^	−104.2 (3)	B14—B18—B21—B20	97.7 (4)
B7—B8—B10—B4^ii^	153.0 (3)	B20^viii^—B18—B21—B20	−39.9 (3)
B6—B8—B10—B11	64.6 (4)	B17—B18—B21—B23	−57.1 (4)
B18^v^—B8—B10—B11	−156.1 (5)	B15—B18—B21—B23	1.3 (4)
B9—B8—B10—B11	−39.2 (3)	B8^ix^—B18—B21—B23	152.2 (5)
B2—B8—B10—B11	102.9 (3)	B14—B18—B21—B23	38.1 (3)
B7—B8—B10—B11	0.0 (5)	B20^viii^—B18—B21—B23	−99.5 (3)
B6—B8—B10—B9	103.8 (5)	B17—B18—B21—B21^viii^	60.9 (4)
B18^v^—B8—B10—B9	−116.9 (7)	B15—B18—B21—B21^viii^	119.2 (3)
B2—B8—B10—B9	142.0 (4)	B8^ix^—B18—B21—B21^viii^	−89.8 (6)
B7—B8—B10—B9	39.2 (3)	B14—B18—B21—B21^viii^	156.1 (3)
B6—B8—B10—Li1^ii^	−64.7 (5)	B20^viii^—B18—B21—B21^viii^	18.5 (3)
B18^v^—B8—B10—Li1^ii^	74.6 (6)	B21—B20—B22—B23	−42.1 (3)
B9—B8—B10—Li1^ii^	−168.5 (3)	B20^viii^—B20—B22—B23	−105.3 (4)
B2—B8—B10—Li1^ii^	−26.5 (4)	B18^viii^—B20—B22—B23	126.4 (4)
B7—B8—B10—Li1^ii^	−129.4 (3)	B19—B20—B22—B23	−142.0 (3)
B6—B8—B10—Mg1^ii^	−64.7 (5)	B17^viii^—B20—B22—B23	153.3 (3)
B18^v^—B8—B10—Mg1^ii^	74.6 (6)	B23—B20—B22—B19	142.0 (3)
B9—B8—B10—Mg1^ii^	−168.5 (3)	B21—B20—B22—B19	99.9 (3)
B2—B8—B10—Mg1^ii^	−26.5 (4)	B20^viii^—B20—B22—B19	36.7 (3)
B7—B8—B10—Mg1^ii^	−129.4 (3)	B18^viii^—B20—B22—B19	−91.5 (4)
B6—B8—B10—Li2^ii^	135.8 (3)	B17^viii^—B20—B22—B19	−64.7 (2)
B18^v^—B8—B10—Li2^ii^	−84.9 (6)	B23—B20—B22—B13	43.2 (3)
B9—B8—B10—Li2^ii^	32.0 (4)	B21—B20—B22—B13	1.1 (4)
B2—B8—B10—Li2^ii^	174.0 (3)	B20^viii^—B20—B22—B13	−62.1 (4)
B7—B8—B10—Li2^ii^	71.1 (5)	B18^viii^—B20—B22—B13	169.6 (4)
B6—B8—B10—Mg2^ii^	135.8 (3)	B19—B20—B22—B13	−98.8 (3)
B18^v^—B8—B10—Mg2^ii^	−84.9 (6)	B17^viii^—B20—B22—B13	−163.5 (3)
B9—B8—B10—Mg2^ii^	32.0 (4)	B23—B20—B22—B16	107.9 (4)
B2—B8—B10—Mg2^ii^	174.0 (3)	B21—B20—B22—B16	65.8 (4)
B7—B8—B10—Mg2^ii^	71.1 (5)	B20^viii^—B20—B22—B16	2.6 (5)
B8—B9—B10—B12	103.1 (4)	B18^viii^—B20—B22—B16	−125.7 (4)
B9^vi^—B9—B10—B12	−145.0 (3)	B19—B20—B22—B16	−34.1 (3)
B7—B9—B10—B12	64.7 (3)	B17^viii^—B20—B22—B16	−98.8 (3)
B11—B9—B10—B12	−32.7 (3)	B23—B20—B22—B14^i^	−109.9 (4)
B1—B9—B10—B12	1.3 (4)	B21—B20—B22—B14^i^	−152.0 (4)
Li2^ii^—B9—B10—B12	−102.5 (2)	B20^viii^—B20—B22—B14^i^	144.9 (4)
Mg2^ii^—B9—B10—B12	−102.5 (2)	B18^viii^—B20—B22—B14^i^	16.6 (6)
B8—B9—B10—B2	35.3 (4)	B19—B20—B22—B14^i^	108.1 (4)
B9^vi^—B9—B10—B2	147.3 (3)	B17^viii^—B20—B22—B14^i^	43.5 (4)
B7—B9—B10—B2	−3.1 (4)	B23—B20—B22—Mg1	−28.5 (3)
B11—B9—B10—B2	−100.4 (3)	B21—B20—B22—Mg1	−70.6 (4)
B1—B9—B10—B2	−66.4 (4)	B20^viii^—B20—B22—Mg1	−133.8 (3)
Li2^ii^—B9—B10—B2	−170.2 (2)	B18^viii^—B20—B22—Mg1	97.9 (5)
Mg2^ii^—B9—B10—B2	−170.2 (2)	B19—B20—B22—Mg1	−170.5 (4)
B9^vi^—B9—B10—B8	111.9 (5)	B17^viii^—B20—B22—Mg1	124.8 (4)
B7—B9—B10—B8	−38.4 (3)	B16^viii^—B19—B22—B20	88.9 (8)
B11—B9—B10—B8	−135.8 (4)	B16—B19—B22—B20	−143.1 (4)
B1—B9—B10—B8	−101.8 (4)	B22^viii^—B19—B22—B20	−19.1 (2)
Li2^ii^—B9—B10—B8	154.5 (3)	B20^viii^—B19—B22—B20	−34.6 (4)
Mg2^ii^—B9—B10—B8	154.5 (3)	B17^viii^—B19—B22—B20	67.8 (3)
B8—B9—B10—B4^ii^	−114.8 (4)	B17—B19—B22—B20	−98.3 (6)
B9^vi^—B9—B10—B4^ii^	−2.8 (5)	B16^viii^—B19—B22—B23	126.3 (8)
B7—B9—B10—B4^ii^	−153.2 (3)	B16—B19—B22—B23	−105.7 (4)
B11—B9—B10—B4^ii^	109.5 (3)	B22^viii^—B19—B22—B23	18.3 (3)
B1—B9—B10—B4^ii^	143.5 (3)	B20^viii^—B19—B22—B23	2.8 (4)
Li2^ii^—B9—B10—B4^ii^	39.7 (2)	B20—B19—B22—B23	37.4 (3)
Mg2^ii^—B9—B10—B4^ii^	39.7 (2)	B17^viii^—B19—B22—B23	105.2 (4)
B8—B9—B10—B11	135.8 (4)	B17—B19—B22—B23	−61.0 (7)
B9^vi^—B9—B10—B11	−112.3 (4)	B16^viii^—B19—B22—B13	−165.6 (7)
B7—B9—B10—B11	97.4 (3)	B16—B19—B22—B13	−37.6 (4)
B1—B9—B10—B11	34.0 (3)	B22^viii^—B19—B22—B13	86.4 (3)
Li2^ii^—B9—B10—B11	−69.75 (18)	B20^viii^—B19—B22—B13	71.0 (4)
Mg2^ii^—B9—B10—B11	−69.75 (18)	B20—B19—B22—B13	105.5 (3)
B8—B9—B10—Li2^ii^	−154.5 (3)	B17^viii^—B19—B22—B13	173.4 (3)
B9^vi^—B9—B10—Li2^ii^	−42.6 (3)	B17—B19—B22—B13	7.2 (7)
B7—B9—B10—Li2^ii^	167.1 (3)	B16^viii^—B19—B22—B16	−128.0 (11)
B11—B9—B10—Li2^ii^	69.75 (18)	B22^viii^—B19—B22—B16	124.0 (3)
B1—B9—B10—Li2^ii^	103.8 (3)	B20^viii^—B19—B22—B16	108.5 (2)
Mg2^ii^—B9—B10—Li2^ii^	0.0	B20—B19—B22—B16	143.1 (4)
B8—B9—B10—Mg2^ii^	−154.5 (3)	B17^viii^—B19—B22—B16	−149.1 (5)
B9^vi^—B9—B10—Mg2^ii^	−42.6 (3)	B17—B19—B22—B16	44.8 (4)
B7—B9—B10—Mg2^ii^	167.1 (3)	B16^viii^—B19—B22—B14^i^	−27.7 (11)
B11—B9—B10—Mg2^ii^	69.75 (18)	B16—B19—B22—B14^i^	100.2 (4)
B1—B9—B10—Mg2^ii^	103.8 (3)	B22^viii^—B19—B22—B14^i^	−135.7 (3)
Li2^ii^—B9—B10—Mg2^ii^	0.0	B20^viii^—B19—B22—B14^i^	−151.2 (3)
B1—B3—B11—B12	139.3 (3)	B20—B19—B22—B14^i^	−116.7 (5)
B5—B3—B11—B12	96.6 (3)	B17^viii^—B19—B22—B14^i^	−48.8 (5)
B15^i^—B3—B11—B12	−106.7 (4)	B17—B19—B22—B14^i^	145.0 (5)
B4—B3—B11—B12	34.6 (3)	B16^viii^—B19—B22—Mg1	−142 (2)
Mg1—B3—B11—B12	−32.8 (4)	B16—B19—B22—Mg1	−14 (3)
B12—B3—B11—B1	−139.3 (3)	B22^viii^—B19—B22—Mg1	110 (3)
B5—B3—B11—B1	−42.7 (3)	B20^viii^—B19—B22—Mg1	94 (3)
B15^i^—B3—B11—B1	114.0 (4)	B20—B19—B22—Mg1	129 (3)
B4—B3—B11—B1	−104.6 (3)	B17^viii^—B19—B22—Mg1	−163 (3)
Mg1—B3—B11—B1	−172.0 (4)	B17—B19—B22—Mg1	31 (3)
B1—B3—B11—B6^vii^	−110.3 (3)	B15—B13—B22—B20	58.6 (4)
B12—B3—B11—B6^vii^	110.5 (4)	B12—B13—B22—B20	−152.8 (4)
B5—B3—B11—B6^vii^	−152.9 (3)	B14—B13—B22—B20	−1.6 (5)
B15^i^—B3—B11—B6^vii^	3.7 (5)	B16—B13—B22—B20	105.9 (4)
B4—B3—B11—B6^vii^	145.1 (3)	B23—B13—B22—B20	−42.7 (3)
Mg1—B3—B11—B6^vii^	77.7 (4)	Mg1—B13—B22—B20	−116.0 (4)
B1—B3—B11—B10	103.1 (3)	B15—B13—B22—B23	101.3 (3)
B12—B3—B11—B10	−36.1 (3)	B12—B13—B22—B23	−110.1 (4)
B5—B3—B11—B10	60.5 (3)	B14—B13—B22—B23	41.0 (3)
B15^i^—B3—B11—B10	−142.9 (3)	B16—B13—B22—B23	148.6 (3)
B4—B3—B11—B10	−1.5 (4)	Mg1—B13—B22—B23	−73.4 (3)
Mg1—B3—B11—B10	−68.9 (4)	B15—B13—B22—B19	−9.9 (6)
B1—B3—B11—B9	37.8 (3)	B12—B13—B22—B19	138.7 (6)
B12—B3—B11—B9	−101.5 (4)	B14—B13—B22—B19	−70.2 (5)
B5—B3—B11—B9	−4.9 (4)	B16—B13—B22—B19	37.3 (5)
B15^i^—B3—B11—B9	151.8 (3)	B23—B13—B22—B19	−111.2 (5)
B4—B3—B11—B9	−66.8 (4)	Mg1—B13—B22—B19	175.4 (5)
Mg1—B3—B11—B9	−134.2 (3)	B15—B13—B22—B16	−47.3 (3)
B5—B1—B11—B3	42.9 (3)	B12—B13—B22—B16	101.4 (4)
B25^i^—B1—B11—B3	−95.0 (6)	B14—B13—B22—B16	−107.5 (4)
B7—B1—B11—B3	101.4 (3)	B23—B13—B22—B16	−148.6 (3)
B9—B1—B11—B3	140.2 (3)	Mg1—B13—B22—B16	138.1 (3)
B24^i^—B1—B11—B3	−74.7 (3)	B15—B13—B22—B14^i^	−147.0 (4)
B5—B1—B11—B12	3.8 (4)	B12—B13—B22—B14^i^	1.6 (5)
B3—B1—B11—B12	−39.1 (3)	B14—B13—B22—B14^i^	152.7 (4)
B25^i^—B1—B11—B12	−134.2 (5)	B16—B13—B22—B14^i^	−99.7 (4)
B7—B1—B11—B12	62.2 (4)	B23—B13—B22—B14^i^	111.7 (4)
B9—B1—B11—B12	101.0 (4)	Mg1—B13—B22—B14^i^	38.3 (3)
B24^i^—B1—B11—B12	−113.9 (3)	B15—B13—B22—Mg1	174.6 (4)
B5—B1—B11—B6^vii^	143.5 (3)	B12—B13—B22—Mg1	−36.7 (4)
B3—B1—B11—B6^vii^	100.6 (4)	B14—B13—B22—Mg1	114.4 (3)
B25^i^—B1—B11—B6^vii^	5.6 (7)	B16—B13—B22—Mg1	−138.1 (3)
B7—B1—B11—B6^vii^	−158.0 (3)	B23—B13—B22—Mg1	73.4 (3)
B9—B1—B11—B6^vii^	−119.2 (4)	B7^vii^—B16—B22—B20	151.5 (4)
B24^i^—B1—B11—B6^vii^	25.9 (4)	B19—B16—B22—B20	36.3 (5)
B5—B1—B11—B10	−61.0 (4)	B13—B16—B22—B20	−100.9 (4)
B3—B1—B11—B10	−103.9 (3)	B15—B16—B22—B20	−61.2 (4)
B25^i^—B1—B11—B10	161.1 (5)	B17—B16—B22—B20	−9.1 (4)
B7—B1—B11—B10	−2.5 (4)	B7^vii^—B16—B22—B23	−138.8 (4)
B9—B1—B11—B10	36.3 (3)	B19—B16—B22—B23	106.0 (5)
B24^i^—B1—B11—B10	−178.6 (3)	B13—B16—B22—B23	−31.3 (3)
B5—B1—B11—B9	−97.3 (3)	B15—B16—B22—B23	8.4 (4)
B3—B1—B11—B9	−140.2 (3)	B17—B16—B22—B23	60.6 (4)
B25^i^—B1—B11—B9	124.8 (6)	B7^vii^—B16—B22—B19	115.2 (6)
B7—B1—B11—B9	−38.8 (3)	B13—B16—B22—B19	−137.2 (5)
B24^i^—B1—B11—B9	145.1 (3)	B15—B16—B22—B19	−97.5 (5)
B5—B1—B11—Li2^ii^	−134.8 (3)	B17—B16—B22—B19	−45.4 (4)
B3—B1—B11—Li2^ii^	−177.7 (4)	B7^vii^—B16—B22—B13	−107.6 (5)
B25^i^—B1—B11—Li2^ii^	87.3 (6)	B19—B16—B22—B13	137.2 (5)
B7—B1—B11—Li2^ii^	−76.3 (4)	B15—B16—B22—B13	39.7 (3)
B9—B1—B11—Li2^ii^	−37.5 (3)	B17—B16—B22—B13	91.8 (3)
B24^i^—B1—B11—Li2^ii^	107.6 (3)	B7^vii^—B16—B22—B14^i^	4.8 (6)
B5—B1—B11—Mg2^ii^	−134.8 (3)	B19—B16—B22—B14^i^	−110.5 (5)
B3—B1—B11—Mg2^ii^	−177.7 (4)	B13—B16—B22—B14^i^	112.3 (4)
B25^i^—B1—B11—Mg2^ii^	87.3 (6)	B15—B16—B22—B14^i^	152.0 (3)
B7—B1—B11—Mg2^ii^	−76.3 (4)	B17—B16—B22—B14^i^	−155.8 (3)
B9—B1—B11—Mg2^ii^	−37.5 (3)	B7^vii^—B16—B22—Mg1	−67.5 (5)
B24^i^—B1—B11—Mg2^ii^	107.6 (3)	B19—B16—B22—Mg1	177.3 (5)
B12—B10—B11—B3	37.8 (3)	B13—B16—B22—Mg1	40.1 (3)
B2—B10—B11—B3	0.6 (4)	B15—B16—B22—Mg1	79.8 (3)
B8—B10—B11—B3	−64.2 (4)	B17—B16—B22—Mg1	131.9 (3)
B4^ii^—B10—B11—B3	141.4 (3)	B19—B22—B23—B20	−38.2 (3)
B9—B10—B11—B3	−103.2 (3)	B13—B22—B23—B20	−134.4 (3)
Li1^ii^—B10—B11—B3	69.4 (3)	B16—B22—B23—B20	−104.4 (4)
Mg1^ii^—B10—B11—B3	69.4 (3)	B14^i^—B22—B23—B20	115.2 (4)
Li2^ii^—B10—B11—B3	170.7 (3)	Mg1—B22—B23—B20	153.3 (3)
Mg2^ii^—B10—B11—B3	170.7 (3)	B20—B22—B23—B13	134.4 (3)
B2—B10—B11—B12	−37.2 (3)	B19—B22—B23—B13	96.2 (3)
B8—B10—B11—B12	−102.0 (4)	B16—B22—B23—B13	29.9 (3)
B4^ii^—B10—B11—B12	103.6 (3)	B14^i^—B22—B23—B13	−110.4 (4)
B9—B10—B11—B12	−141.0 (3)	Mg1—B22—B23—B13	−72.3 (2)
Li1^ii^—B10—B11—B12	31.6 (3)	B20—B22—B23—B2^i^	−115.9 (4)
Mg1^ii^—B10—B11—B12	31.6 (3)	B19—B22—B23—B2^i^	−154.1 (3)
Li2^ii^—B10—B11—B12	132.9 (3)	B13—B22—B23—B2^i^	109.7 (4)
Mg2^ii^—B10—B11—B12	132.9 (3)	B16—B22—B23—B2^i^	139.6 (4)
B12—B10—B11—B1	103.6 (3)	B14^i^—B22—B23—B2^i^	−0.7 (5)
B2—B10—B11—B1	66.5 (4)	Mg1—B22—B23—B2^i^	37.4 (3)
B8—B10—B11—B1	1.6 (4)	B20—B22—B23—B21	39.6 (3)
B4^ii^—B10—B11—B1	−152.7 (3)	B19—B22—B23—B21	1.5 (4)
B9—B10—B11—B1	−37.4 (3)	B13—B22—B23—B21	−94.7 (3)
Li1^ii^—B10—B11—B1	135.3 (3)	B16—B22—B23—B21	−64.8 (4)
Mg1^ii^—B10—B11—B1	135.3 (3)	B14^i^—B22—B23—B21	154.8 (3)
Li2^ii^—B10—B11—B1	−123.4 (3)	Mg1—B22—B23—B21	−167.0 (3)
Mg2^ii^—B10—B11—B1	−123.4 (3)	B20—B22—B23—B14	97.9 (3)
B12—B10—B11—B6^vii^	−103.2 (4)	B19—B22—B23—B14	59.7 (4)
B2—B10—B11—B6^vii^	−140.4 (3)	B13—B22—B23—B14	−36.5 (3)
B8—B10—B11—B6^vii^	154.8 (4)	B16—B22—B23—B14	−6.6 (4)
B4^ii^—B10—B11—B6^vii^	0.4 (5)	B14^i^—B22—B23—B14	−146.9 (4)
B9—B10—B11—B6^vii^	115.8 (4)	Mg1—B22—B23—B14	−108.8 (3)
Li1^ii^—B10—B11—B6^vii^	−71.6 (4)	B20—B22—B23—Mg1	−153.3 (3)
Mg1^ii^—B10—B11—B6^vii^	−71.6 (4)	B19—B22—B23—Mg1	168.5 (4)
Li2^ii^—B10—B11—B6^vii^	29.7 (3)	B13—B22—B23—Mg1	72.3 (2)
Mg2^ii^—B10—B11—B6^vii^	29.7 (3)	B16—B22—B23—Mg1	102.2 (3)
B12—B10—B11—B9	141.0 (3)	B14^i^—B22—B23—Mg1	−38.1 (3)
B2—B10—B11—B9	103.8 (3)	B21^viii^—B20—B23—B22	170.5 (5)
B8—B10—B11—B9	39.0 (3)	B21—B20—B23—B22	135.0 (3)
B4^ii^—B10—B11—B9	−115.4 (3)	B20^viii^—B20—B23—B22	101.8 (3)
Li1^ii^—B10—B11—B9	172.6 (3)	B18^viii^—B20—B23—B22	−108.3 (5)
Mg1^ii^—B10—B11—B9	172.6 (3)	B19—B20—B23—B22	36.0 (3)
Li2^ii^—B10—B11—B9	−86.1 (2)	B17^viii^—B20—B23—B22	−33.7 (4)
Mg2^ii^—B10—B11—B9	−86.1 (2)	B22—B20—B23—B13	−41.2 (3)
B12—B10—B11—Li2^ii^	−132.9 (3)	B21^viii^—B20—B23—B13	129.3 (4)
B2—B10—B11—Li2^ii^	−170.1 (3)	B21—B20—B23—B13	93.8 (3)
B8—B10—B11—Li2^ii^	125.1 (3)	B20^viii^—B20—B23—B13	60.6 (3)
B4^ii^—B10—B11—Li2^ii^	−29.3 (3)	B18^viii^—B20—B23—B13	−149.5 (4)
B9—B10—B11—Li2^ii^	86.1 (2)	B19—B20—B23—B13	−5.2 (4)
Li1^ii^—B10—B11—Li2^ii^	−101.3 (3)	B17^viii^—B20—B23—B13	−74.9 (5)
Mg1^ii^—B10—B11—Li2^ii^	−101.3 (3)	B22—B20—B23—B2^i^	105.3 (4)
Mg2^ii^—B10—B11—Li2^ii^	0.0	B21^viii^—B20—B23—B2^i^	−84.3 (5)
B12—B10—B11—Mg2^ii^	−132.9 (3)	B21—B20—B23—B2^i^	−119.7 (4)
B2—B10—B11—Mg2^ii^	−170.1 (3)	B20^viii^—B20—B23—B2^i^	−152.9 (3)
B8—B10—B11—Mg2^ii^	125.1 (3)	B18^viii^—B20—B23—B2^i^	−3.0 (7)
B4^ii^—B10—B11—Mg2^ii^	−29.3 (3)	B19—B20—B23—B2^i^	141.3 (4)
B9—B10—B11—Mg2^ii^	86.1 (2)	B17^viii^—B20—B23—B2^i^	71.6 (6)
Li1^ii^—B10—B11—Mg2^ii^	−101.3 (3)	B22—B20—B23—B21	−135.0 (3)
Mg1^ii^—B10—B11—Mg2^ii^	−101.3 (3)	B21^viii^—B20—B23—B21	35.4 (4)
Li2^ii^—B10—B11—Mg2^ii^	0.0	B20^viii^—B20—B23—B21	−33.2 (2)
B8—B9—B11—B3	65.4 (4)	B18^viii^—B20—B23—B21	116.7 (5)
B9^vi^—B9—B11—B3	−156.0 (4)	B19—B20—B23—B21	−99.0 (4)
B7—B9—B11—B3	2.9 (4)	B17^viii^—B20—B23—B21	−168.7 (5)
B10—B9—B11—B3	103.7 (3)	B22—B20—B23—B14	−98.3 (3)
B1—B9—B11—B3	−36.9 (3)	B21^viii^—B20—B23—B14	72.2 (5)
Li2^ii^—B9—B11—B3	172.8 (3)	B21—B20—B23—B14	36.7 (3)
Mg2^ii^—B9—B11—B3	172.8 (3)	B20^viii^—B20—B23—B14	3.5 (3)
B8—B9—B11—B12	−3.2 (4)	B18^viii^—B20—B23—B14	153.4 (4)
B9^vi^—B9—B11—B12	135.4 (4)	B19—B20—B23—B14	−62.3 (4)
B7—B9—B11—B12	−65.7 (4)	B17^viii^—B20—B23—B14	−132.0 (4)
B10—B9—B11—B12	35.1 (3)	B22—B20—B23—Mg1	28.1 (3)
B1—B9—B11—B12	−105.5 (3)	B21^viii^—B20—B23—Mg1	−161.5 (3)
Li2^ii^—B9—B11—B12	104.2 (3)	B21—B20—B23—Mg1	163.1 (4)
Mg2^ii^—B9—B11—B12	104.2 (3)	B20^viii^—B20—B23—Mg1	129.9 (2)
B8—B9—B11—B1	102.3 (4)	B18^viii^—B20—B23—Mg1	−80.2 (5)
B9^vi^—B9—B11—B1	−119.1 (5)	B19—B20—B23—Mg1	64.1 (4)
B7—B9—B11—B1	39.8 (3)	B17^viii^—B20—B23—Mg1	−5.6 (6)
B10—B9—B11—B1	140.6 (3)	B15—B13—B23—B22	−96.7 (4)
Li2^ii^—B9—B11—B1	−150.3 (3)	B12—B13—B23—B22	109.0 (4)
Mg2^ii^—B9—B11—B1	−150.3 (3)	B14—B13—B23—B22	−135.3 (3)
B8—B9—B11—B6^vii^	−152.4 (4)	B16—B13—B23—B22	−28.7 (3)
B9^vi^—B9—B11—B6^vii^	−13.8 (6)	Li1^ii^—B13—B23—B22	−170.2 (4)
B7—B9—B11—B6^vii^	145.1 (4)	Mg1^ii^—B13—B23—B22	−170.2 (4)
B10—B9—B11—B6^vii^	−114.1 (4)	Mg1—B13—B23—B22	75.8 (3)
B1—B9—B11—B6^vii^	105.3 (4)	B15—B13—B23—B20	−57.8 (4)
Li2^ii^—B9—B11—B6^vii^	−45.0 (3)	B12—B13—B23—B20	147.9 (4)
Mg2^ii^—B9—B11—B6^vii^	−45.0 (3)	B14—B13—B23—B20	−96.4 (3)
B8—B9—B11—B10	−38.3 (3)	B16—B13—B23—B20	10.2 (4)
B9^vi^—B9—B11—B10	100.3 (4)	B22—B13—B23—B20	38.9 (3)
B7—B9—B11—B10	−100.8 (3)	Li1^ii^—B13—B23—B20	−131.3 (3)
B1—B9—B11—B10	−140.6 (3)	Mg1^ii^—B13—B23—B20	−131.3 (3)
Li2^ii^—B9—B11—B10	69.13 (17)	Mg1—B13—B23—B20	114.8 (3)
Mg2^ii^—B9—B11—B10	69.13 (17)	B15—B13—B23—B2^i^	153.1 (3)
B8—B9—B11—Li2^ii^	−107.4 (3)	B12—B13—B23—B2^i^	−1.2 (5)
B9^vi^—B9—B11—Li2^ii^	31.1 (4)	B14—B13—B23—B2^i^	114.5 (4)
B7—B9—B11—Li2^ii^	−170.0 (3)	B16—B13—B23—B2^i^	−138.9 (3)
B10—B9—B11—Li2^ii^	−69.13 (17)	B22—B13—B23—B2^i^	−110.2 (4)
B1—B9—B11—Li2^ii^	150.3 (3)	Li1^ii^—B13—B23—B2^i^	79.6 (4)
Mg2^ii^—B9—B11—Li2^ii^	0.000 (1)	Mg1^ii^—B13—B23—B2^i^	79.6 (4)
B8—B9—B11—Mg2^ii^	−107.4 (3)	Mg1—B13—B23—B2^i^	−34.4 (3)
B9^vi^—B9—B11—Mg2^ii^	31.1 (4)	B15—B13—B23—B21	4.6 (4)
B7—B9—B11—Mg2^ii^	−170.0 (3)	B12—B13—B23—B21	−149.7 (4)
B10—B9—B11—Mg2^ii^	−69.13 (17)	B14—B13—B23—B21	−34.0 (3)
B1—B9—B11—Mg2^ii^	150.3 (3)	B16—B13—B23—B21	72.6 (4)
Li2^ii^—B9—B11—Mg2^ii^	0.000 (1)	B22—B13—B23—B21	101.3 (3)
B3—B11—B12—B10	−140.7 (3)	Li1^ii^—B13—B23—B21	−68.9 (4)
B1—B11—B12—B10	−102.9 (3)	Mg1^ii^—B13—B23—B21	−68.9 (4)
B6^vii^—B11—B12—B10	117.2 (3)	Mg1—B13—B23—B21	177.1 (3)
B9—B11—B12—B10	−35.3 (3)	B15—B13—B23—B14	38.6 (3)
Li2^ii^—B11—B12—B10	44.0 (2)	B12—B13—B23—B14	−115.7 (4)
Mg2^ii^—B11—B12—B10	44.0 (2)	B16—B13—B23—B14	106.6 (4)
B3—B11—B12—B13	102.2 (4)	B22—B13—B23—B14	135.3 (3)
B1—B11—B12—B13	140.1 (4)	Li1^ii^—B13—B23—B14	−34.9 (3)
B6^vii^—B11—B12—B13	0.2 (6)	Mg1^ii^—B13—B23—B14	−34.9 (3)
B10—B11—B12—B13	−117.1 (4)	Mg1—B13—B23—B14	−148.9 (3)
B9—B11—B12—B13	−152.4 (4)	B15—B13—B23—Mg1	−172.5 (4)
Li2^ii^—B11—B12—B13	−73.0 (5)	B12—B13—B23—Mg1	33.1 (3)
Mg2^ii^—B11—B12—B13	−73.0 (5)	B14—B13—B23—Mg1	148.9 (3)
B1—B11—B12—B3	37.8 (3)	B16—B13—B23—Mg1	−104.6 (3)
B6^vii^—B11—B12—B3	−102.1 (4)	B22—B13—B23—Mg1	−75.8 (3)
B10—B11—B12—B3	140.7 (3)	Li1^ii^—B13—B23—Mg1	114.0 (4)
B9—B11—B12—B3	105.4 (3)	Mg1^ii^—B13—B23—Mg1	114.0 (4)
Li2^ii^—B11—B12—B3	−175.3 (3)	B24^x^—B21—B23—B22	−160.8 (3)
Mg2^ii^—B11—B12—B3	−175.3 (3)	B14—B21—B23—B22	97.5 (3)
B3—B11—B12—B4	−38.8 (3)	B25^xi^—B21—B23—B22	−119.3 (4)
B1—B11—B12—B4	−1.0 (4)	B20^viii^—B21—B23—B22	−5.4 (4)
B6^vii^—B11—B12—B4	−140.9 (3)	B20—B21—B23—B22	−38.2 (3)
B10—B11—B12—B4	101.9 (3)	B18—B21—B23—B22	60.5 (4)
B9—B11—B12—B4	66.6 (4)	B21^viii^—B21—B23—B22	−62.7 (3)
Li2^ii^—B11—B12—B4	145.9 (3)	B24^x^—B21—B23—B20	−122.6 (3)
Mg2^ii^—B11—B12—B4	145.9 (3)	B14—B21—B23—B20	135.7 (3)
B3—B11—B12—B2	−105.1 (4)	B25^xi^—B21—B23—B20	−81.1 (4)
B1—B11—B12—B2	−67.3 (4)	B20^viii^—B21—B23—B20	32.8 (3)
B6^vii^—B11—B12—B2	152.8 (3)	B18—B21—B23—B20	98.7 (4)
B10—B11—B12—B2	35.6 (3)	B21^viii^—B21—B23—B20	−24.5 (2)
B9—B11—B12—B2	0.3 (4)	B24^x^—B21—B23—B13	135.2 (3)
Li2^ii^—B11—B12—B2	79.6 (3)	B14—B21—B23—B13	33.5 (3)
Mg2^ii^—B11—B12—B2	79.6 (3)	B25^xi^—B21—B23—B13	176.7 (3)
B3—B11—B12—Li1^ii^	−176.4 (4)	B20^viii^—B21—B23—B13	−69.4 (4)
B1—B11—B12—Li1^ii^	−138.5 (3)	B20—B21—B23—B13	−102.3 (3)
B6^vii^—B11—B12—Li1^ii^	81.6 (4)	B18—B21—B23—B13	−3.6 (4)
B10—B11—B12—Li1^ii^	−35.7 (3)	B21^viii^—B21—B23—B13	−126.8 (2)
B9—B11—B12—Li1^ii^	−71.0 (4)	B24^x^—B21—B23—B2^i^	−8.6 (5)
Li2^ii^—B11—B12—Li1^ii^	8.4 (4)	B14—B21—B23—B2^i^	−110.3 (4)
Mg2^ii^—B11—B12—Li1^ii^	8.4 (4)	B25^xi^—B21—B23—B2^i^	32.9 (6)
B3—B11—B12—Mg1^ii^	−176.4 (4)	B20^viii^—B21—B23—B2^i^	146.8 (4)
B1—B11—B12—Mg1^ii^	−138.5 (3)	B20—B21—B23—B2^i^	114.0 (4)
B6^vii^—B11—B12—Mg1^ii^	81.6 (4)	B18—B21—B23—B2^i^	−147.3 (4)
B10—B11—B12—Mg1^ii^	−35.7 (3)	B21^viii^—B21—B23—B2^i^	89.5 (4)
B9—B11—B12—Mg1^ii^	−71.0 (4)	B24^x^—B21—B23—B14	101.7 (3)
Li2^ii^—B11—B12—Mg1^ii^	8.4 (4)	B25^xi^—B21—B23—B14	143.2 (4)
Mg2^ii^—B11—B12—Mg1^ii^	8.4 (4)	B20^viii^—B21—B23—B14	−102.9 (4)
B3—B11—B12—Mg1	29.4 (3)	B20—B21—B23—B14	−135.7 (3)
B1—B11—B12—Mg1	67.2 (4)	B18—B21—B23—B14	−37.0 (3)
B6^vii^—B11—B12—Mg1	−72.7 (4)	B21^viii^—B21—B23—B14	−160.3 (3)
B10—B11—B12—Mg1	170.0 (4)	B24^x^—B21—B23—Mg1	145.3 (12)
B9—B11—B12—Mg1	134.7 (3)	B14—B21—B23—Mg1	43.6 (13)
Li2^ii^—B11—B12—Mg1	−145.9 (3)	B25^xi^—B21—B23—Mg1	−173.1 (11)
Mg2^ii^—B11—B12—Mg1	−145.9 (3)	B20^viii^—B21—B23—Mg1	−59.3 (13)
B2—B10—B12—B11	140.5 (3)	B20—B21—B23—Mg1	−92.1 (13)
B8—B10—B12—B11	97.5 (3)	B18—B21—B23—Mg1	6.6 (14)
B4^ii^—B10—B12—B11	−111.0 (3)	B21^viii^—B21—B23—Mg1	−116.6 (12)
B9—B10—B12—B11	34.6 (3)	B15—B17—B24—B25	−94.3 (8)
Li1^ii^—B10—B12—B11	−147.5 (3)	B18—B17—B24—B25	−155.2 (7)
Mg1^ii^—B10—B12—B11	−147.5 (3)	B16—B17—B24—B25	−31.2 (8)
Li2^ii^—B10—B12—B11	−43.6 (2)	B19—B17—B24—B25	20.4 (10)
Mg2^ii^—B10—B12—B11	−43.6 (2)	B20^viii^—B17—B24—B25	120.2 (10)
B2—B10—B12—B13	−101.1 (4)	B15—B17—B24—B21^xii^	−170.9 (3)
B8—B10—B12—B13	−144.0 (4)	B18—B17—B24—B21^xii^	128.1 (4)
B4^ii^—B10—B12—B13	7.4 (5)	B16—B17—B24—B21^xii^	−107.9 (4)
B11—B10—B12—B13	118.4 (4)	B19—B17—B24—B21^xii^	−56.3 (5)
B9—B10—B12—B13	153.0 (4)	B20^viii^—B17—B24—B21^xii^	43.6 (10)
Li1^ii^—B10—B12—B13	−29.0 (3)	B15—B17—B24—B1^ii^	1.1 (5)
Mg1^ii^—B10—B12—B13	−29.0 (3)	B18—B17—B24—B1^ii^	−59.9 (6)
Li2^ii^—B10—B12—B13	74.8 (4)	B16—B17—B24—B1^ii^	64.2 (5)
Mg2^ii^—B10—B12—B13	74.8 (4)	B19—B17—B24—B1^ii^	115.7 (4)
B2—B10—B12—B3	105.7 (3)	B20^viii^—B17—B24—B1^ii^	−144.4 (7)
B8—B10—B12—B3	62.7 (4)	B17—B24—B25—B21^xii^	−90.6 (8)
B4^ii^—B10—B12—B3	−145.9 (3)	B1^ii^—B24—B25—B21^xii^	151.4 (3)
B11—B10—B12—B3	−34.9 (3)	B21^xii^—B24—B25—B21^xiii^	−43.1 (4)
B9—B10—B12—B3	−0.3 (4)	B17—B24—B25—B21^xiii^	−133.7 (6)
Li1^ii^—B10—B12—B3	177.7 (4)	B1^ii^—B24—B25—B21^xiii^	108.3 (2)
Mg1^ii^—B10—B12—B3	177.7 (4)	B21^xii^—B24—B25—B1^ii^	−151.4 (3)
Li2^ii^—B10—B12—B3	−78.5 (3)	B17—B24—B25—B1^ii^	118.0 (7)
Mg2^ii^—B10—B12—B3	−78.5 (3)	B21^xii^—B24—B25—B1^vii^	107.4 (3)
B2—B10—B12—B4	35.6 (3)	B17—B24—B25—B1^vii^	16.8 (9)
B8—B10—B12—B4	−7.4 (4)	B1^ii^—B24—B25—B1^vii^	−101.2 (5)
B4^ii^—B10—B12—B4	144.0 (3)	B1—B5—Mg2—B5^iii^	−26.7 (13)
B11—B10—B12—B4	−104.9 (3)	B7—B5—Mg2—B5^iii^	105.6 (4)
B9—B10—B12—B4	−70.3 (3)	B3—B5—Mg2—B5^iii^	−104.3 (4)
Li1^ii^—B10—B12—B4	107.6 (3)	B4—B5—Mg2—B5^iii^	−148.6 (3)
Mg1^ii^—B10—B12—B4	107.6 (3)	B6—B5—Mg2—B5^iii^	148.4 (3)
Li2^ii^—B10—B12—B4	−148.6 (2)	B1—B5—Mg2—B4	121.9 (15)
Mg2^ii^—B10—B12—B4	−148.6 (2)	B7—B5—Mg2—B4	−105.8 (3)
B8—B10—B12—B2	−43.0 (3)	B3—B5—Mg2—B4	44.3 (2)
B4^ii^—B10—B12—B2	108.4 (3)	B5^iii^—B5—Mg2—B4	148.6 (3)
B11—B10—B12—B2	−140.5 (3)	B6—B5—Mg2—B4	−63.0 (2)
B9—B10—B12—B2	−105.9 (3)	B1—B5—Mg2—B4^iii^	−53.3 (15)
Li1^ii^—B10—B12—B2	72.0 (2)	B7—B5—Mg2—B4^iii^	79.0 (3)
Mg1^ii^—B10—B12—B2	72.0 (2)	B3—B5—Mg2—B4^iii^	−130.9 (3)
Li2^ii^—B10—B12—B2	175.8 (3)	B5^iii^—B5—Mg2—B4^iii^	−26.6 (3)
Mg2^ii^—B10—B12—B2	175.8 (3)	B4—B5—Mg2—B4^iii^	−175.21 (9)
B2—B10—B12—Li1^ii^	−72.0 (2)	B6—B5—Mg2—B4^iii^	121.8 (2)
B8—B10—B12—Li1^ii^	−115.0 (3)	B1—B5—Mg2—B10^iv^	−107.1 (14)
B4^ii^—B10—B12—Li1^ii^	36.4 (2)	B7—B5—Mg2—B10^iv^	25.1 (4)
B11—B10—B12—Li1^ii^	147.5 (3)	B3—B5—Mg2—B10^iv^	175.2 (2)
B9—B10—B12—Li1^ii^	−178.0 (3)	B5^iii^—B5—Mg2—B10^iv^	−80.5 (3)
Mg1^ii^—B10—B12—Li1^ii^	0.000 (1)	B4—B5—Mg2—B10^iv^	130.9 (2)
Li2^ii^—B10—B12—Li1^ii^	103.8 (2)	B6—B5—Mg2—B10^iv^	67.9 (3)
Mg2^ii^—B10—B12—Li1^ii^	103.8 (2)	B1—B5—Mg2—B10^i^	98.1 (15)
B2—B10—B12—Mg1^ii^	−72.0 (2)	B7—B5—Mg2—B10^i^	−129.6 (3)
B8—B10—B12—Mg1^ii^	−115.0 (3)	B3—B5—Mg2—B10^i^	20.4 (3)
B4^ii^—B10—B12—Mg1^ii^	36.4 (2)	B5^iii^—B5—Mg2—B10^i^	124.8 (2)
B11—B10—B12—Mg1^ii^	147.5 (3)	B4—B5—Mg2—B10^i^	−23.83 (16)
B9—B10—B12—Mg1^ii^	−178.0 (3)	B6—B5—Mg2—B10^i^	−86.8 (2)
Li1^ii^—B10—B12—Mg1^ii^	0.000 (1)	B1—B5—Mg2—B11^iv^	−152.8 (15)
Li2^ii^—B10—B12—Mg1^ii^	103.8 (2)	B7—B5—Mg2—B11^iv^	−20.5 (3)
Mg2^ii^—B10—B12—Mg1^ii^	103.8 (2)	B3—B5—Mg2—B11^iv^	129.6 (3)
B1—B3—B12—B11	−38.9 (3)	B5^iii^—B5—Mg2—B11^iv^	−126.1 (2)
B5—B3—B12—B11	−100.2 (3)	B4—B5—Mg2—B11^iv^	85.31 (18)
B15^i^—B3—B12—B11	109.7 (4)	B6—B5—Mg2—B11^iv^	22.3 (2)
B4—B3—B12—B11	−139.9 (3)	B1—B5—Mg2—B11^i^	49.6 (15)
Mg1—B3—B12—B11	149.9 (3)	B7—B5—Mg2—B11^i^	−178.1 (3)
B11—B3—B12—B10	37.9 (3)	B3—B5—Mg2—B11^i^	−28.0 (4)
B1—B3—B12—B10	−1.0 (4)	B5^iii^—B5—Mg2—B11^i^	76.3 (3)
B5—B3—B12—B10	−62.3 (4)	B4—B5—Mg2—B11^i^	−72.3 (2)
B15^i^—B3—B12—B10	147.6 (3)	B6—B5—Mg2—B11^i^	−135.3 (3)
B4—B3—B12—B10	−102.0 (3)	B1—B5—Mg2—B6^iii^	−1.0 (15)
Mg1—B3—B12—B10	−172.2 (3)	B7—B5—Mg2—B6^iii^	131.3 (3)
B11—B3—B12—B13	−117.8 (4)	B3—B5—Mg2—B6^iii^	−78.7 (3)
B1—B3—B12—B13	−156.7 (4)	B5^iii^—B5—Mg2—B6^iii^	25.7 (3)
B5—B3—B12—B13	142.0 (3)	B4—B5—Mg2—B6^iii^	−122.94 (18)
B15^i^—B3—B12—B13	−8.1 (5)	B6—B5—Mg2—B6^iii^	174.04 (11)
B4—B3—B12—B13	102.3 (4)	B1—B5—Mg2—B6	−175.1 (15)
Mg1—B3—B12—B13	32.1 (3)	B7—B5—Mg2—B6	−42.8 (3)
B11—B3—B12—B4	139.9 (3)	B3—B5—Mg2—B6	107.3 (3)
B1—B3—B12—B4	101.0 (3)	B5^iii^—B5—Mg2—B6	−148.4 (3)
B5—B3—B12—B4	39.7 (3)	B4—B5—Mg2—B6	63.0 (2)
B15^i^—B3—B12—B4	−110.4 (4)	B1—B5—Mg2—B9^iv^	−169.7 (14)
Mg1—B3—B12—B4	−70.2 (2)	B7—B5—Mg2—B9^iv^	−37.4 (5)
B11—B3—B12—B2	101.4 (4)	B3—B5—Mg2—B9^iv^	112.7 (3)
B1—B3—B12—B2	62.5 (4)	B5^iii^—B5—Mg2—B9^iv^	−143.0 (3)
B5—B3—B12—B2	1.2 (4)	B4—B5—Mg2—B9^iv^	68.4 (3)
B15^i^—B3—B12—B2	−148.9 (4)	B6—B5—Mg2—B9^iv^	5.4 (4)
B4—B3—B12—B2	−38.5 (3)	B1—B5—Mg2—B9^i^	111.7 (15)
Mg1—B3—B12—B2	−108.7 (3)	B7—B5—Mg2—B9^i^	−116.0 (3)
B11—B3—B12—Mg1	−149.9 (3)	B3—B5—Mg2—B9^i^	34.0 (5)
B1—B3—B12—Mg1	171.2 (4)	B5^iii^—B5—Mg2—B9^i^	138.4 (3)
B5—B3—B12—Mg1	109.9 (3)	B4—B5—Mg2—B9^i^	−10.2 (4)
B15^i^—B3—B12—Mg1	−40.2 (3)	B6—B5—Mg2—B9^i^	−73.3 (4)

Symmetry codes: (i) *y*−1/2, −*x*+1/2, *z*+1/4; (ii) −*y*+1/2, *x*+1/2, *z*−1/4; (iii) −*y*, −*x*, −*z*+1/2; (iv) *x*−1/2, −*y*+1/2, −*z*+1/4; (v) *y*−1, *x*, −*z*; (vi) *y*, *x*, −*z*; (vii) *x*+1/2, −*y*+1/2, −*z*+1/4; (viii) −*y*+1, −*x*+1, −*z*+1/2; (ix) *y*, *x*+1, −*z*; (x) *x*−1/2, −*y*+3/2, −*z*+1/4; (xi) *y*−1/2, −*x*+3/2, *z*+1/4; (xii) *x*+1/2, −*y*+3/2, −*z*+1/4; (xiii) −*y*+3/2, *x*+1/2, *z*−1/4.
